# Supplementary material for: Transcriptomic Investigation of FoxM1-Mediated Neuroprotection by hAEC-Derived Exosomes in an In Vitro Ischemic Stroke Model
Source: Biology (Basel). 2025 Oct 7;14(10):1368. doi: 10.3390/biology14101368 (PMC12561972; doi:10.3390/biology14101368)
Supplement: Supplementary file 1 [file biology-14-01368-s001.zip › biology-3845635-supplementary.pdf]

First replicate

Figure1D

Figure1D-HT22-Bax

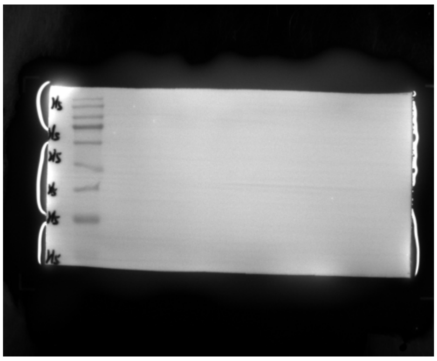 Full membrane image	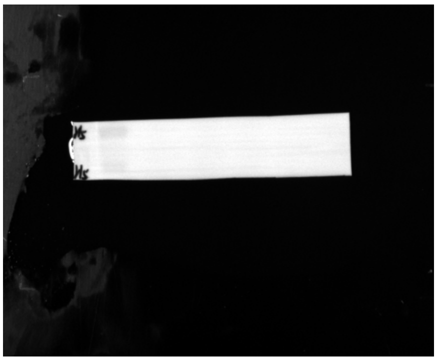 White light image
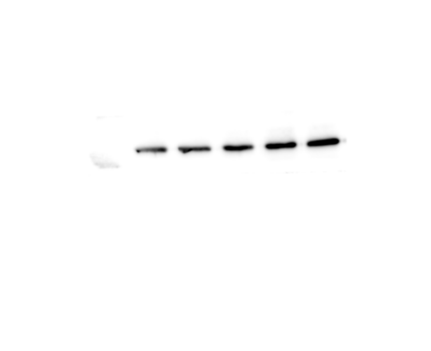 Chemiluminescent image	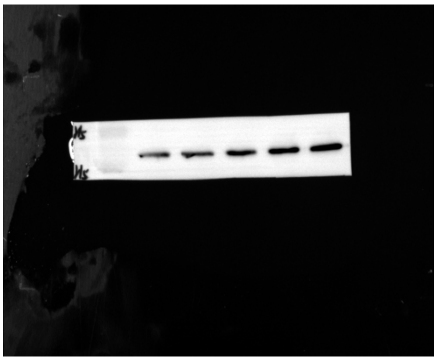 Composite image

Figure1D-HT22-Bcl2

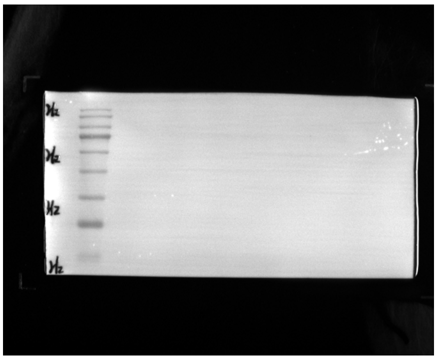 Full membrane image	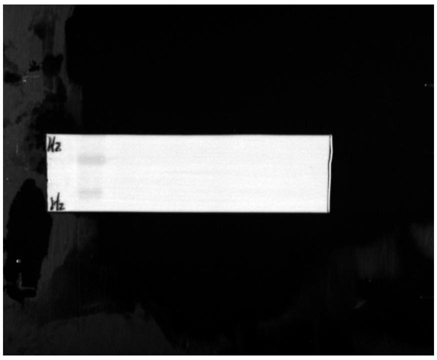 White light image
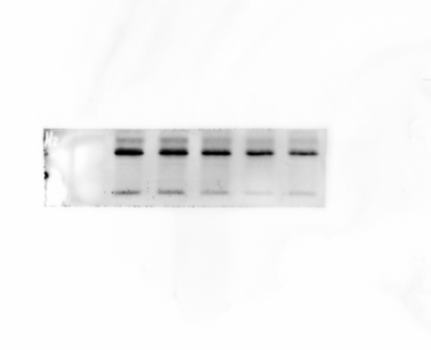	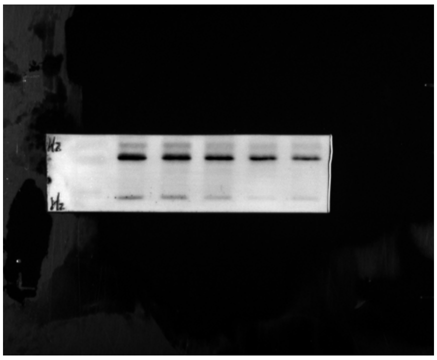

Chemiluminescent image	Composite image

Figure1D-HT22-Caspase3

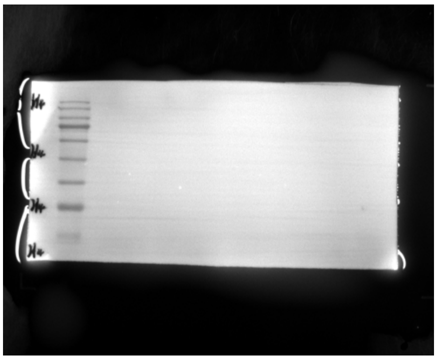<br>Full membrane image	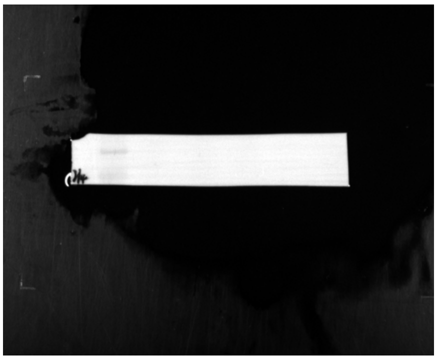<br>White light image
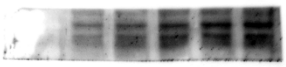<br>Chemiluminescent image	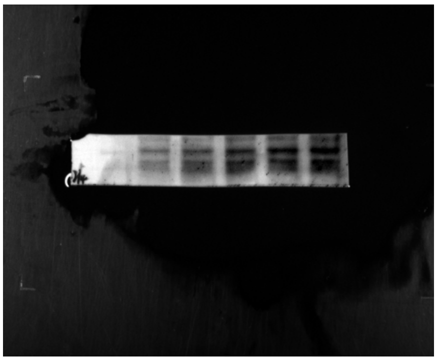<br>Composite image

Figure1D-HT22-BDNF

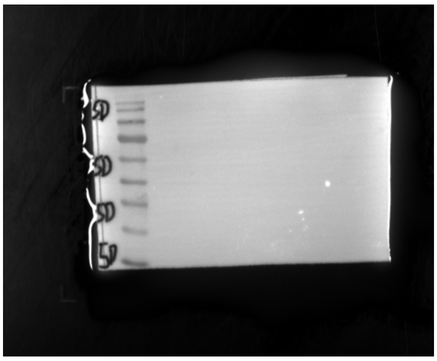<br>Full membrane image	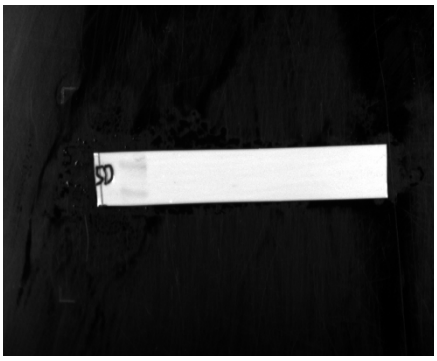<br>White light image
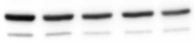	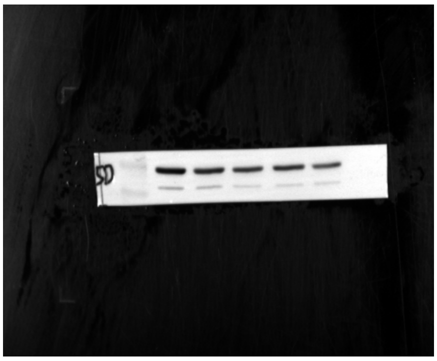

Chemiluminescent image	Composite image

Figure1D-HT22-β-actin

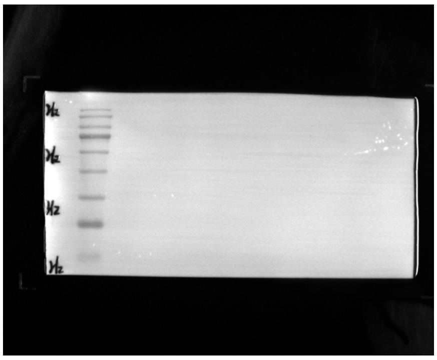	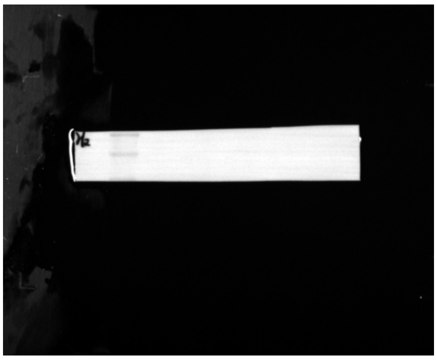
Full membrane image-同 Bcl2	White light image
	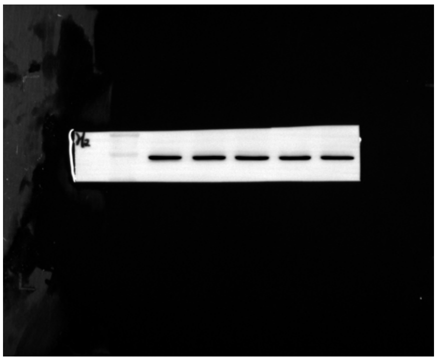
Chemiluminescent image	Composite image

Figure1D-BV2-Bax

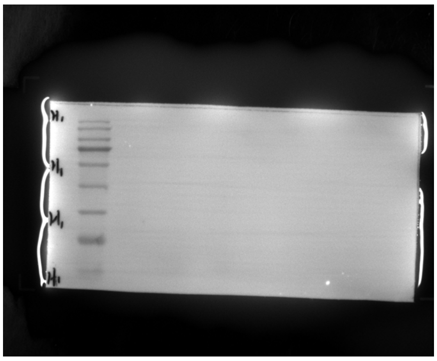	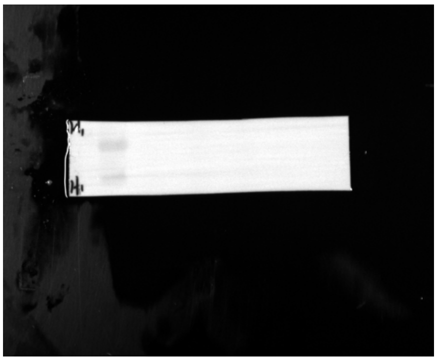
Full membrane image	White light image
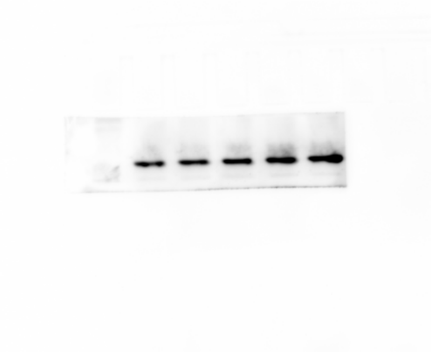	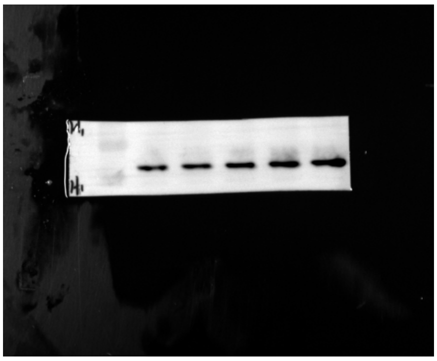

Chemiluminescent image	Composite image

Figure1D-BV2-Bcl2

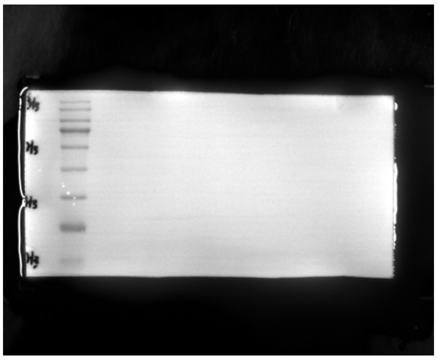	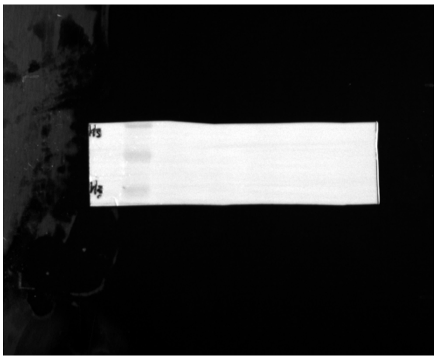
Full membrane image	White light image
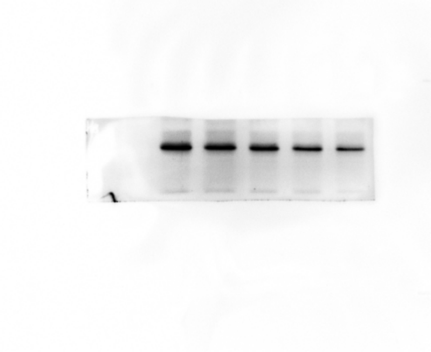	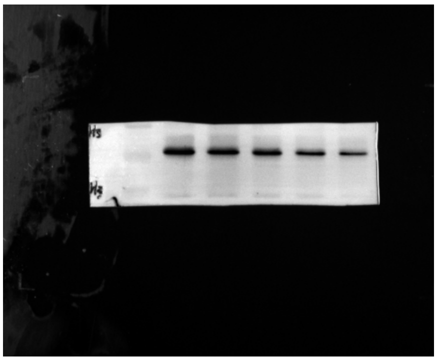
Chemiluminescent image	Composite image

Figure1D-BV2-Caspase3

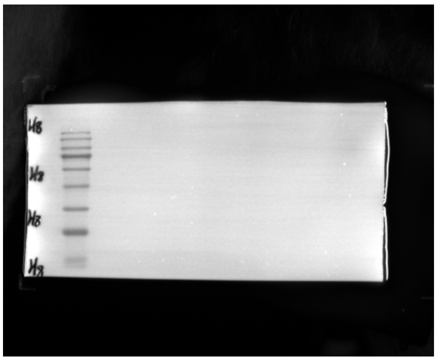	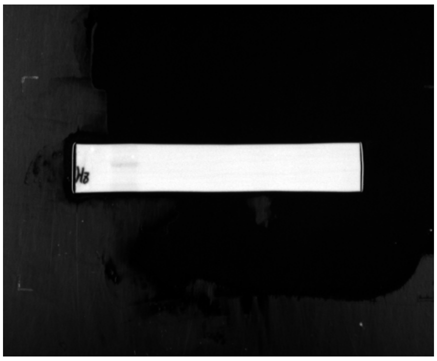
Full membrane image	White light image
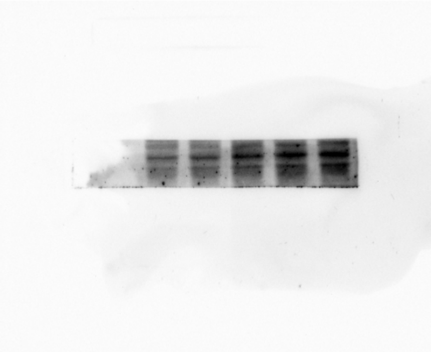	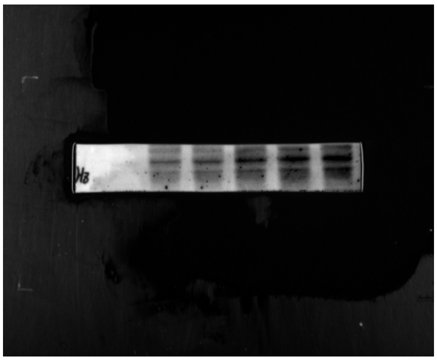

Chemiluminescent image	Composite image

Figure1D-BV2-BDNF

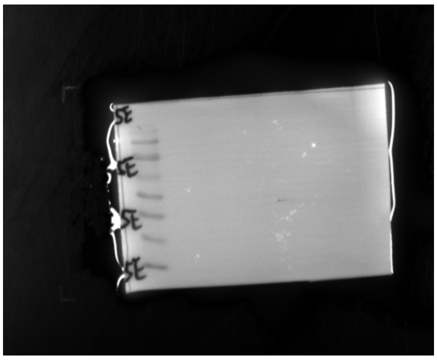<br>Full membrane image	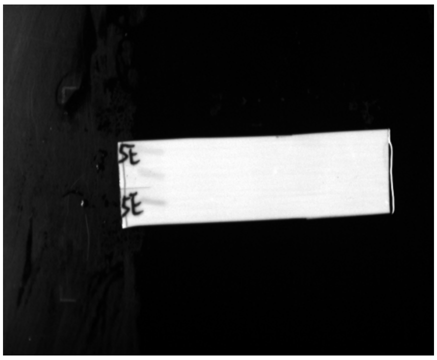<br>White light image
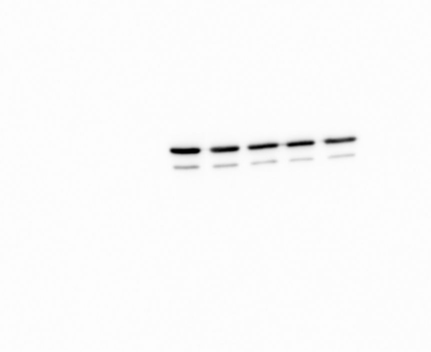<br>Chemiluminescent image	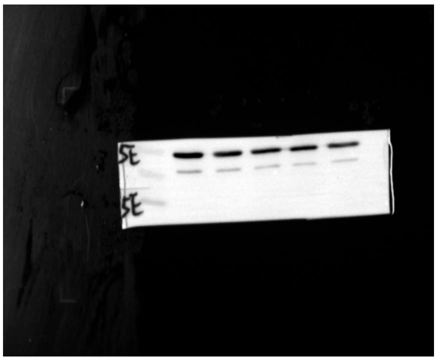<br>Composite image

Figure1D-BV2-β-actin

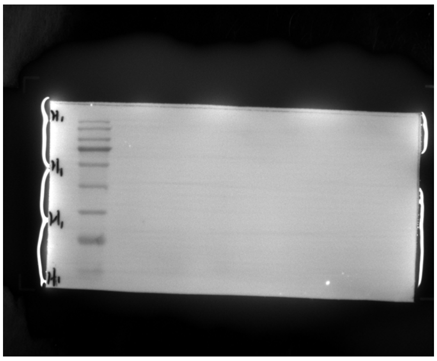<br>Full membrane image-同 Bax	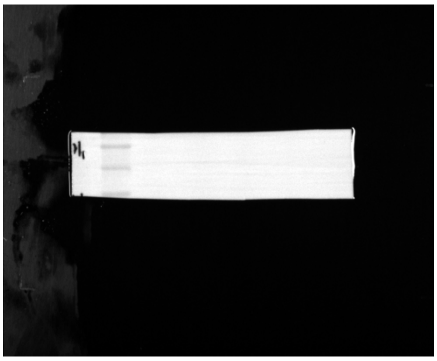<br>White light image
	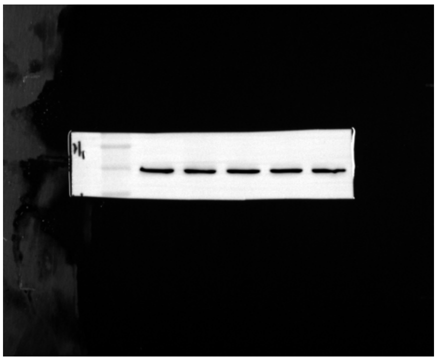

Chemiluminescent image	Composite image

Figure2A

Figure2A-CD63

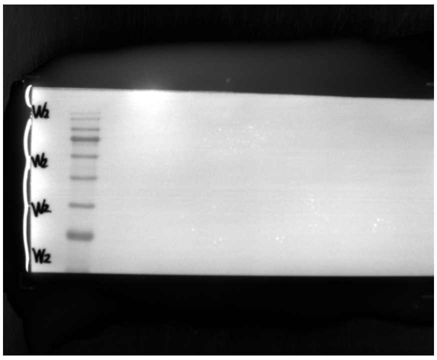<br>Full membrane image	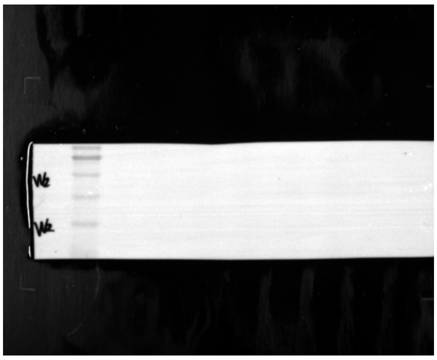<br>White light image
 Chemiluminescent image	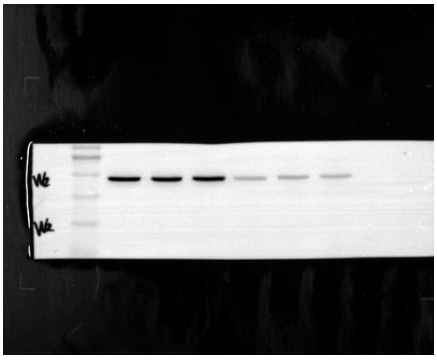<br>Composite image

Figure2A-TSG101

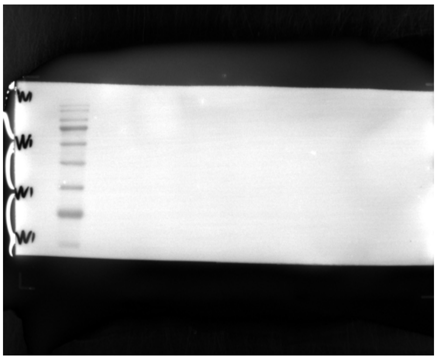<br>Full membrane image	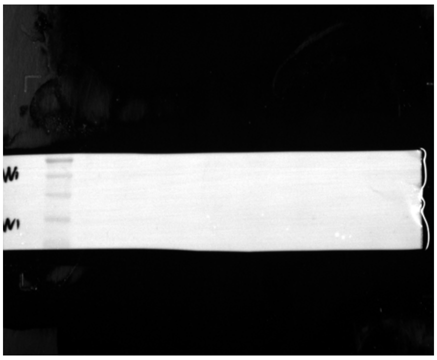<br>White light image

 Chemiluminescent image	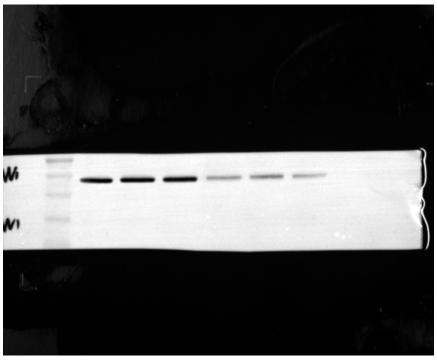<br>Composite image

Figure3F

Figure3F-HT22-Bax

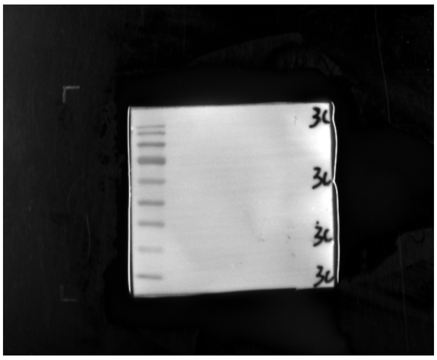<br>Full membrane image	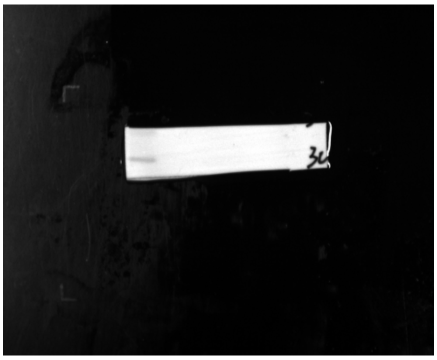<br>White light image
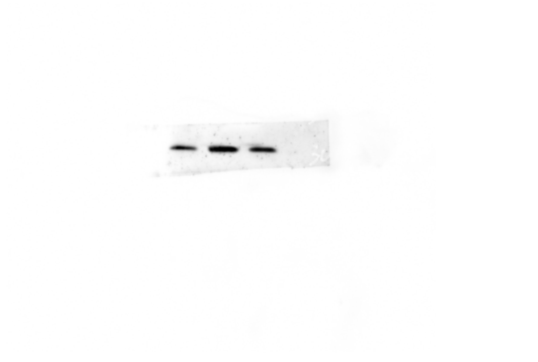<br>Chemiluminescent image	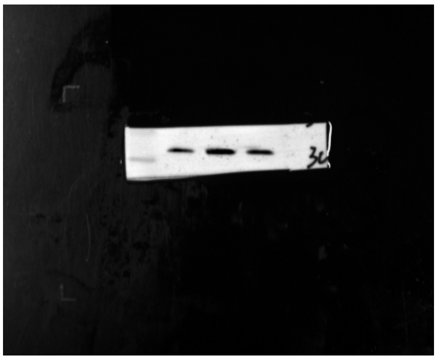<br>Composite image

Figure3F-HT22-Bcl2

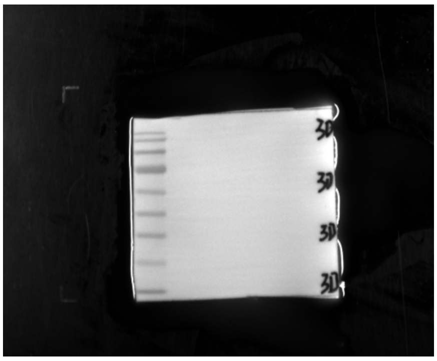	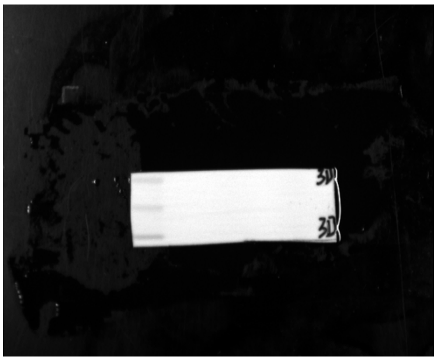

Full membrane image	White light image
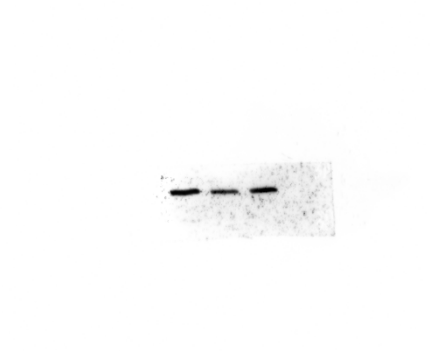 Chemiluminescent image	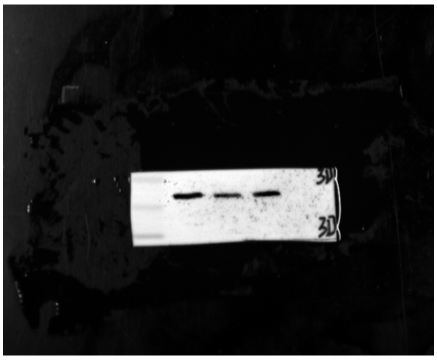 Composite image

Figure3F-HT22-Caspase3

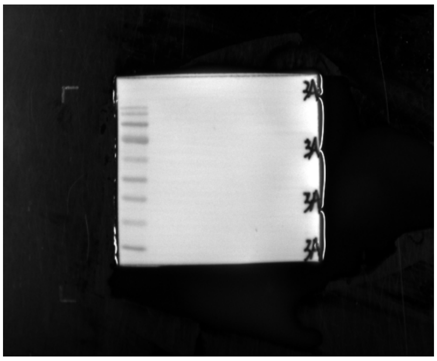 Full membrane image	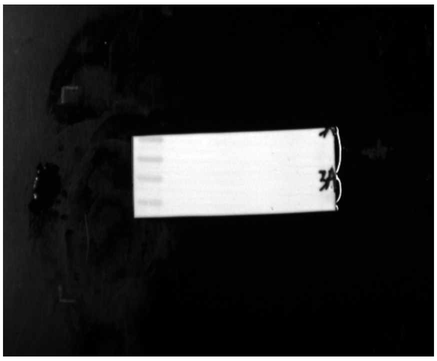 White light image
Chemiluminescent image	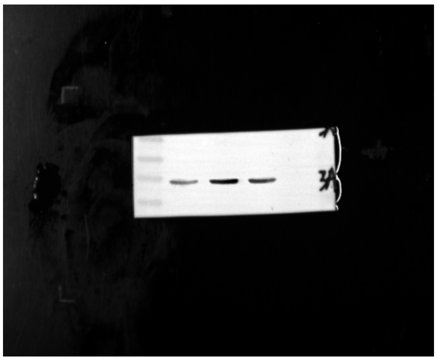 Composite image

Figure3F-HT22-BDNF

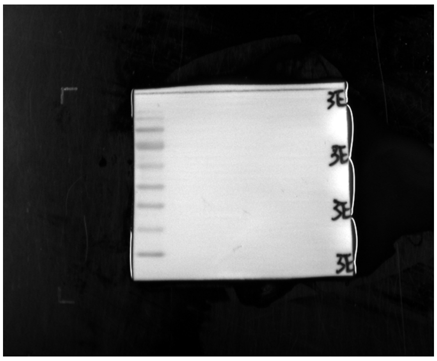	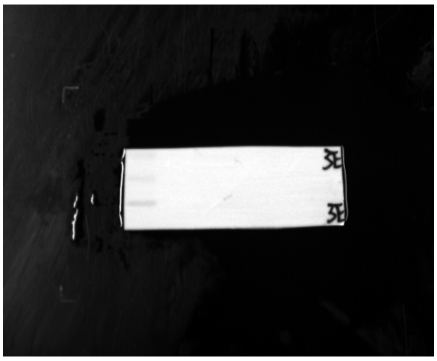

Full membrane image	White light image
 Chemiluminescent image	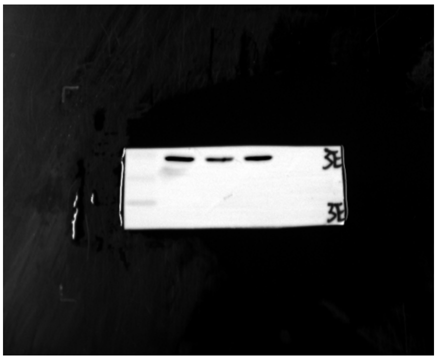<br>Composite image

Figure3F-HT22-β-actin

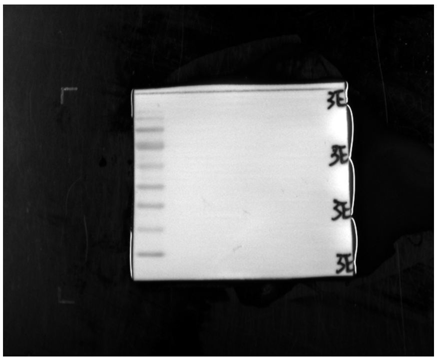<br>Full membrane image-同 BDNF	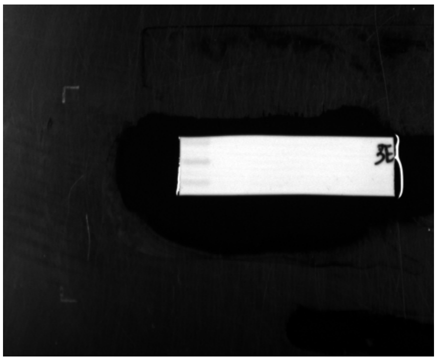<br>White light image
 Chemiluminescent image	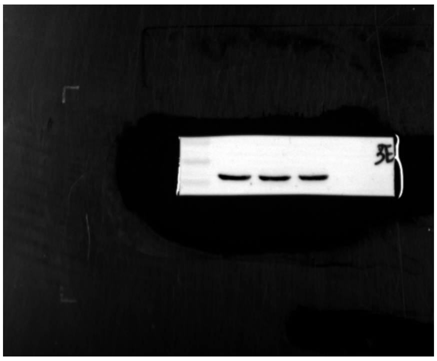<br>Composite image

Figure3F-BV2-Bax

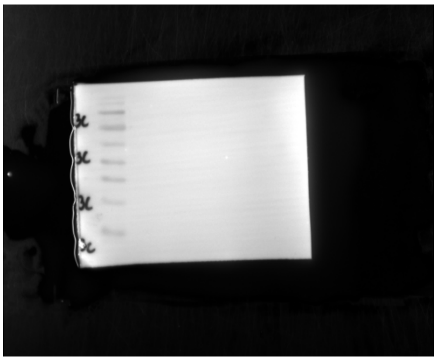	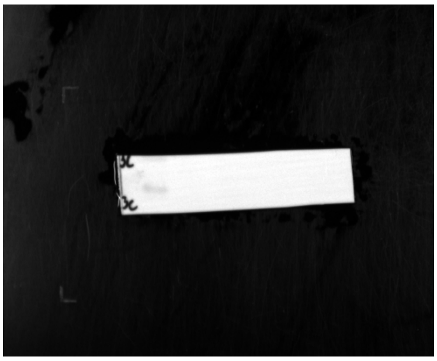

Full membrane image	White light image
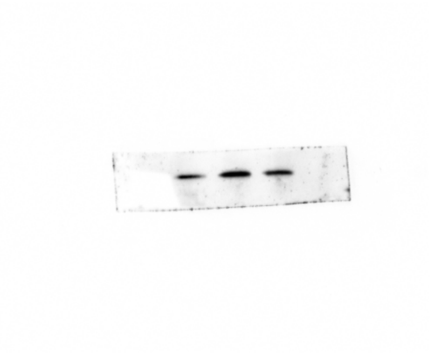 Chemiluminescent image	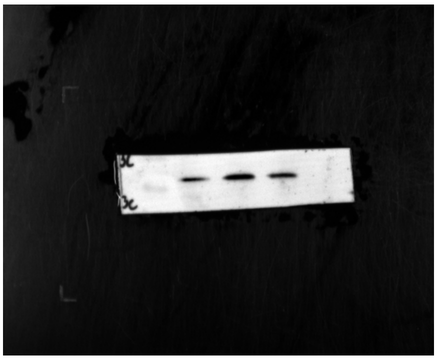 Composite image

Figure3F-BV2-Bcl2

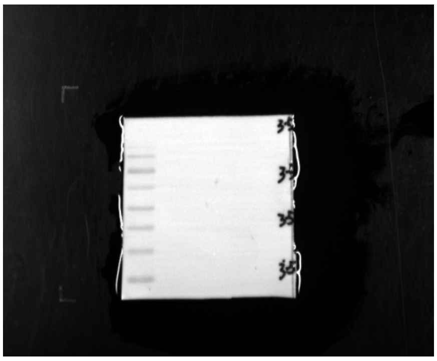 Full membrane image	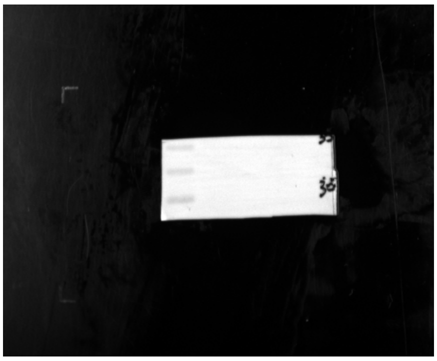 White light image
Chemiluminescent image	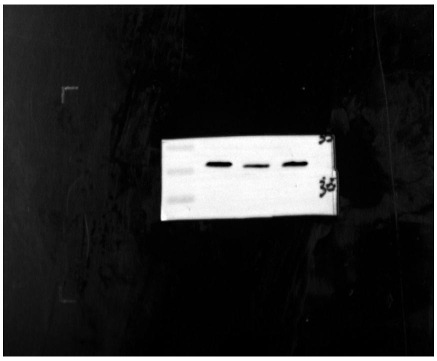 Composite image

Figure3F-BV2-Caspase3

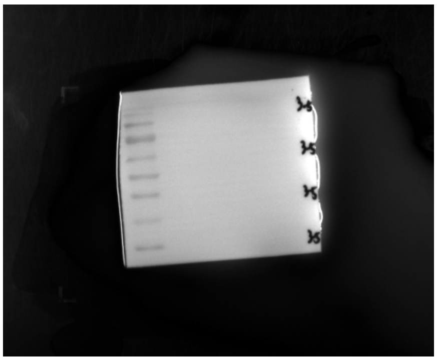	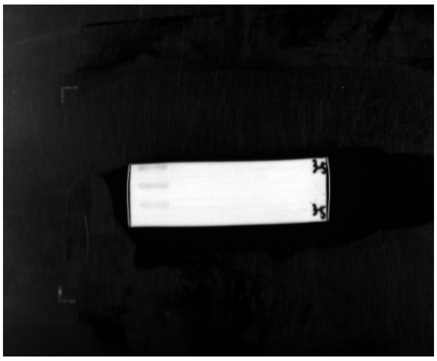

Full membrane image	White light image
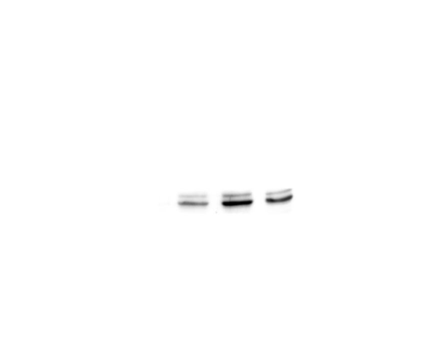<br>Chemiluminescent image	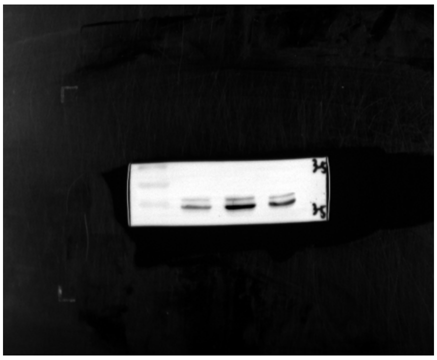<br>Composite image

Figure3F-BV2-BDNF

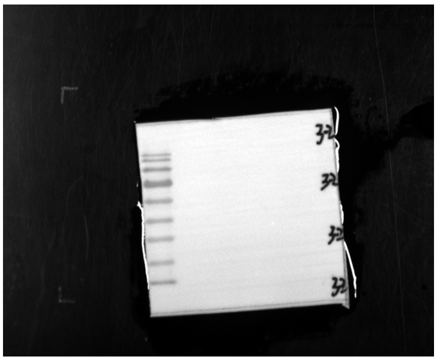<br>Full membrane image	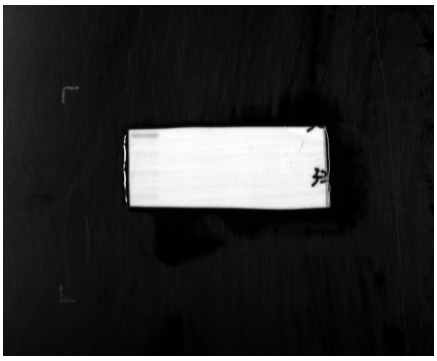<br>White light image
 Chemiluminescent image	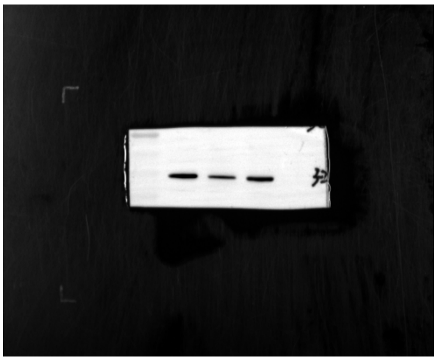<br>Composite image

Figure3F-BV2-β-actin Figure3F-BV2-BDNF

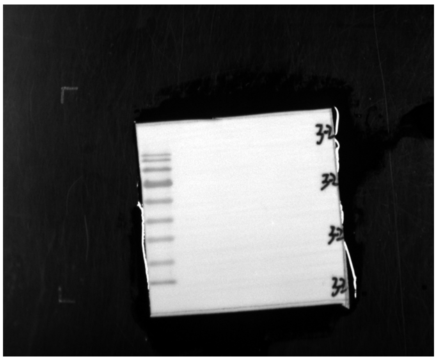	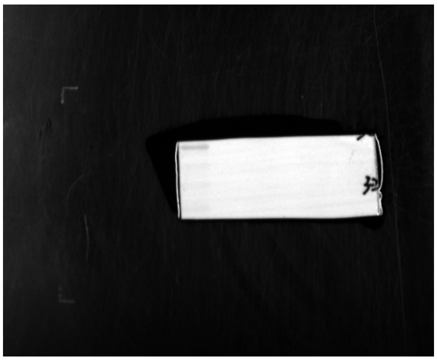

Full membrane image	White light image
Chemiluminescent image	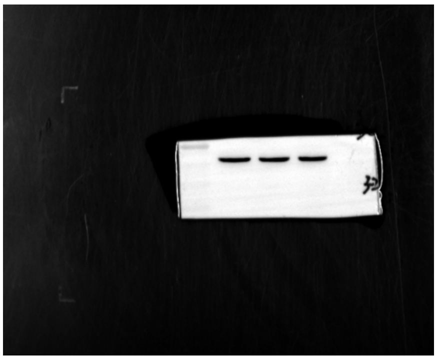 Composite image

Figure4E

Figure4E-Foxm1

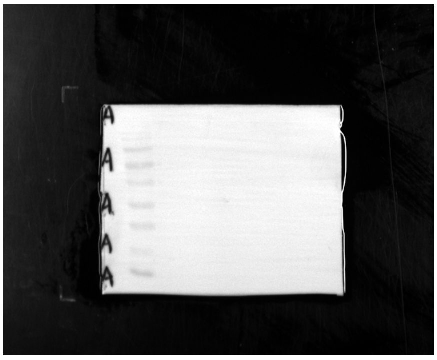 Full membrane image	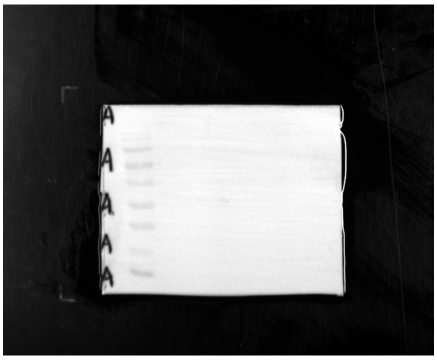 White light image
Chemiluminescent image	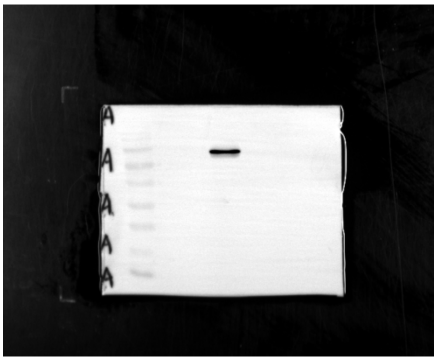 Composite image

Figure4F

Figure4F-BV2-Foxm1

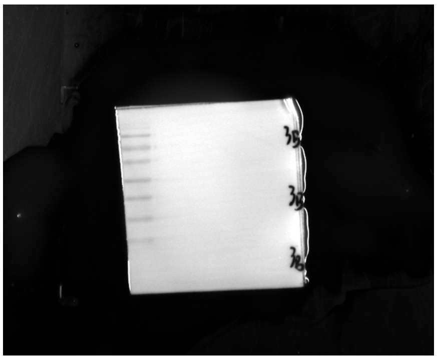<br>Full membrane image	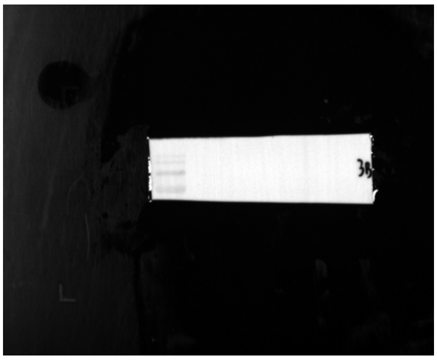<br>White light image
 Chemiluminescent image	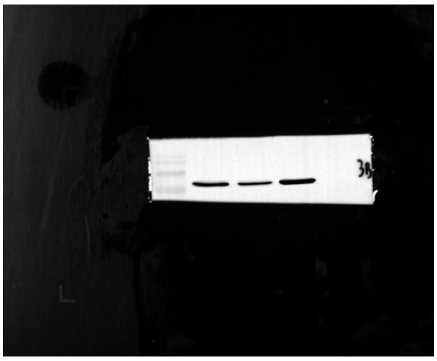<br>Composite image

Figure4F-BV2-β-actin

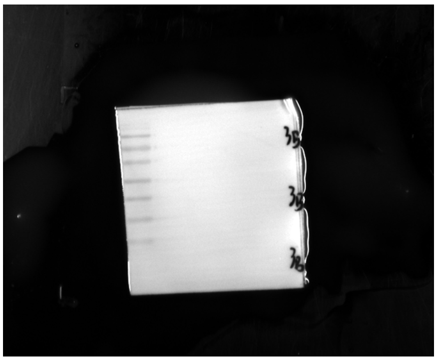<br>Full membrane image	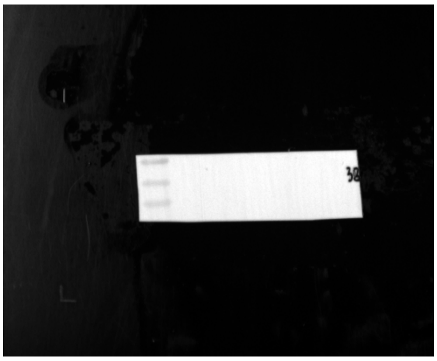<br>White light image
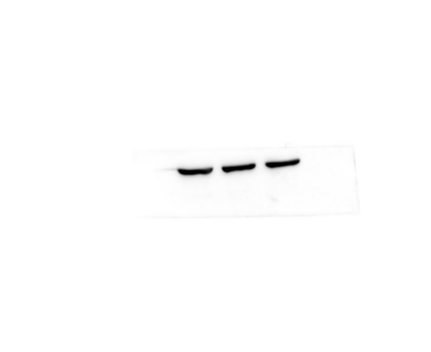<br>Chemiluminescent image	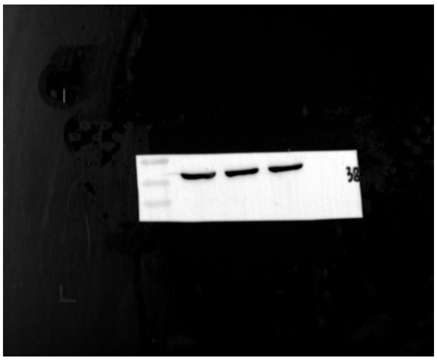<br>Composite image

Figure4F-HT22-Foxm1

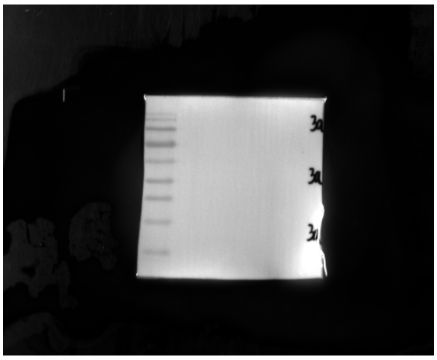<br>Full membrane image	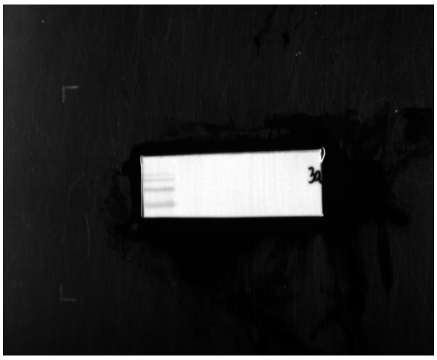<br>White light image
 Chemiluminescent image	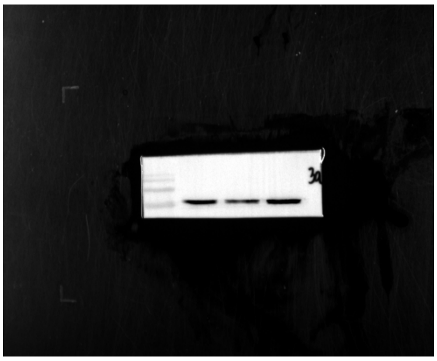<br>Composite image

Figure4F-HT22-β-actin

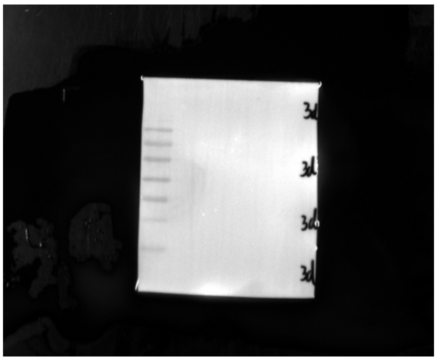<br>Full membrane image	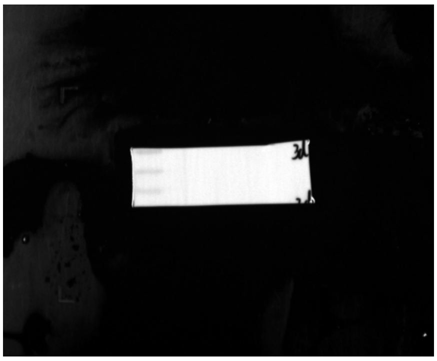<br>White light image
 Chemiluminescent image	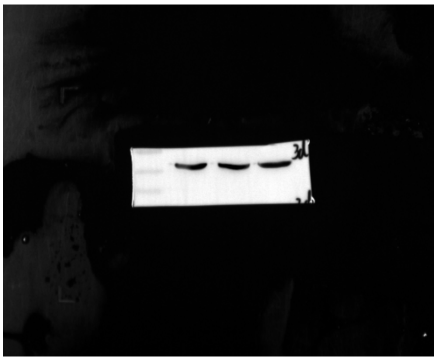<br>Composite image

Figure5A

Figure5A-Foxm1

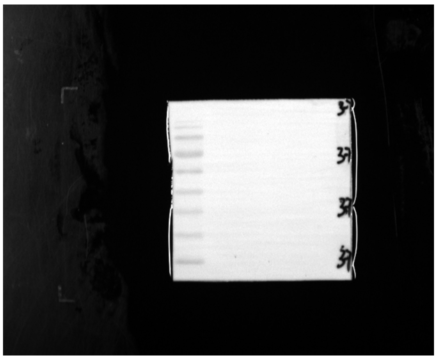<br>Full membrane image	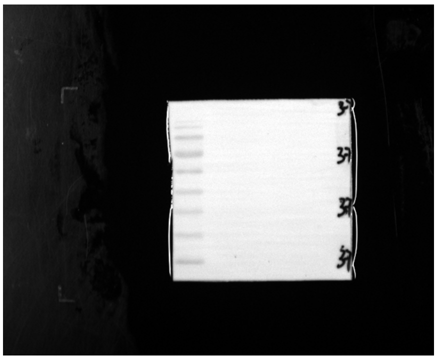<br>White light image
 Chemiluminescent image	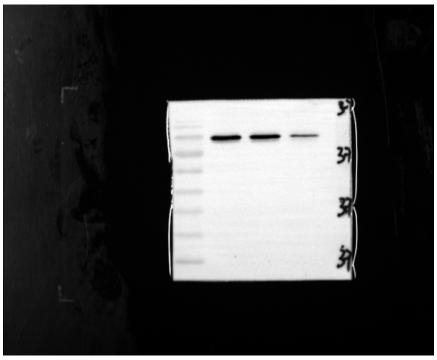<br>Composite image

Figure5A-β-actin

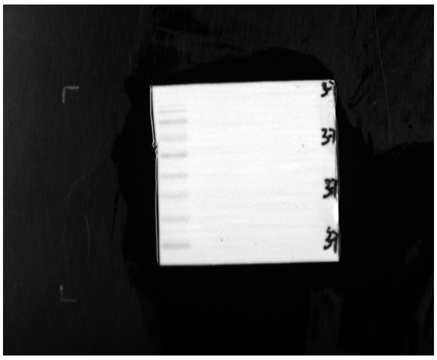<br>Full membrane image-同 Figure5A-Foxm1	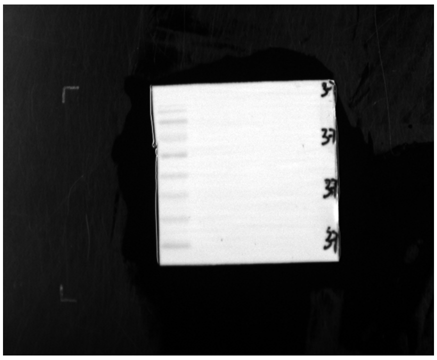<br>White light image
 Chemiluminescent image	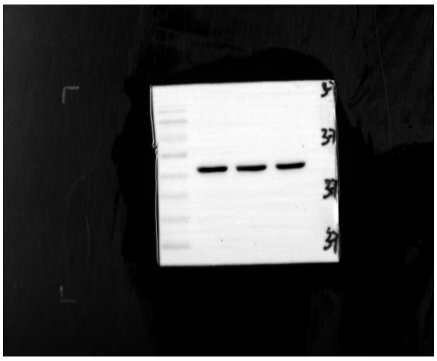<br>Composite image

Figure5B

Figure5B-Foxm1

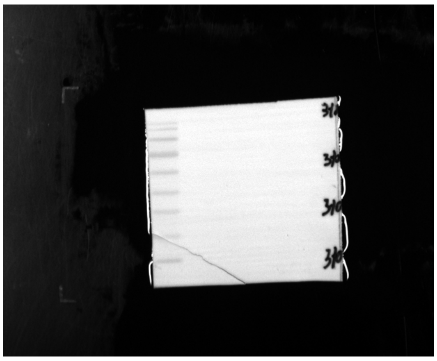<br>Full membrane image	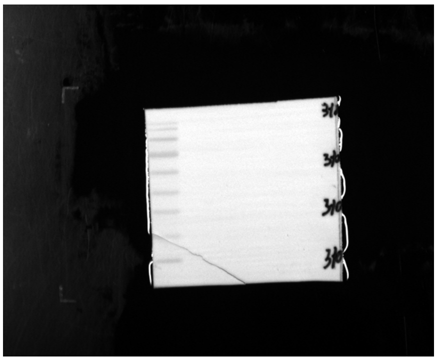<br>White light image
 Chemiluminescent image	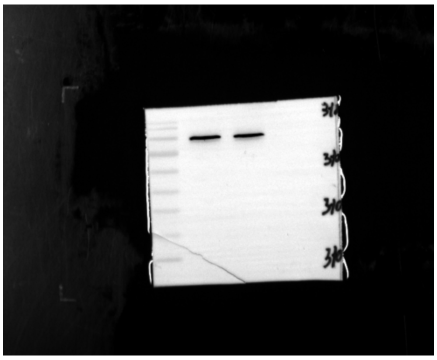<br>Composite image

Figure5C

Figure5C-HT22-Foxm1

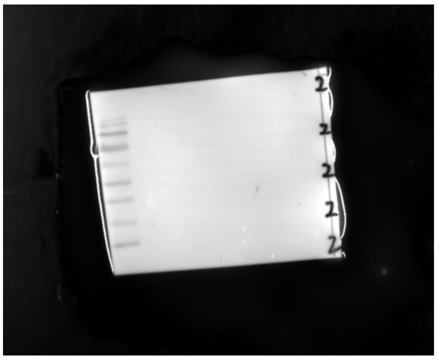<br>Full membrane image	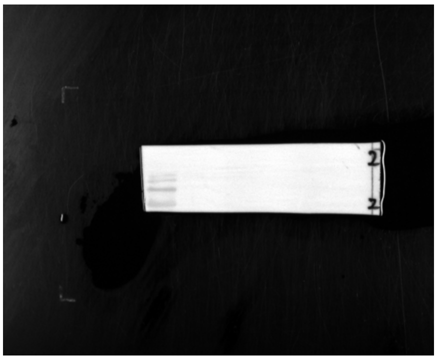<br>White light image
	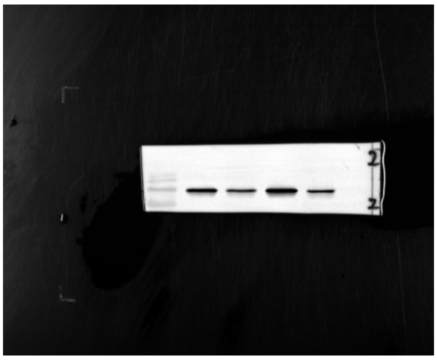

Chemiluminescent image	Composite image

Figure5C-HT22-β-actin

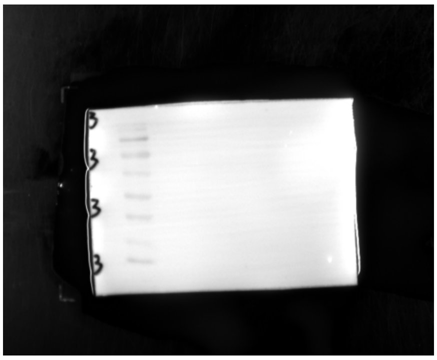<br>Full membrane image	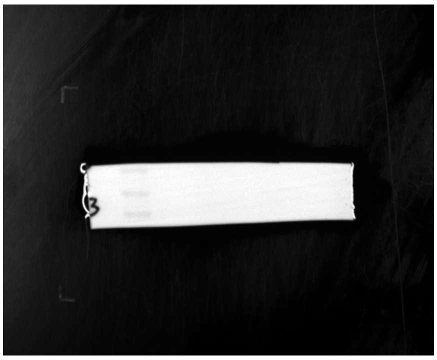<br>White light image
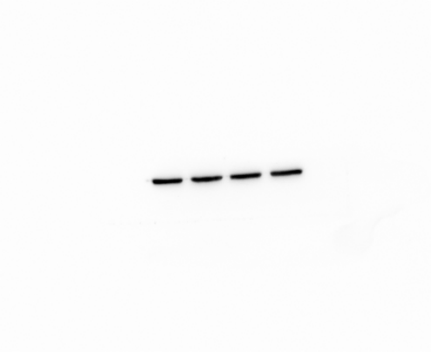<br>Chemiluminescent image	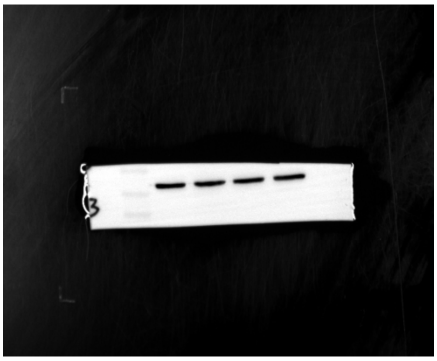<br>Composite image

Figure5D

Figure5D-BV2-Foxm1

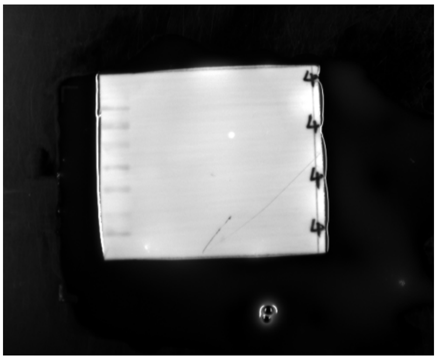<br>Full membrane image	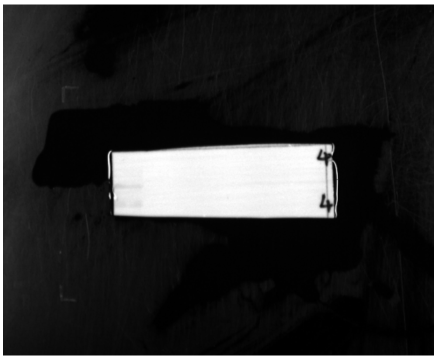<br>White light image

 Chemiluminescent image	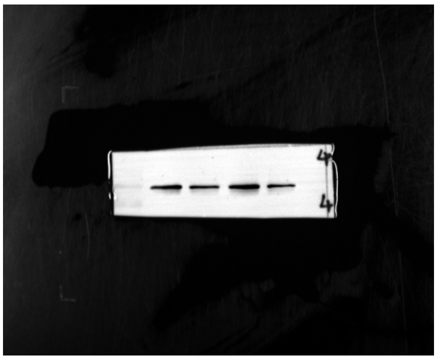<br>Composite image

Figure5D-BV2-β-actin

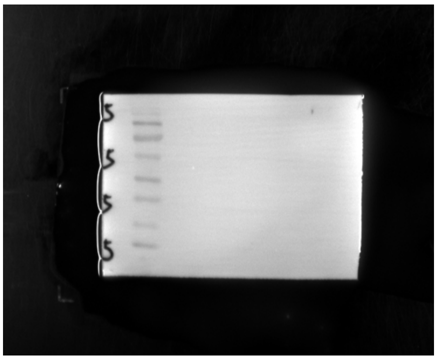<br>Full membrane image	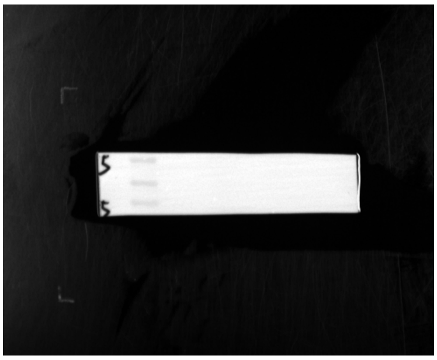<br>White light image
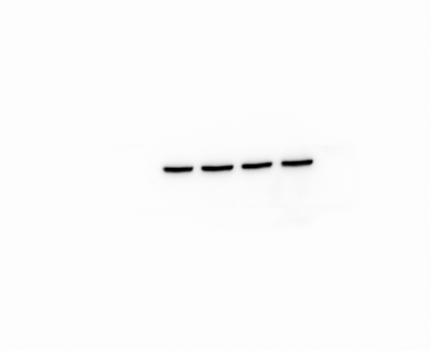<br>Chemiluminescent image	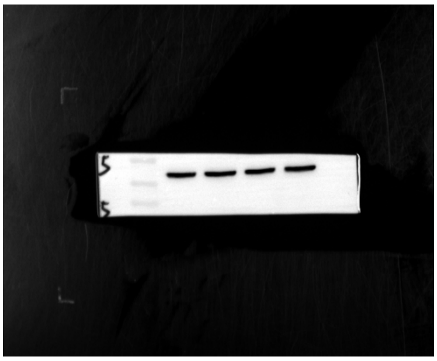<br>Composite image

Figure5I

Figure5I-HT22-Bax

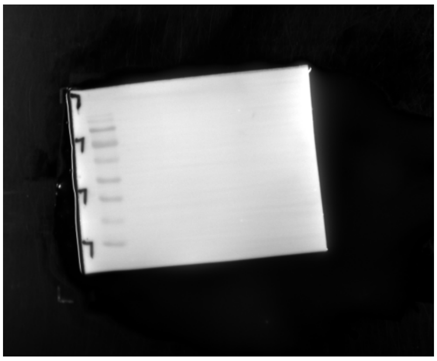	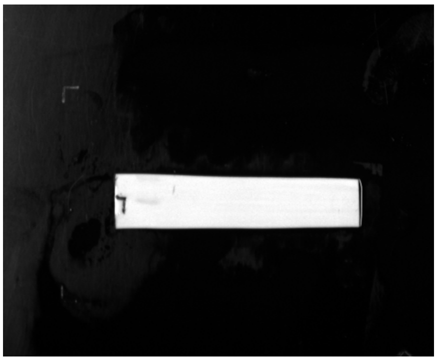

Full membrane image	White light image
 Chemiluminescent image	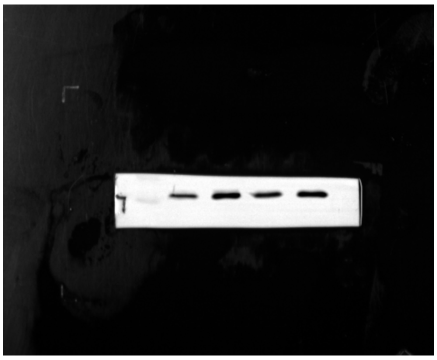<br>Composite image

Figure5I-HT22-Bcl2

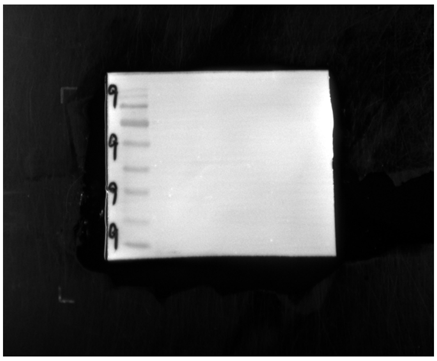<br>Full membrane image	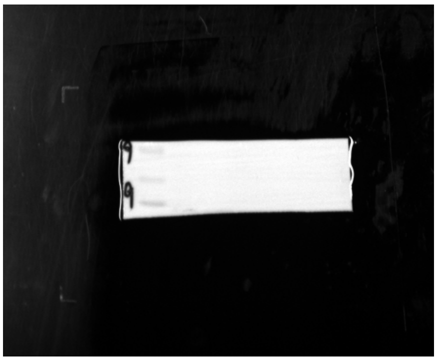<br>White light image
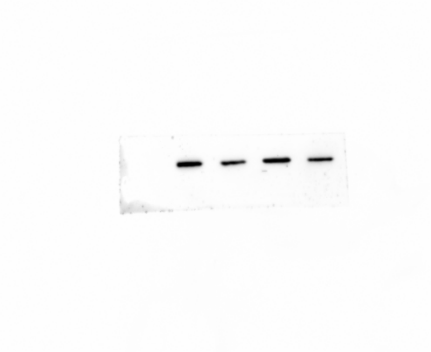<br>Chemiluminescent image	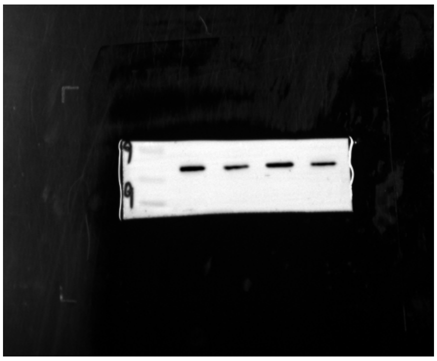<br>Composite image

Figure5I-HT22-Caspase3

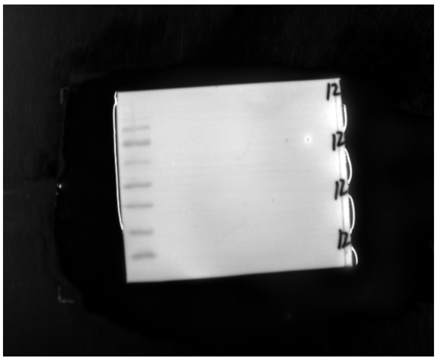	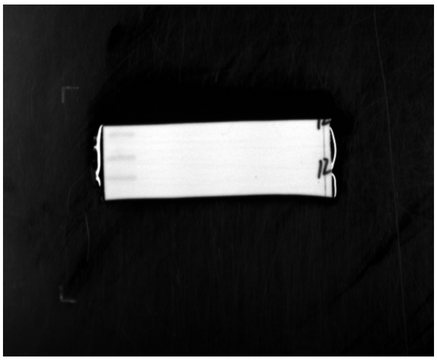

Full membrane image	White light image
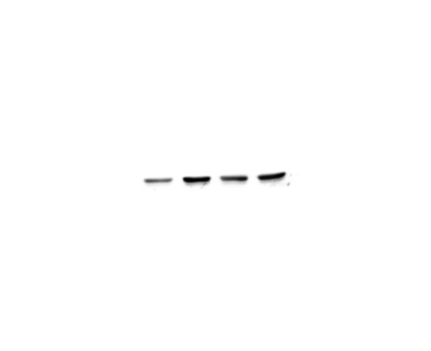<br>Chemiluminescent image	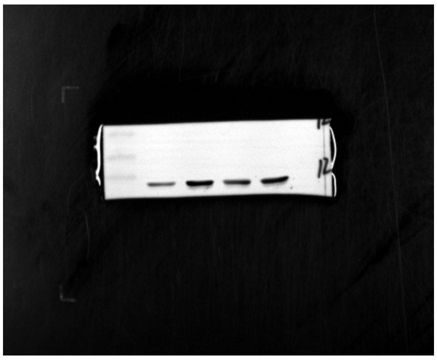<br>Composite image

Figure5I-HT22-BDNF

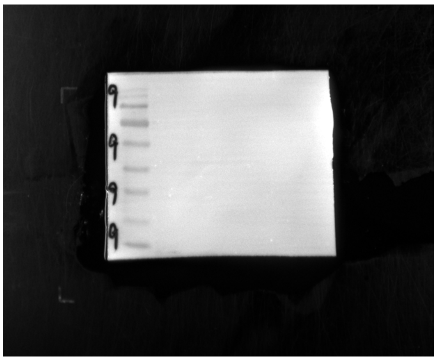<br>Full membrane image	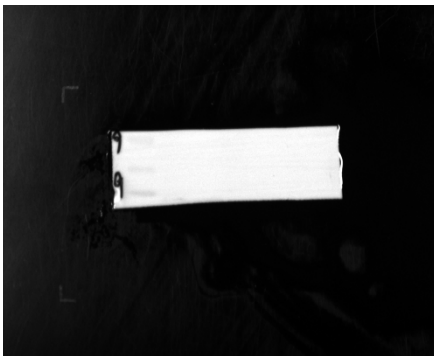<br>White light image
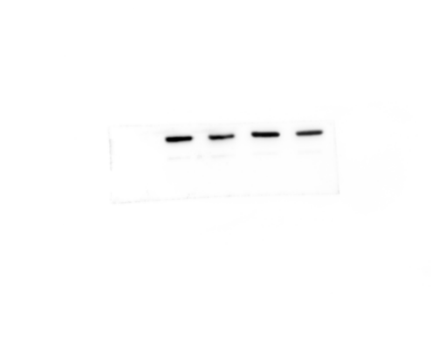<br>Chemiluminescent image	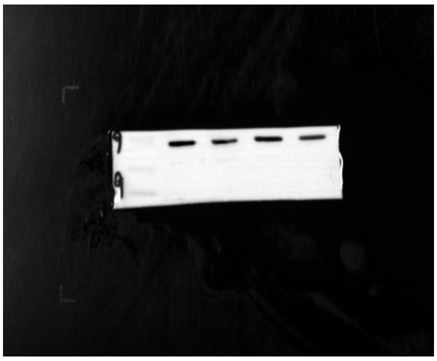<br>Composite image

Figure5I-HT22-β-actin

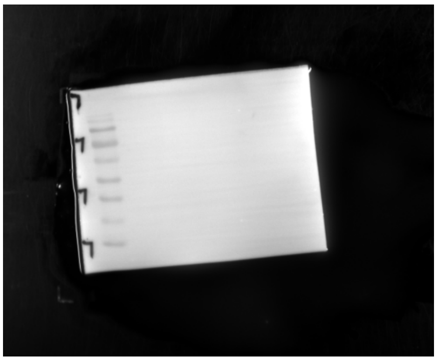	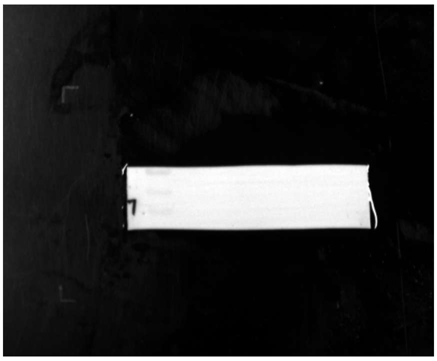

Full membrane image-同 Figure5I-HT22-Bax	White light image
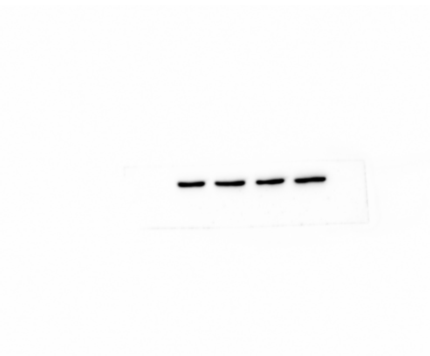<br>Chemiluminescent image	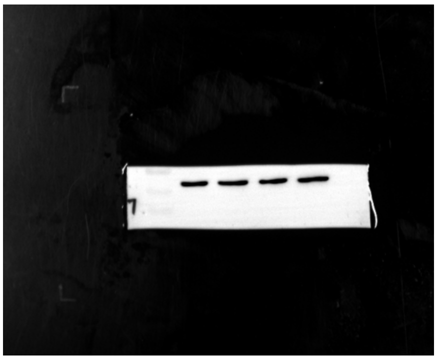<br>Composite image

Figure5J

Figure5J-BV2-Bax

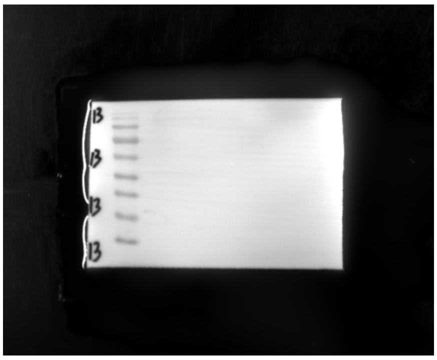<br>Full membrane image	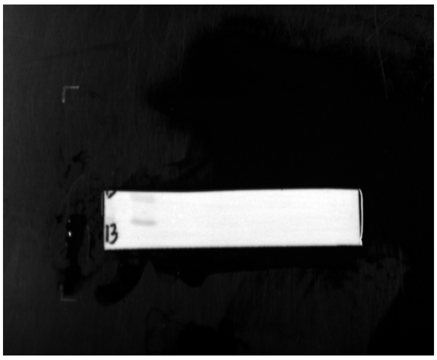<br>White light image
 Chemiluminescent image	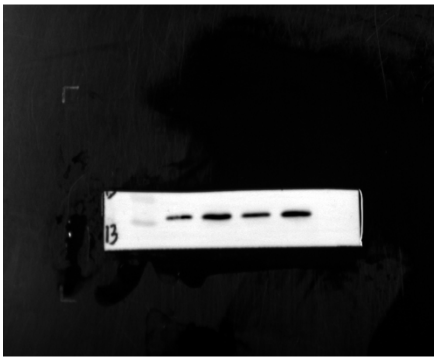<br>Composite image

Figure5J-BV2-Bcl2

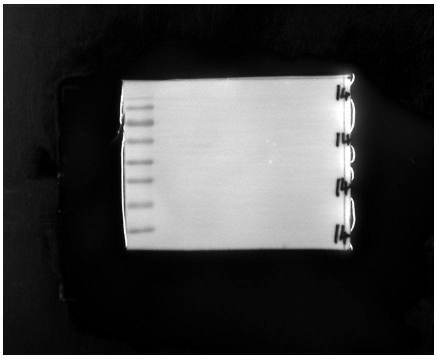<br>Full membrane image	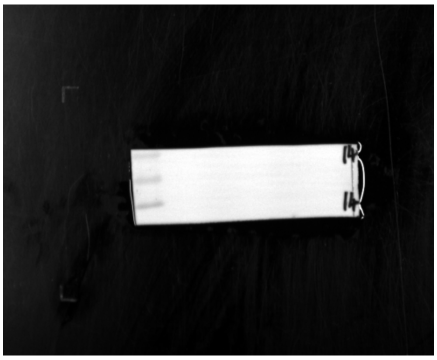<br>White light image
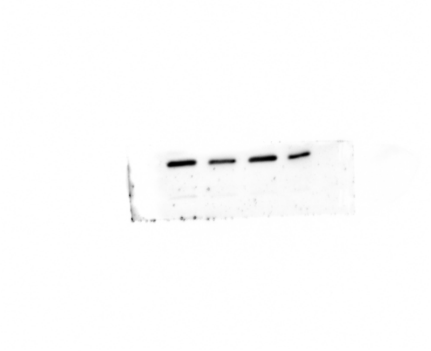<br>Chemiluminescent image	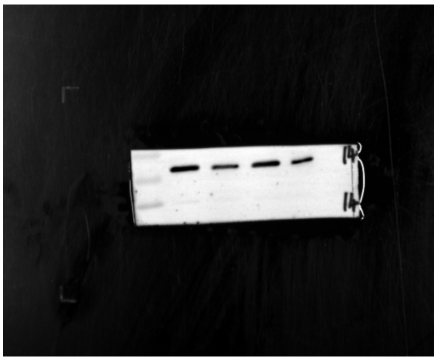<br>Composite image

Figure5J-BV2-Caspase3

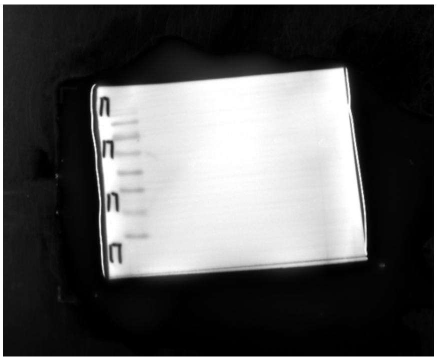<br>Full membrane image	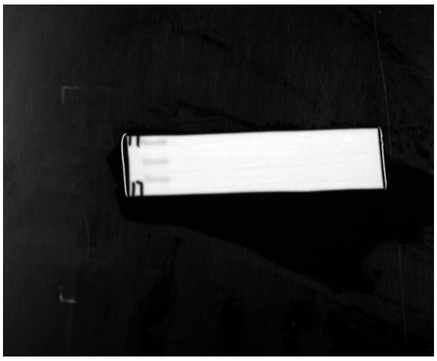<br>White light image
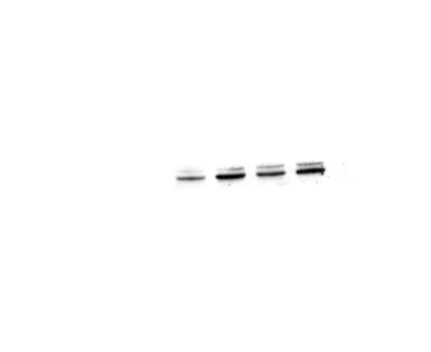<br>Chemiluminescent image	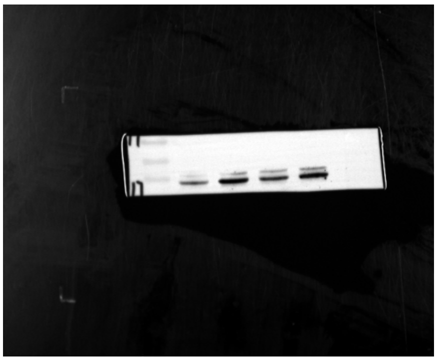<br>Composite image

Figure5J-BV2-BDNF

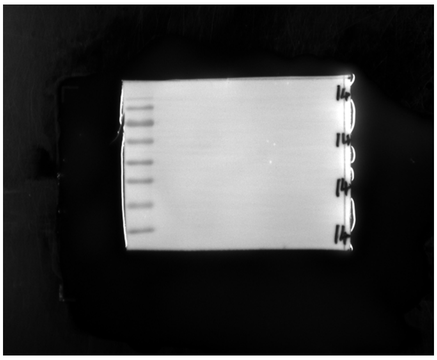<br>Full membrane image-同 Figure5J-BV2-Bcl2	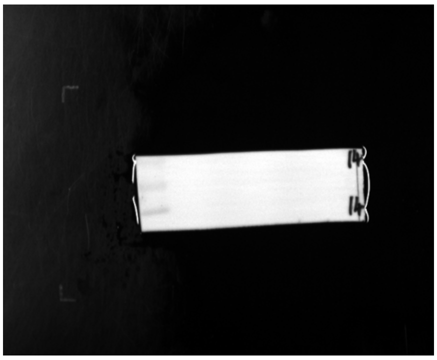<br>White light image
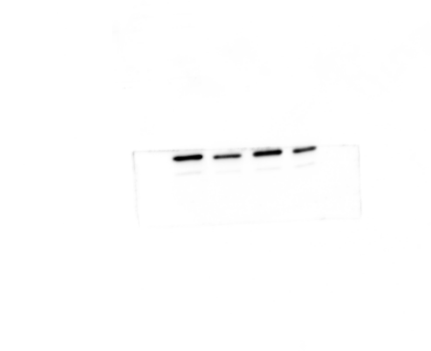<br>Chemiluminescent image	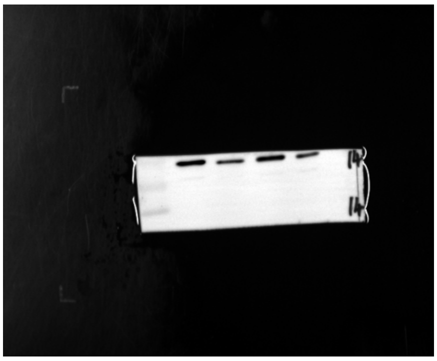<br>Composite image

Figure5J-BV2-β-actin

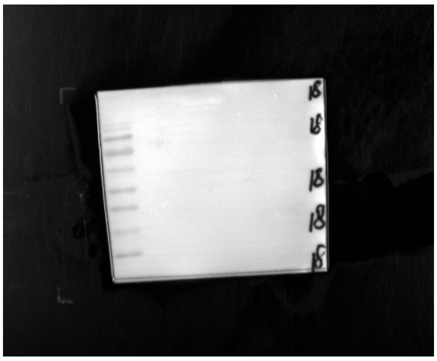<br>Full membrane image	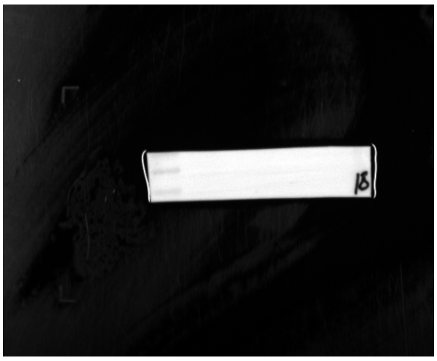<br>White light image
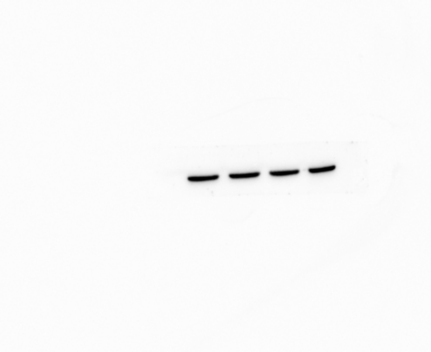<br>Chemiluminescent image	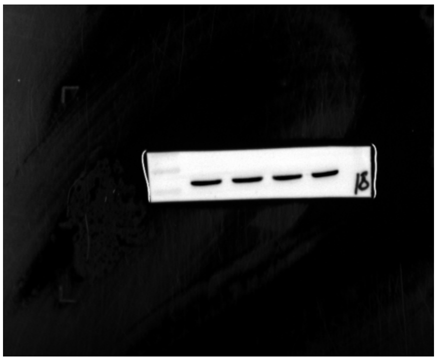<br>Composite image

Second replicate

Figure1D

Figure1D-HT22-Bax

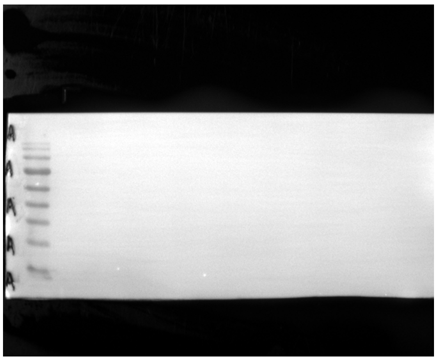<br>Full membrane image	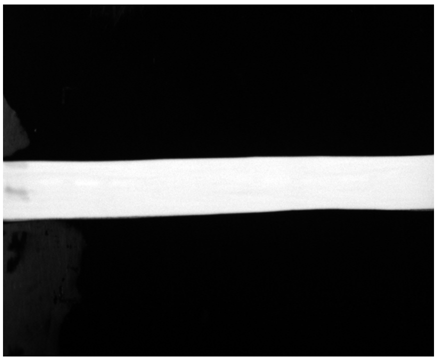<br>White light image
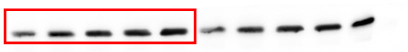<br>Chemiluminescent image	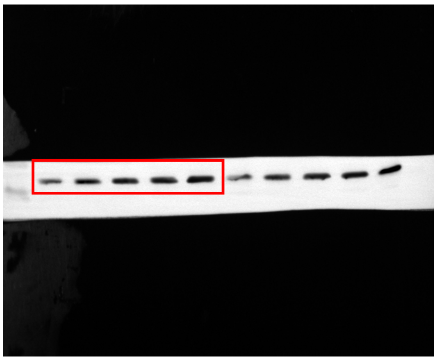<br>Composite image

Figure1D-HT22-Bcl2 Figure1D-HT22-Bax

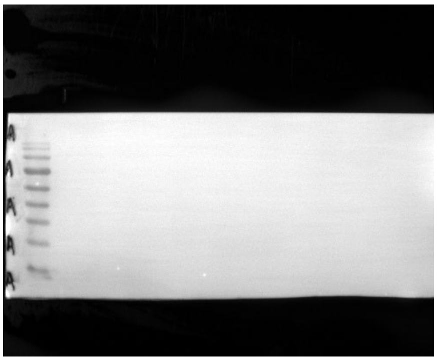<br>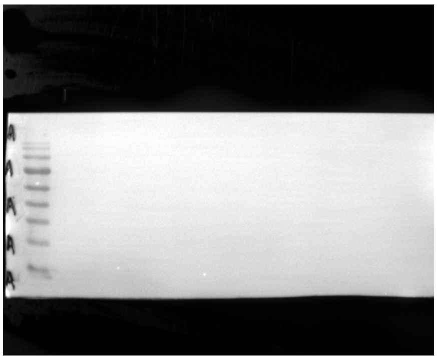<br>Full membrane image	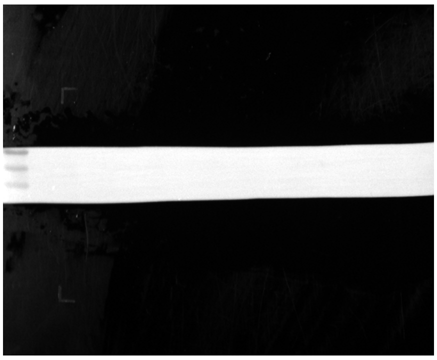<br>White light image

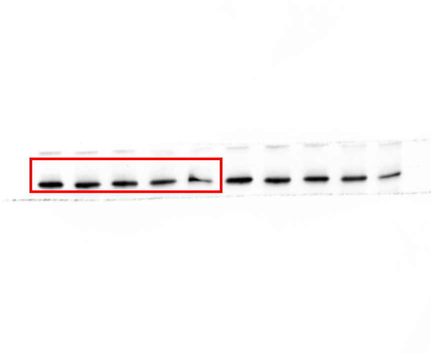

Chemiluminescent image

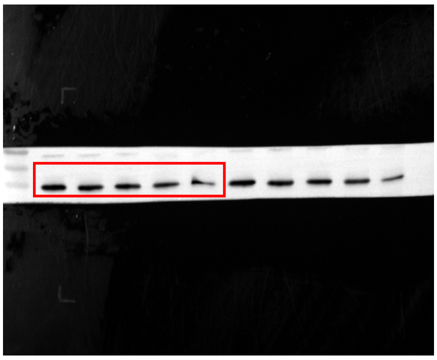

Composite image

Figure1D-HT22-Caspase3

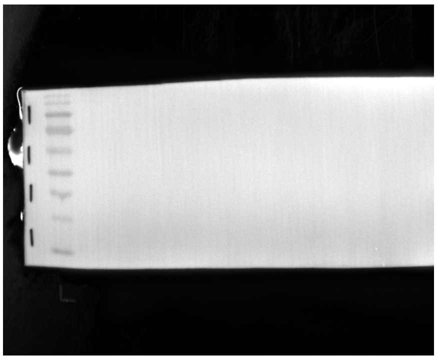

Full membrane image

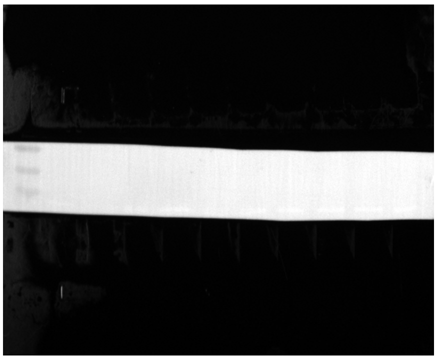

White light image

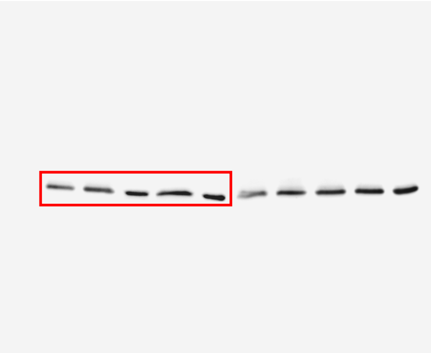

Chemiluminescent image

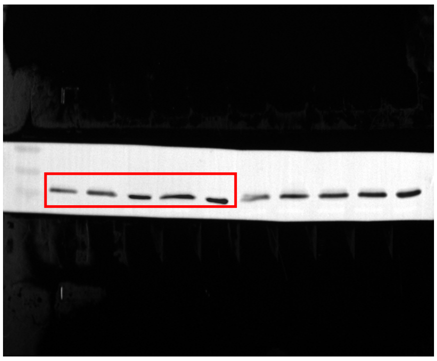

Composite image

Figure1D-HT22-BDNF

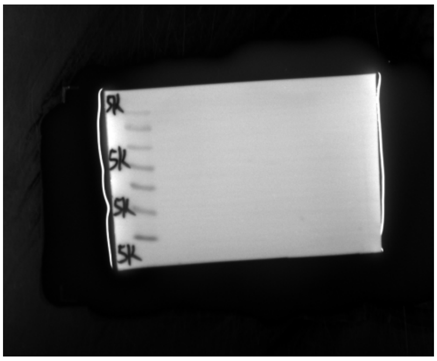

Full membrane image

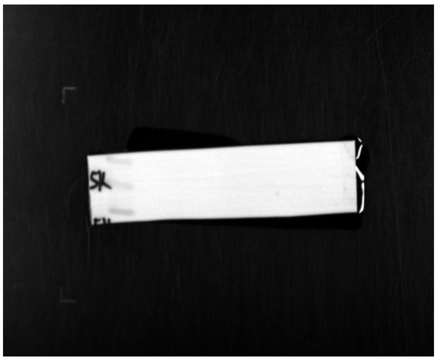

White light image

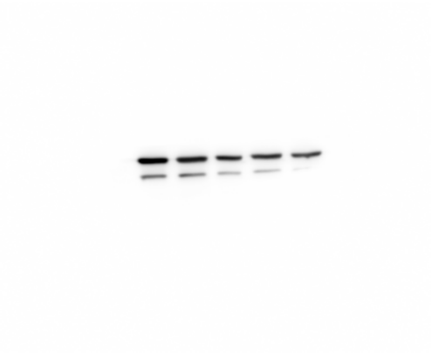<br>Chemiluminescent image	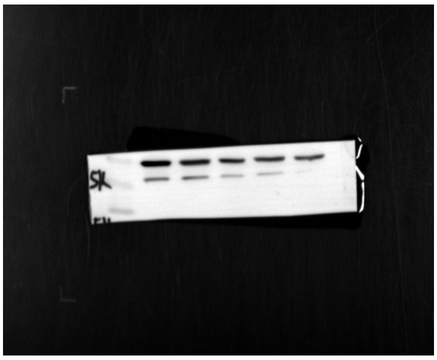<br>Composite image

Figure1D-HT22-β-actin Figure1D-HT22-Caspase3

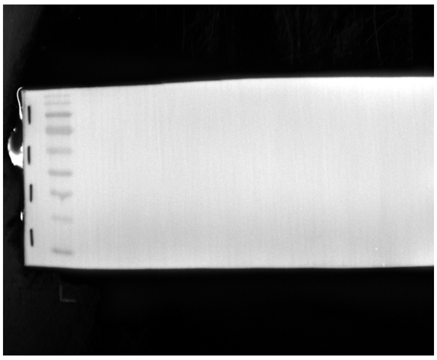<br>Full membrane image	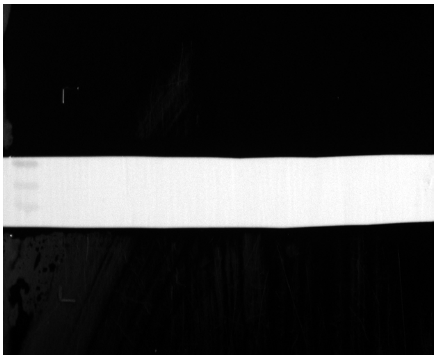<br>White light image
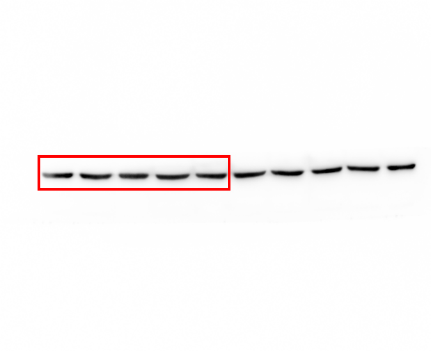<br>Chemiluminescent image	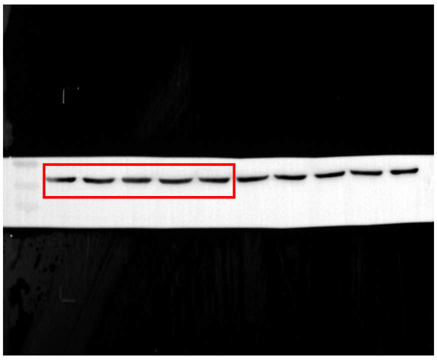<br>Composite image

Figure1D-BV2-Bax

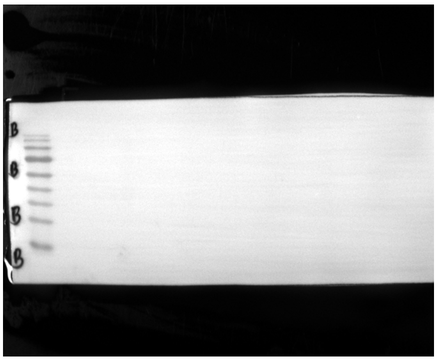	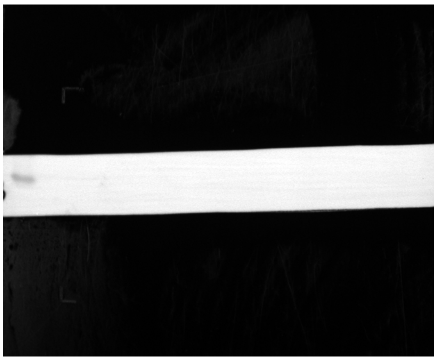

Full membrane image	White light image
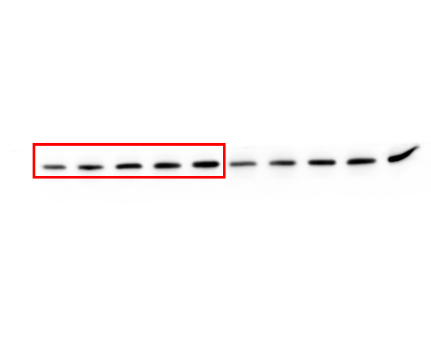<br>Chemiluminescent image	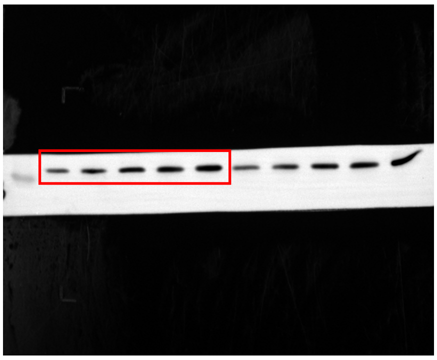<br>Composite image

Figure1D-BV2-Bcl2 Figure1D-BV2-Bax

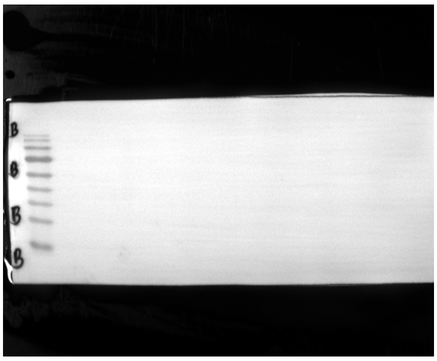<br>Full membrane image	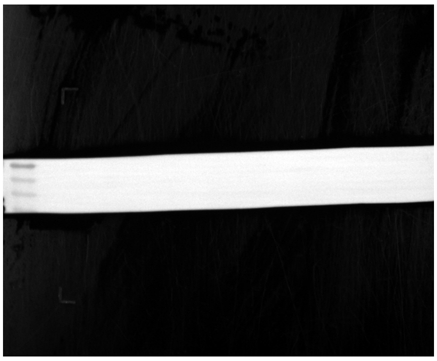<br>White light image
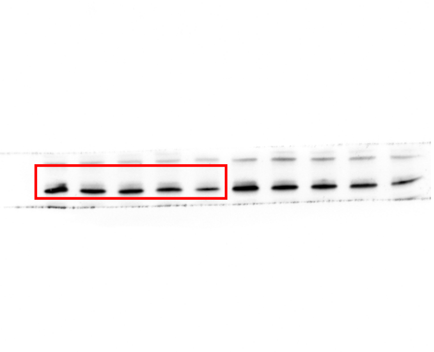<br>Chemiluminescent image	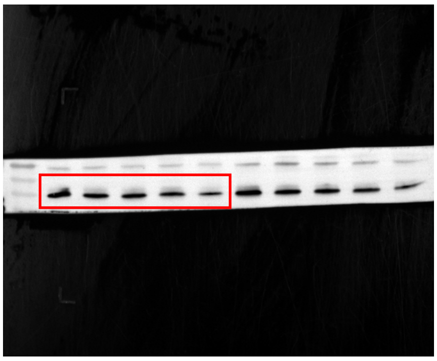<br>Composite image

Figure1D-BV2-Caspase3

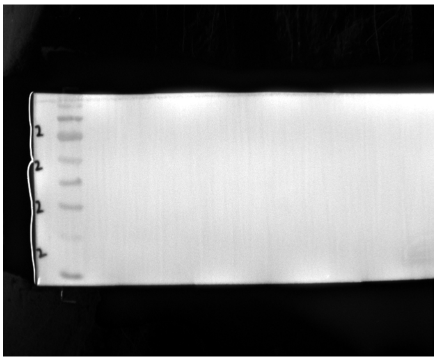	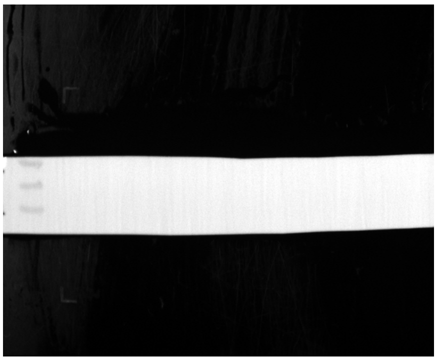

Full membrane image	White light image
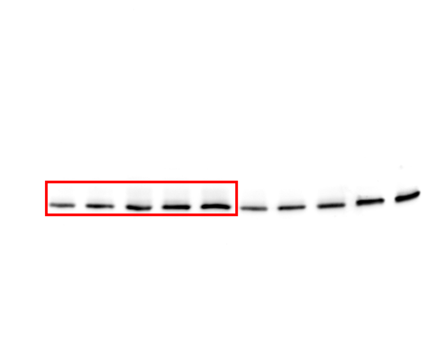 Chemiluminescent image	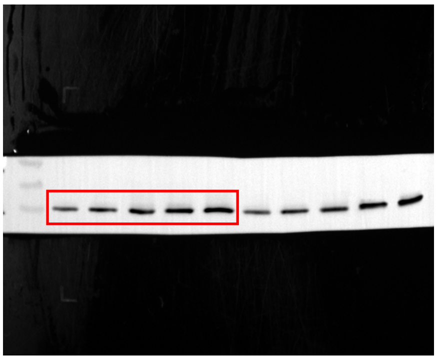 Composite image

Figure1D-BV2-BDNF

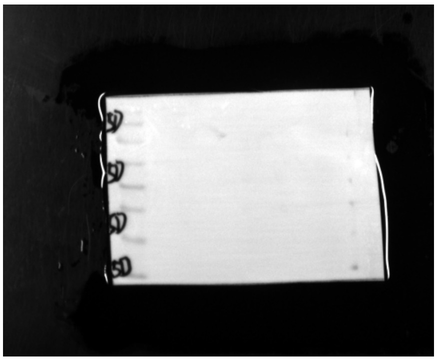 Full membrane image	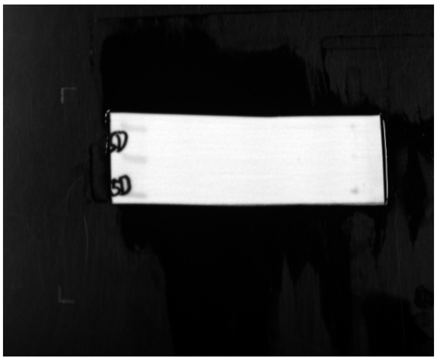 White light image
Chemiluminescent image	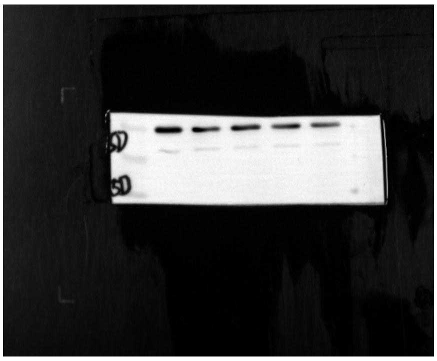 Composite image

Figure1D-BV2-β-actin Figure1D-BV2-Caspase3

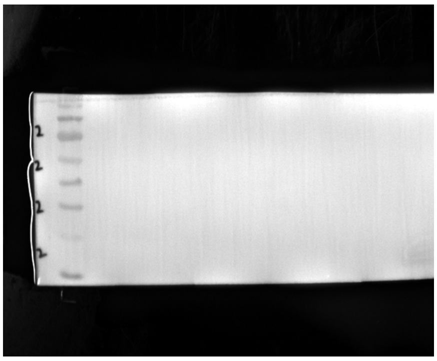	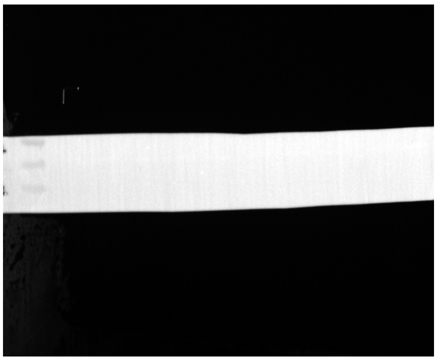

Full membrane image	White light image
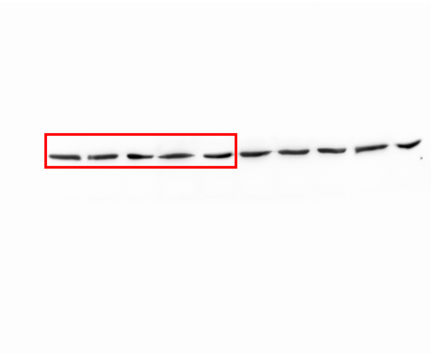<br>Chemiluminescent image	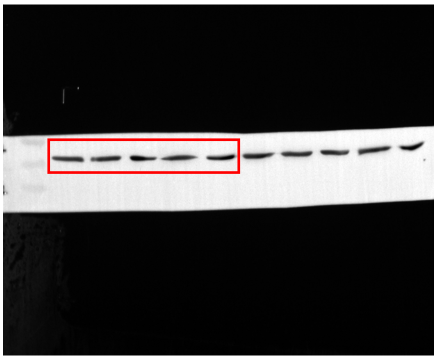<br>Composite image

Figure2A

Figure2A-CD63

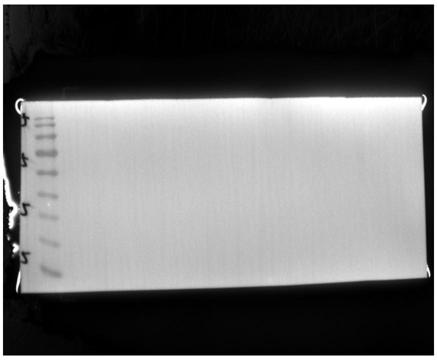<br>Full membrane image	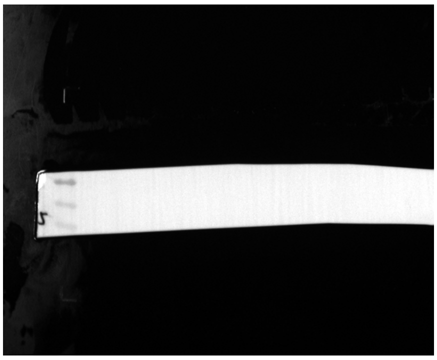<br>White light image
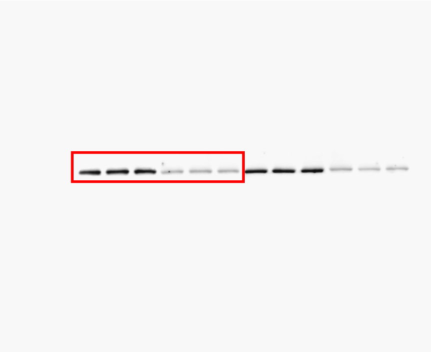<br>Chemiluminescent image	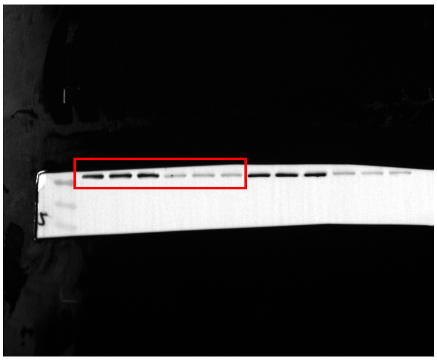<br>Composite image

Figure2A-TSG101

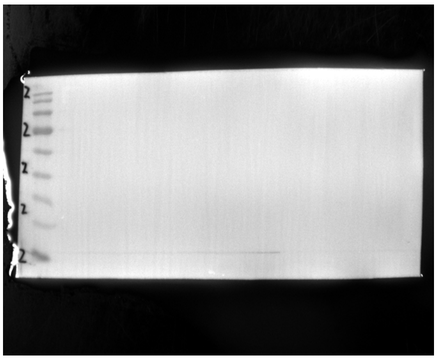 Full membrane image	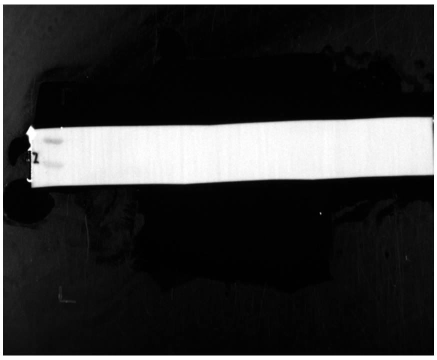 White light image
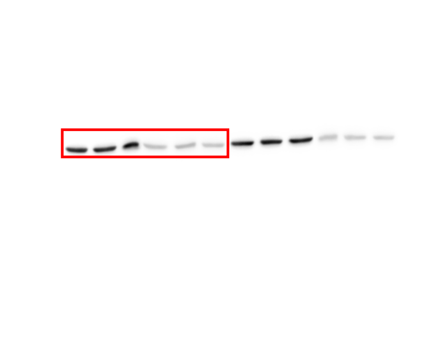 Chemiluminescent image	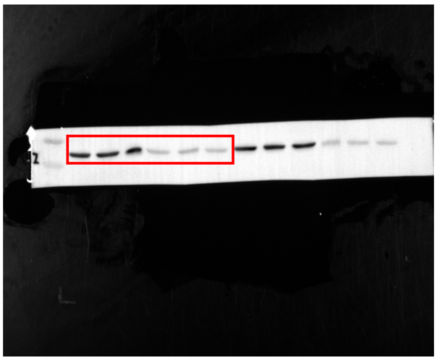 Composite image

Figure3F

Figure3F-HT22-Bax

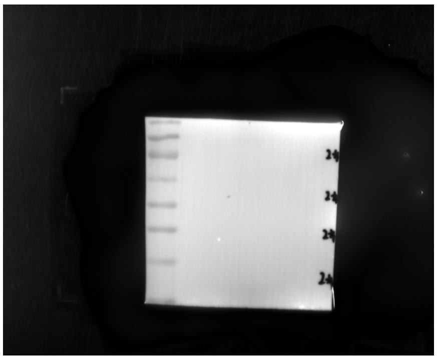 Full membrane image	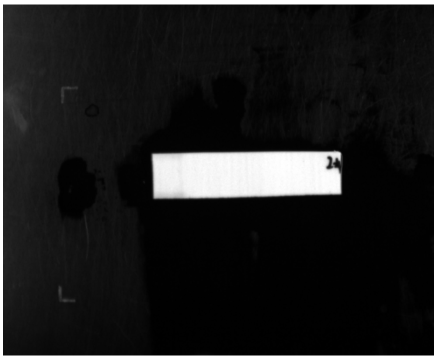 White light image
Chemiluminescent image	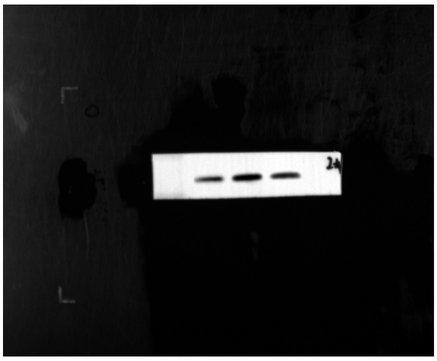 Composite image

Figure3F-HT22-Bcl2

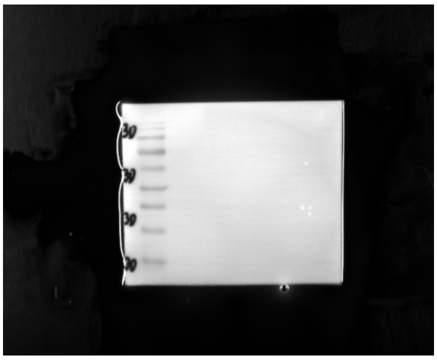

Full membrane image

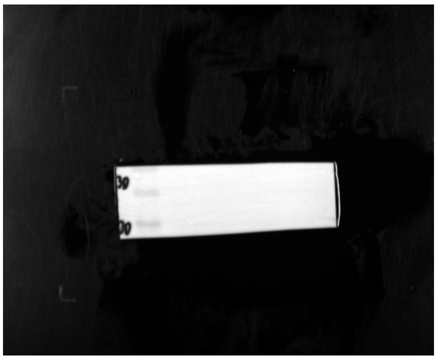

White light image

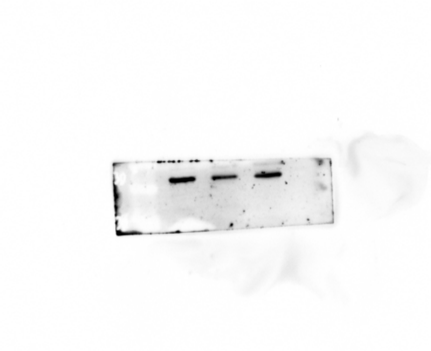

Chemiluminescent image

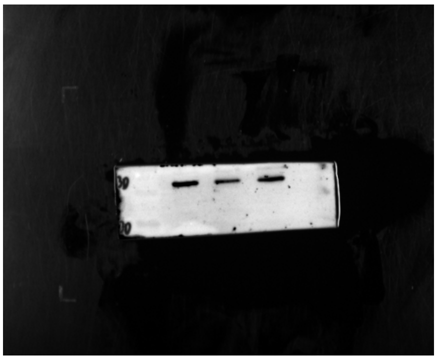

Composite image

Figure3F-HT22-Caspase3

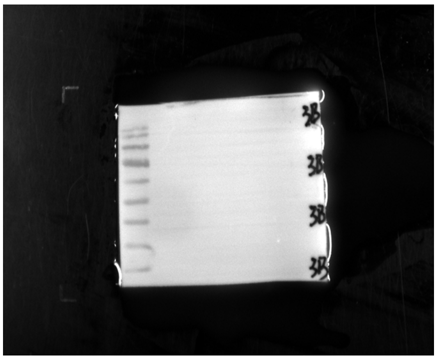

Full membrane image

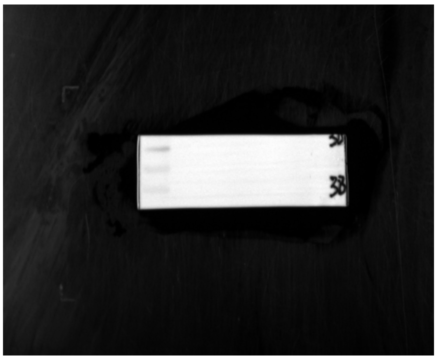

White light image

Chemiluminescent image

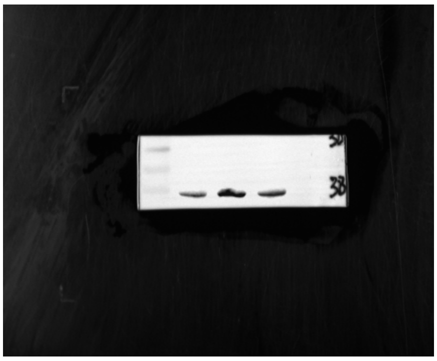

Composite image

Figure3F-HT22-BDNF Figure3F-HT22-Bax

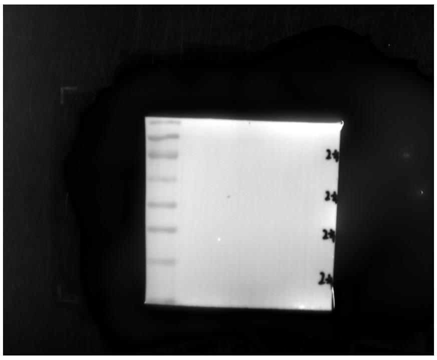 Full membrane image	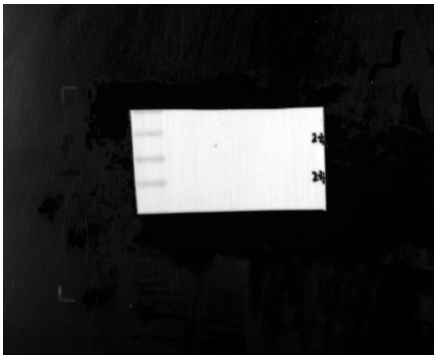 White light image
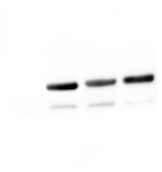 Chemiluminescent image	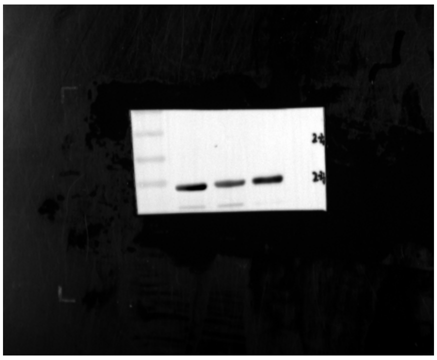 Composite image

Figure3F-HT22-β-actin

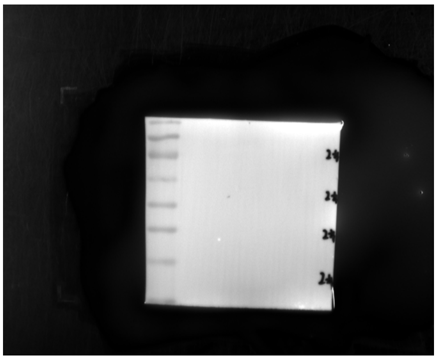 Full membrane image-同 Figure3F-HT22-Bax	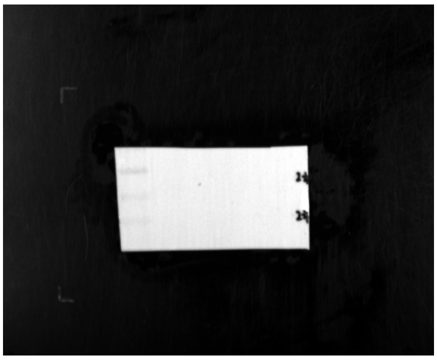 White light image
Chemiluminescent image	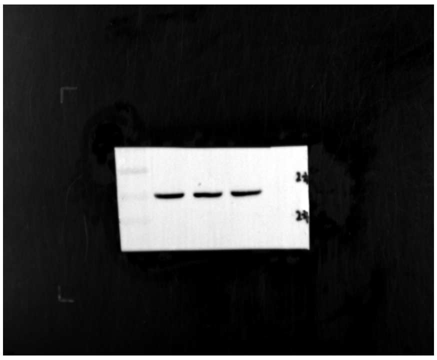 Composite image

Figure3F-BV2-Bax

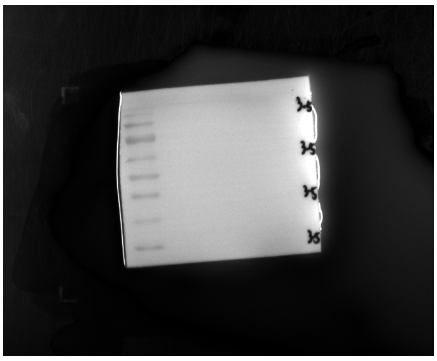 Full membrane image	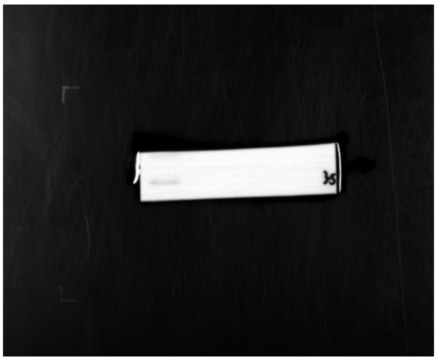 White light image
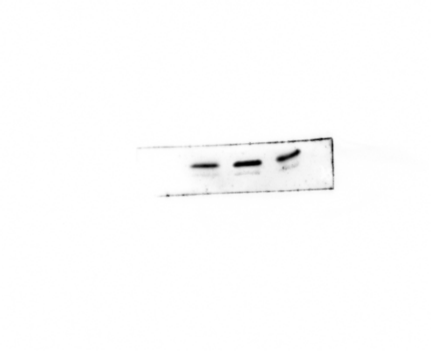 Chemiluminescent image	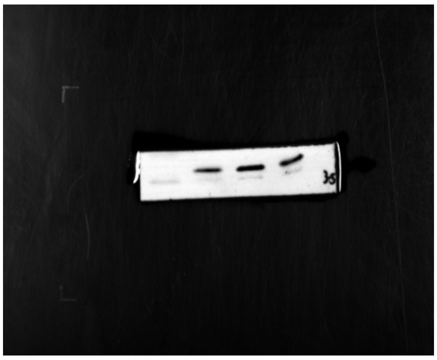 Composite image

Figure3F-BV2-Bcl2

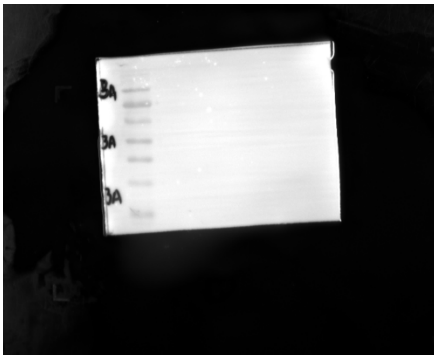 Full membrane image	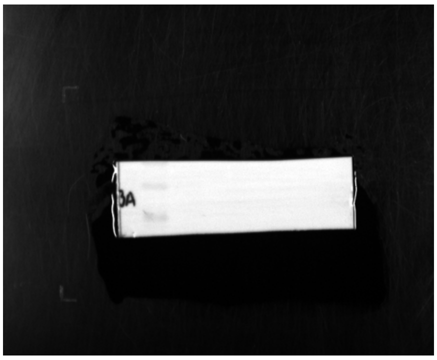 White light image
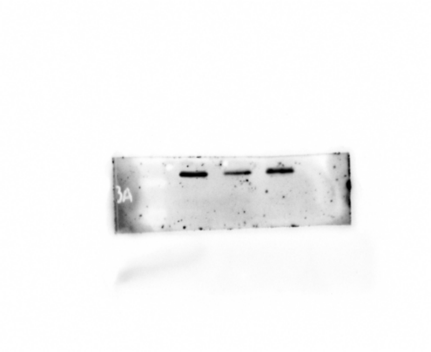 Chemiluminescent image	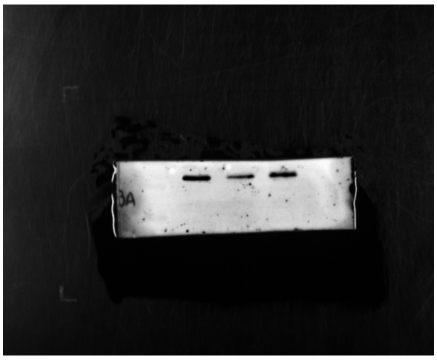 Composite image

Figure3F-BV2-Caspase3

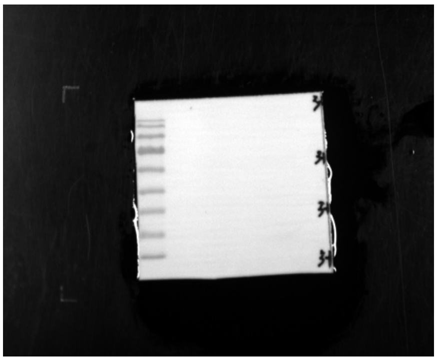
Full membrane image

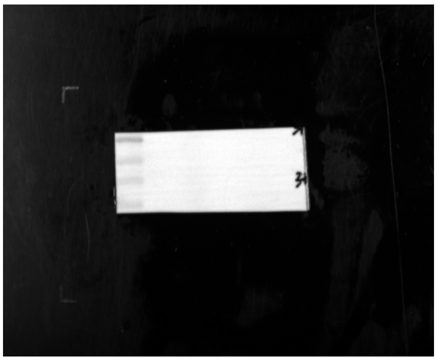
White light image

Chemiluminescent image

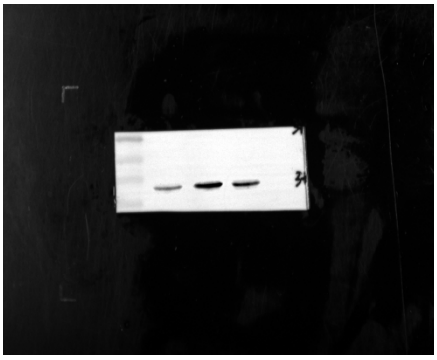
Composite image

Figure3F-BV2-BDNF

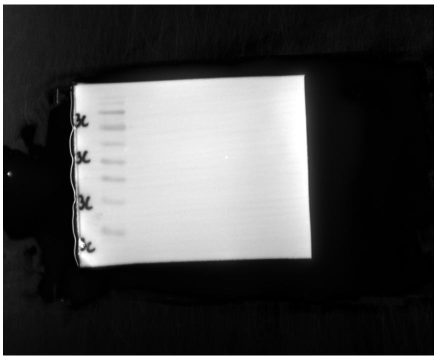
Full membrane image

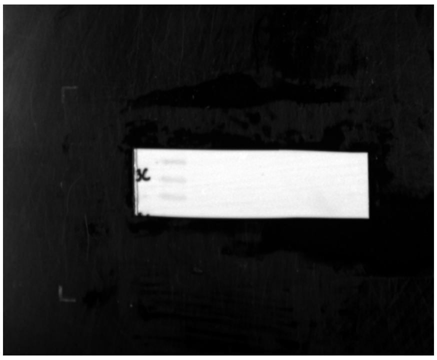
White light image

Chemiluminescent image

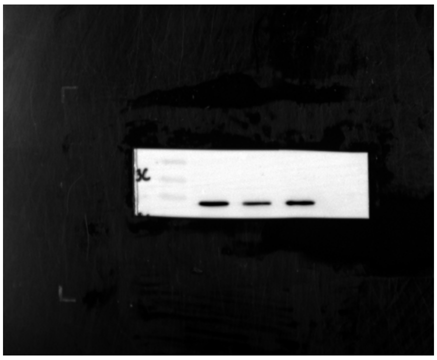
Composite image

Figure3F-BV2-β-actin

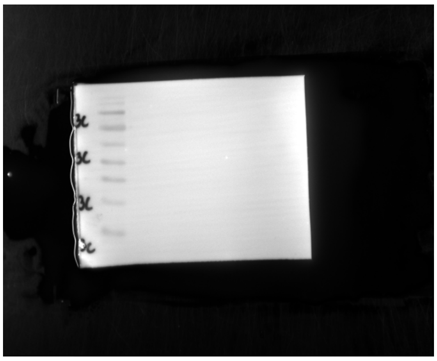<br>Full membrane image-同 Figure3F-BV2-BDNF	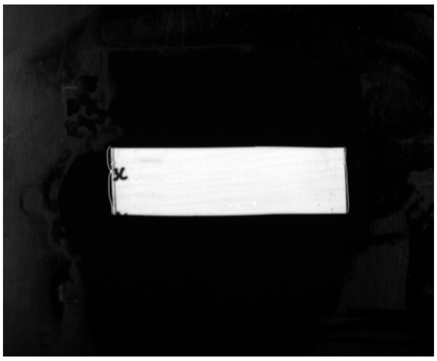<br>White light image
 Chemiluminescent image	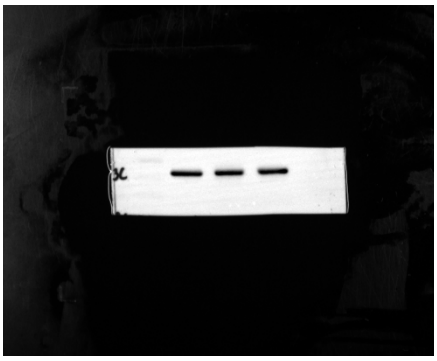<br>Composite image

Figure4E

Figure4E-Foxm1

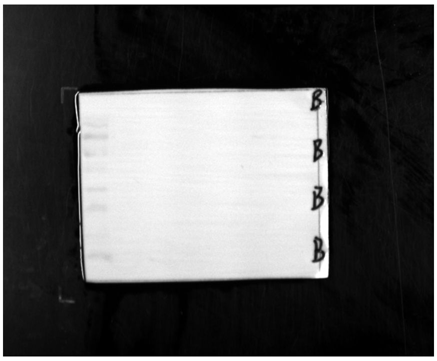<br>Full membrane image	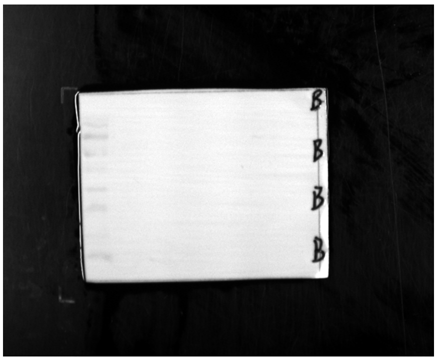<br>White light image

 Chemiluminescent image	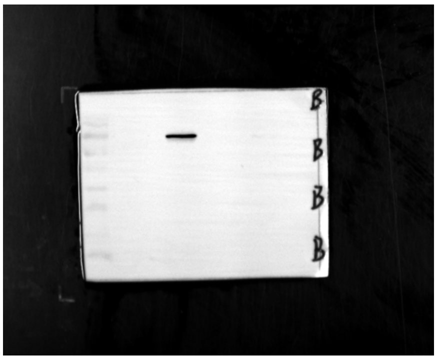<br>Composite image

Figure4F

Figure4F-BV2-Foxm1

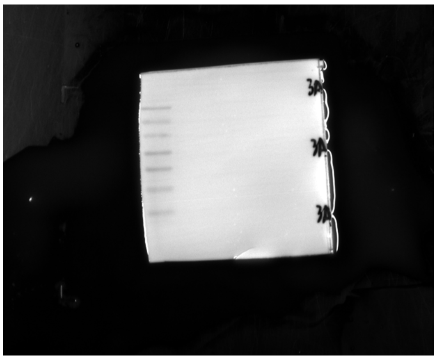<br>Full membrane image	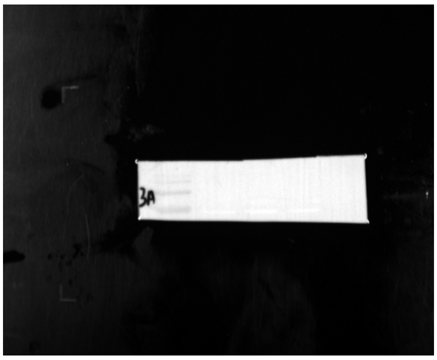<br>White light image
 Chemiluminescent image	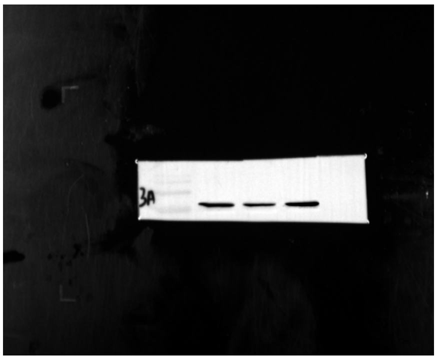<br>Composite image

Figure4F-BV2-β-actin Figure4F-BV2-Foxm1

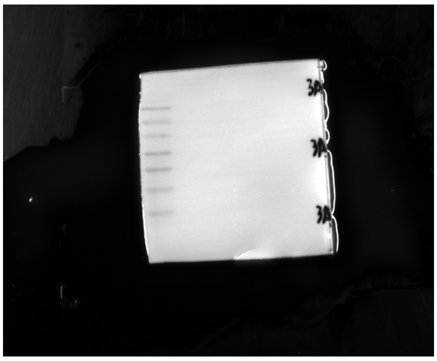	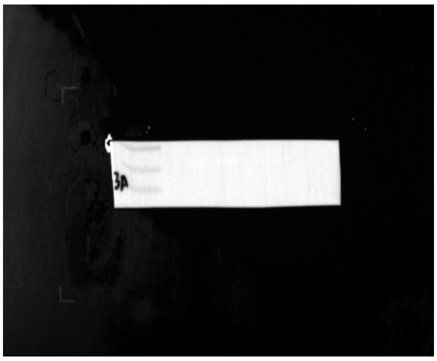

Full membrane image	White light image
	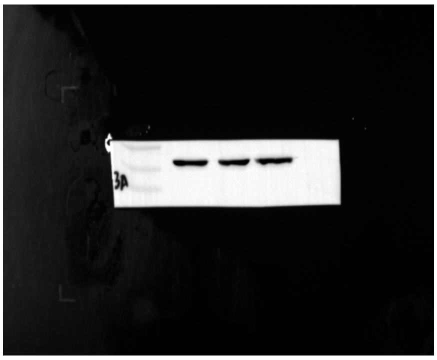
Chemiluminescent image	Composite image

Figure4F-HT22-Foxm1

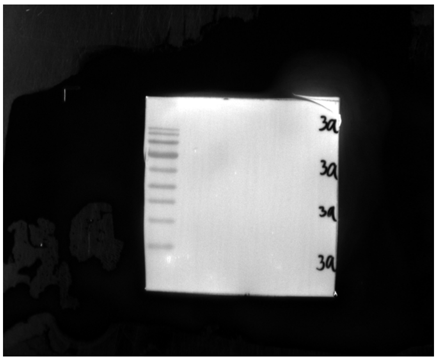	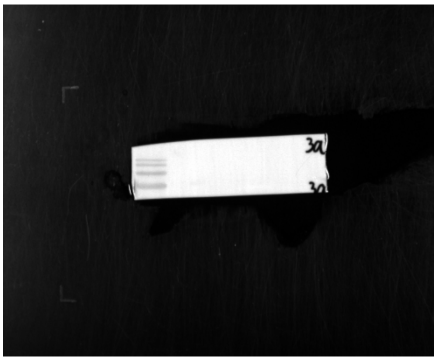
Full membrane image	White light image
	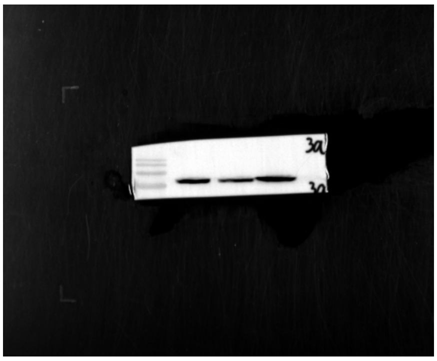
Chemiluminescent image	Composite image

Figure4F-HT22-β-actin Figure4F-HT22-Foxm1

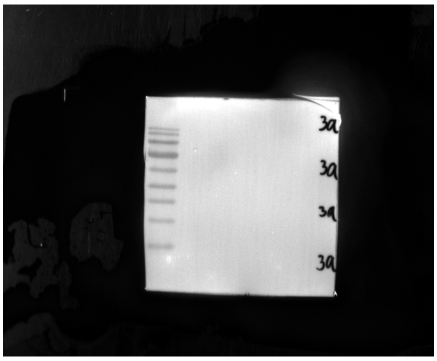	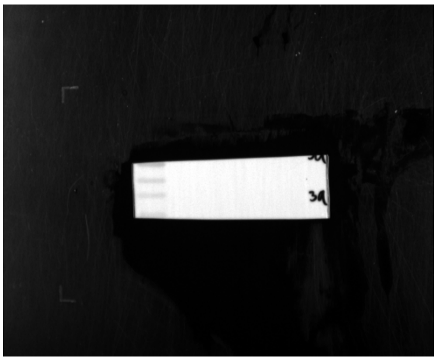

Full membrane image	White light image
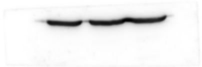 Chemiluminescent image	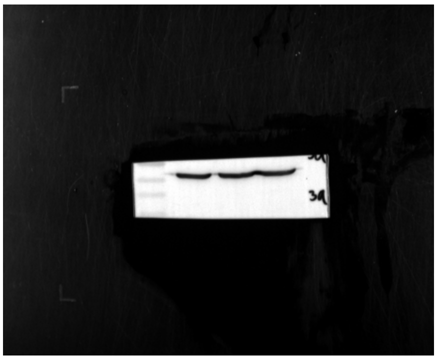 Composite image

Figure5A

Figure5A-Foxm1

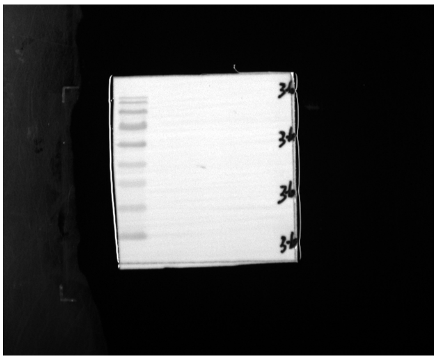 Full membrane image	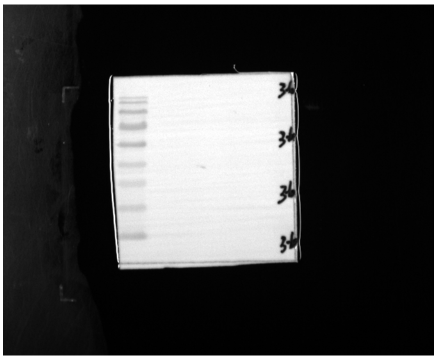 White light image
Chemiluminescent image	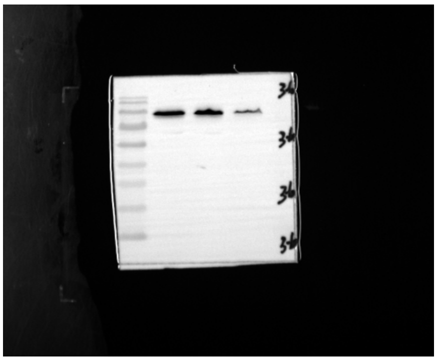 Composite image

Figure5A-β-actin

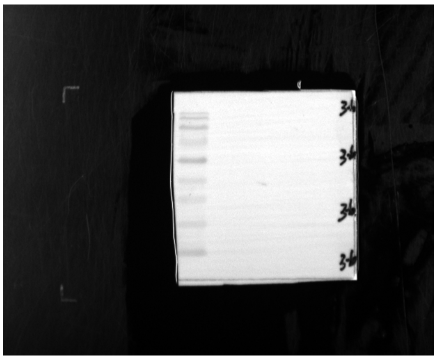<br>Full membrane image	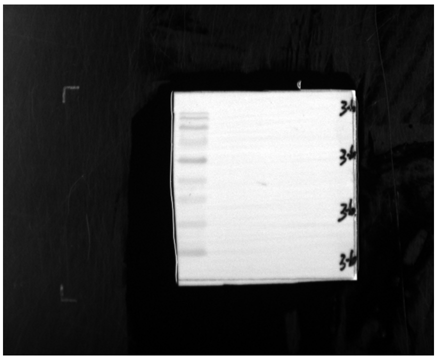<br>White light image
 Chemiluminescent image	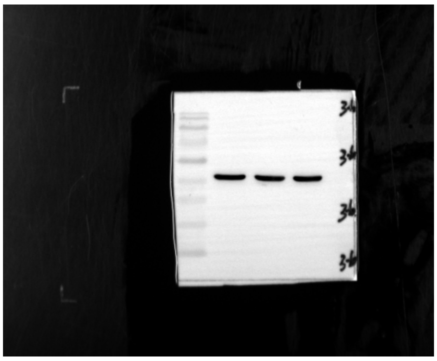<br>Composite image

Figure5B

Figure5B-Foxm1

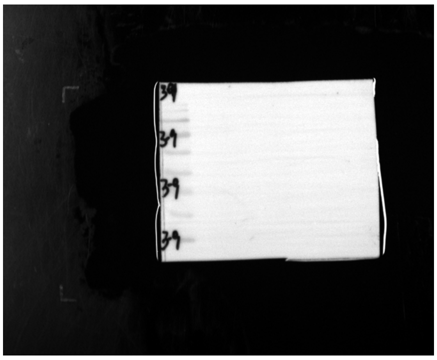<br>Full membrane image	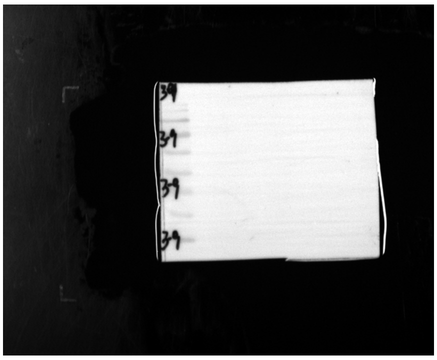<br>White light image
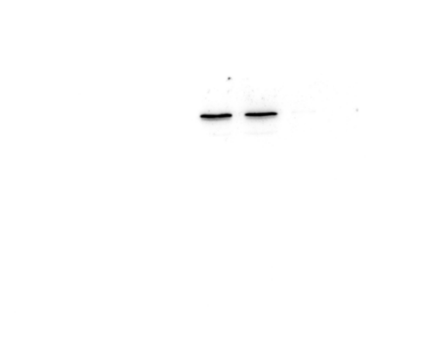<br>Chemiluminescent image	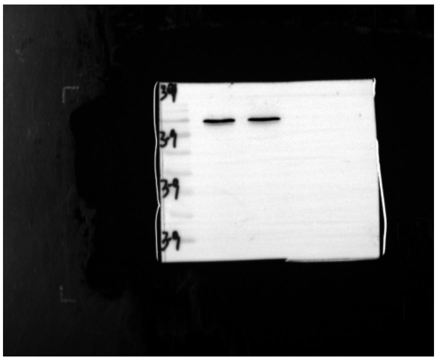<br>Composite image

Figure5C

Figure5C-HT22-Foxm1

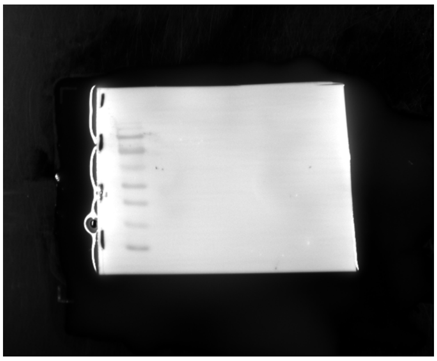<br>Full membrane image	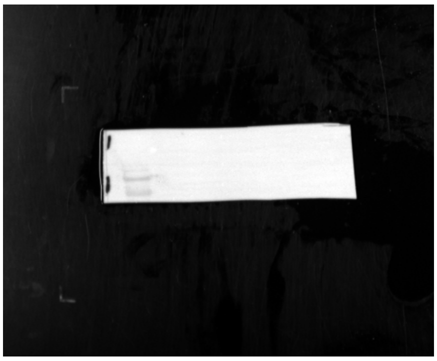<br>White light image
 Chemiluminescent image	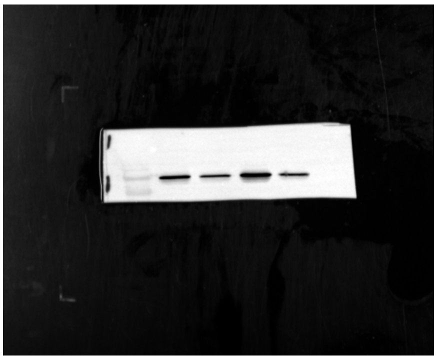<br>Composite image

Figure5C-HT22-β-actin

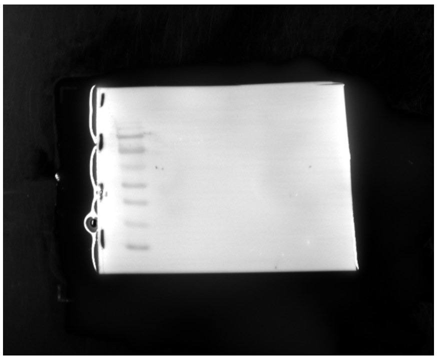<br>Full membrane image	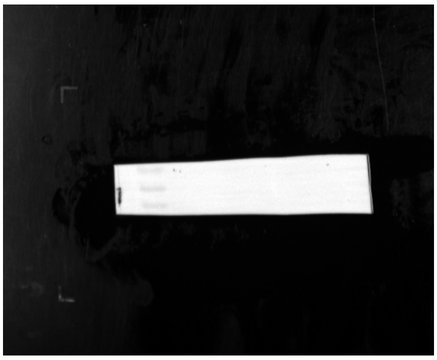<br>White light image
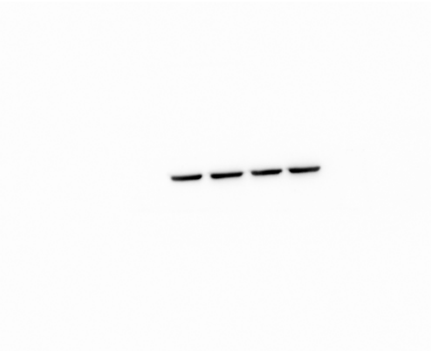<br>Chemiluminescent image	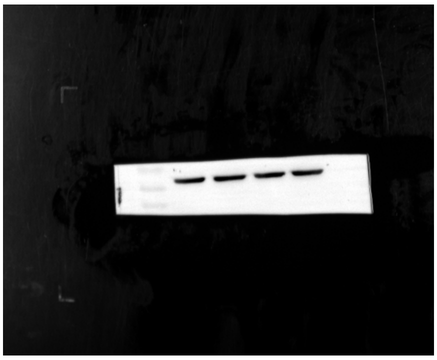<br>Composite image

Figure5D

Figure5D-BV2-Foxm1

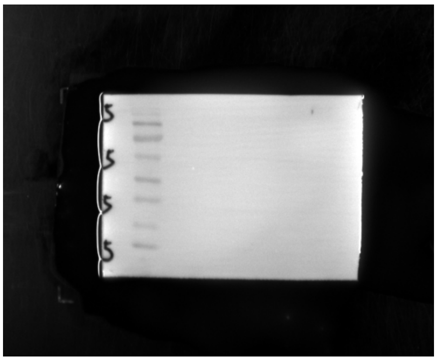<br>Full membrane image	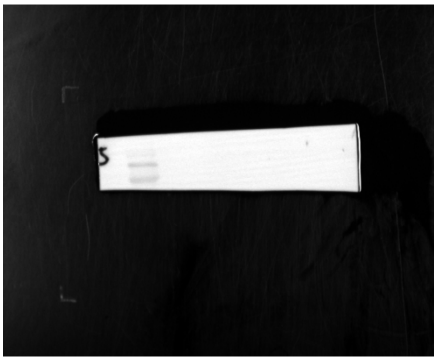<br>White light image
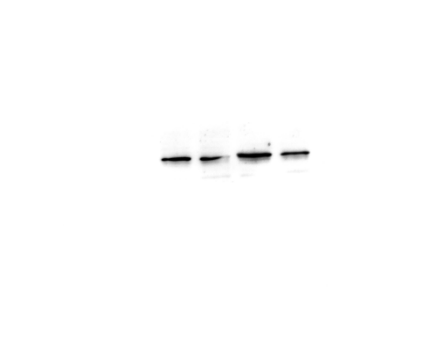<br>Chemiluminescent image	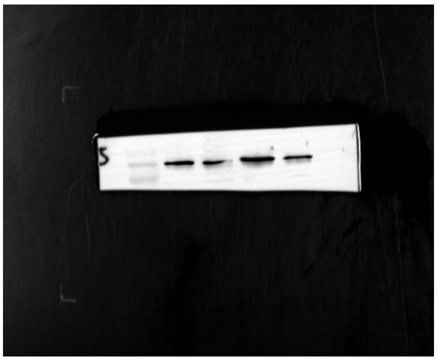<br>Composite image

Figure5D-BV2-β-actin

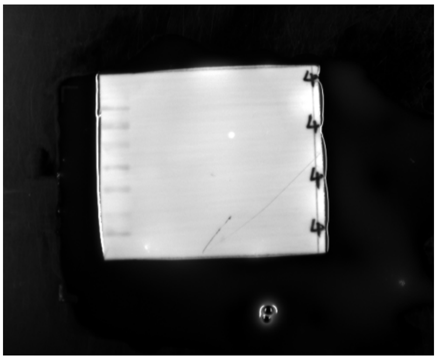<br>Full membrane image	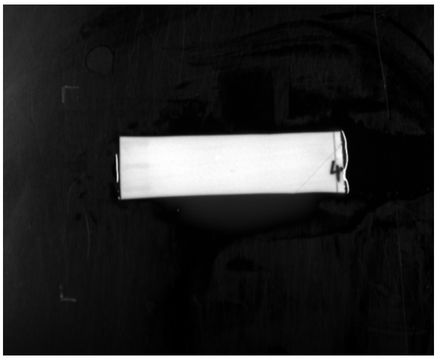<br>White light image
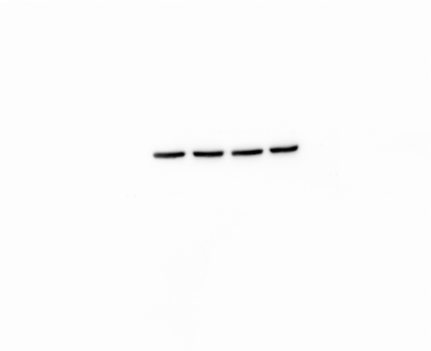	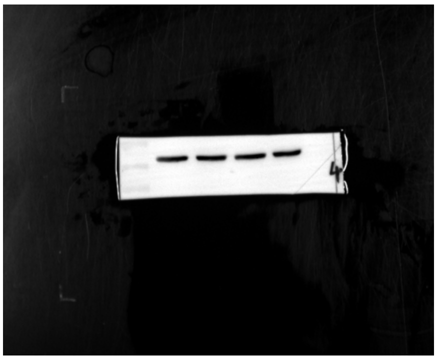

Chemiluminescent image	Composite image

Figure5I

Figure5I-HT22-Bax

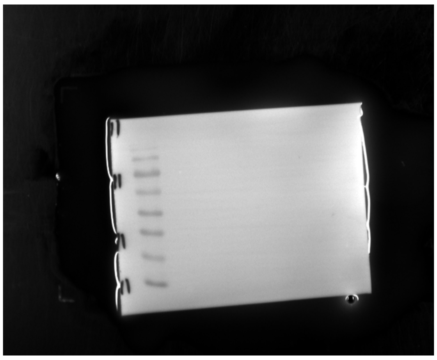<br>Full membrane image	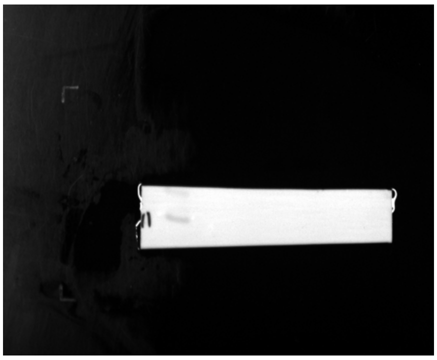<br>White light image
 Chemiluminescent image	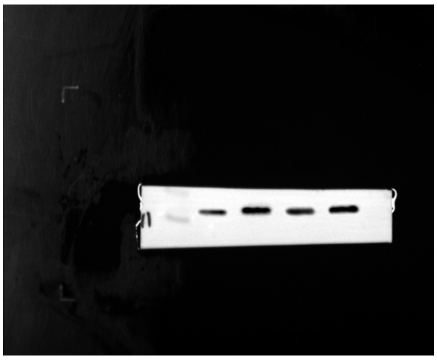<br>Composite image

Figure5I-HT22-Bcl2

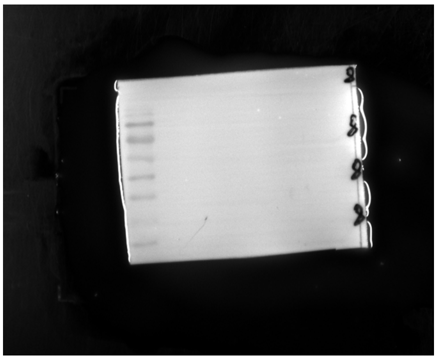<br>Full membrane image	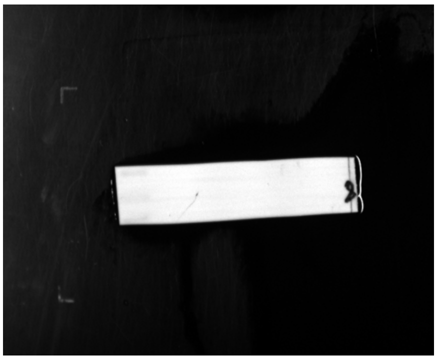<br>White light image

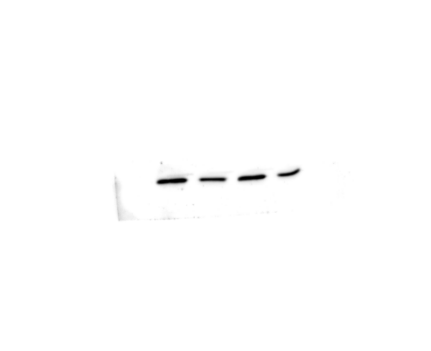<br>Chemiluminescent image	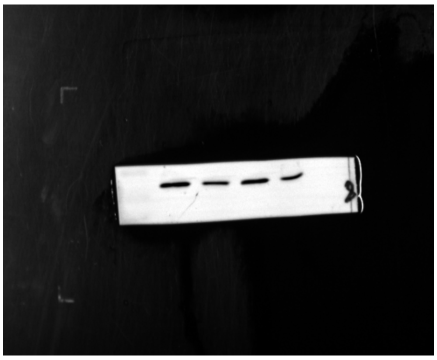<br>Composite image

Figure5I-HT22-Caspase3

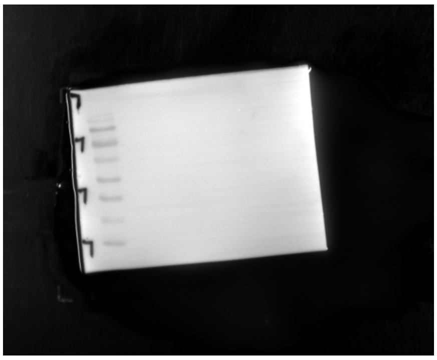<br>Full membrane image	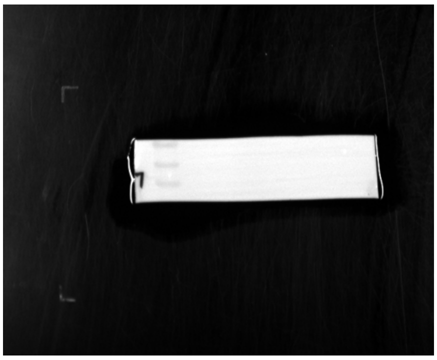<br>White light image
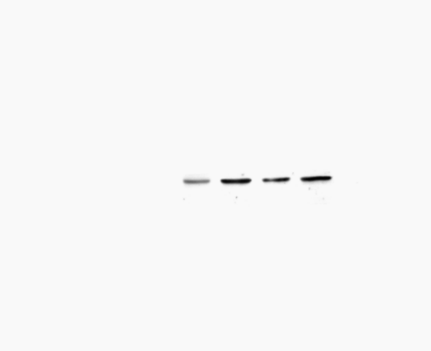<br>Chemiluminescent image	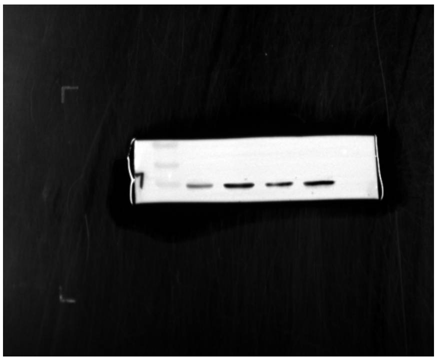<br>Composite image

Figure5I-HT22-BDNF

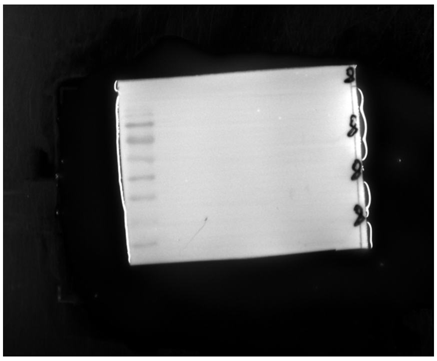<br>Full membrane image	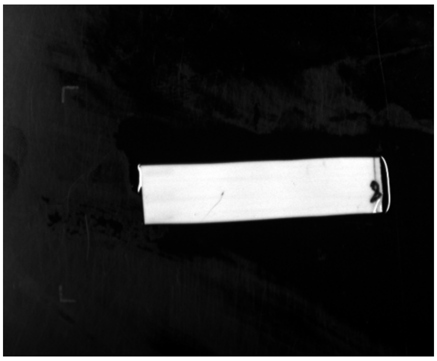<br>White light image

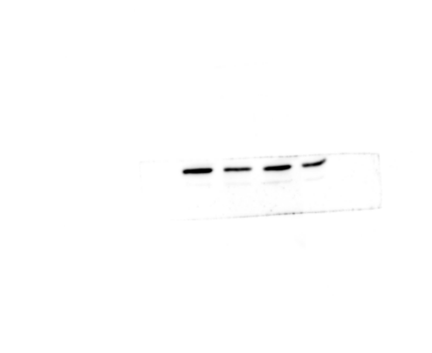<br>Chemiluminescent image	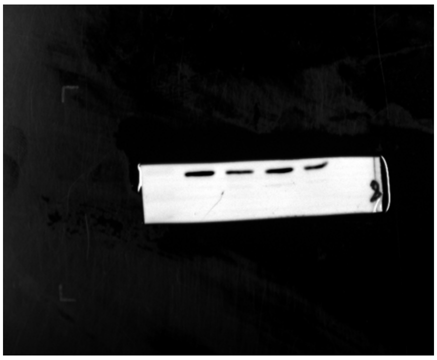<br>Composite image

Figure5I-HT22-β-actin

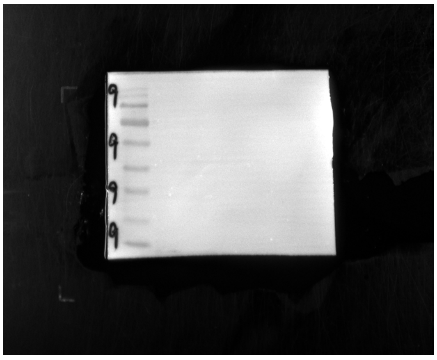<br>Full membrane image	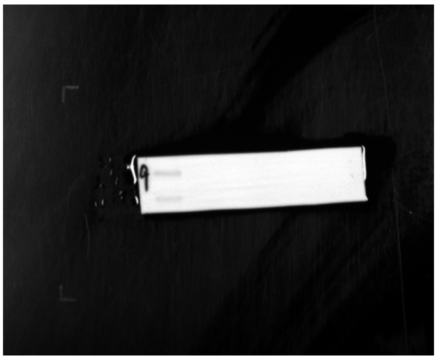<br>White light image
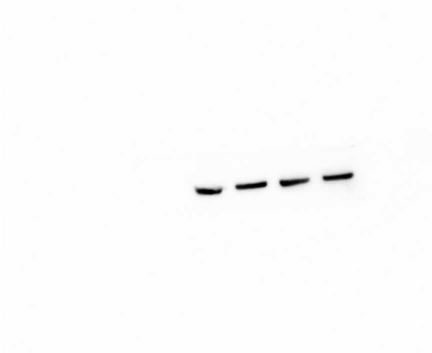<br>Chemiluminescent image	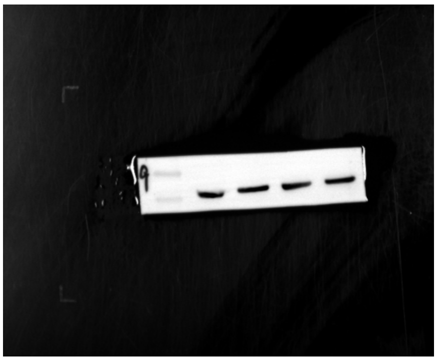<br>Composite image

Figure5J

Figure5J-BV2-Bax

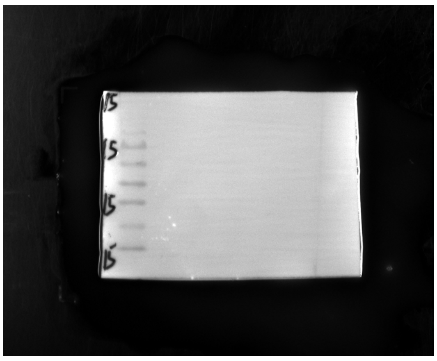	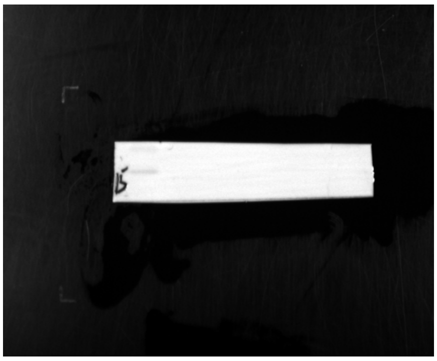

Full membrane image	White light image
Chemiluminescent image	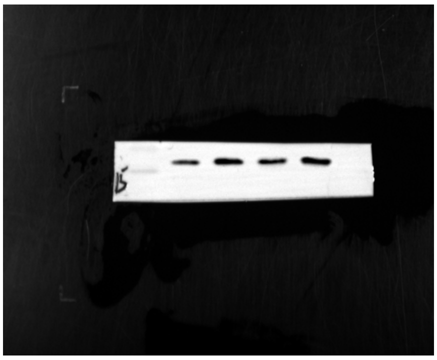 Composite image

Figure5J-BV2-Bcl2

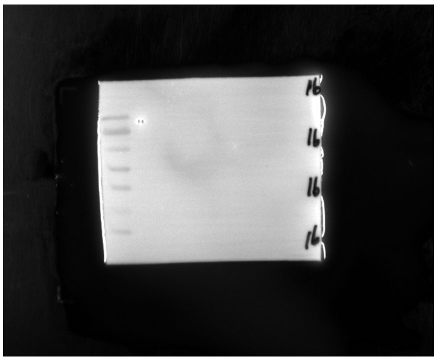 Full membrane image	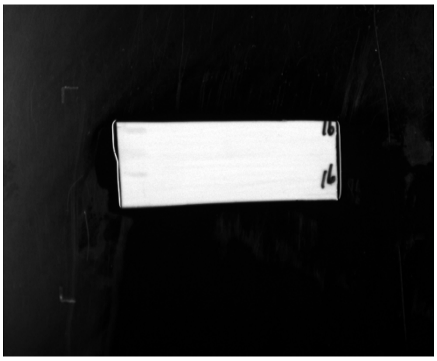 White light image
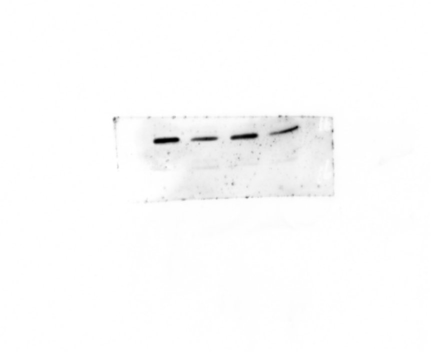 Chemiluminescent image	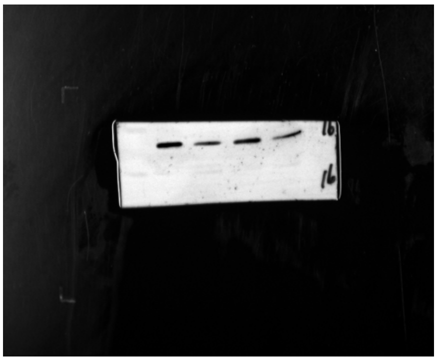 Composite image

Figure5J-BV2-Caspase3

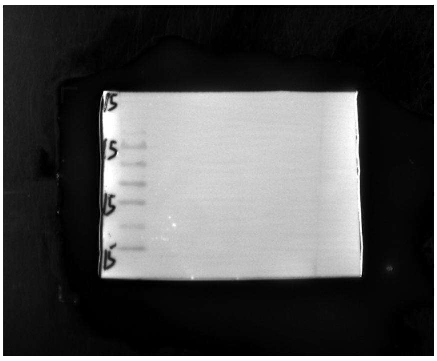	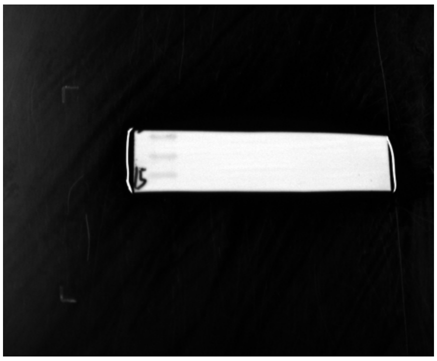

Full membrane image	White light image
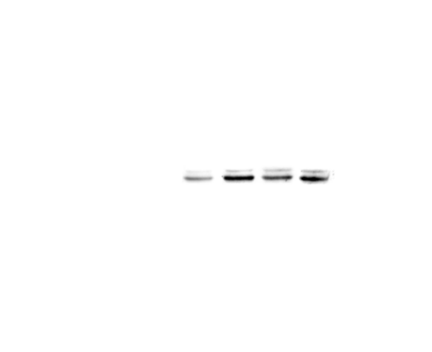 Chemiluminescent image	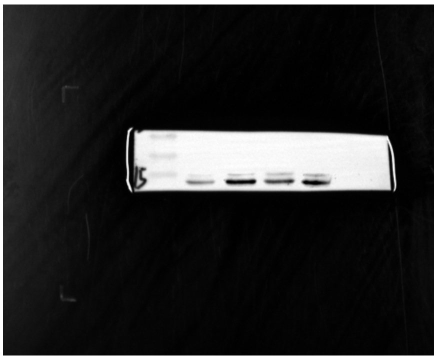 Composite image

Figure5J-BV2-BDNF

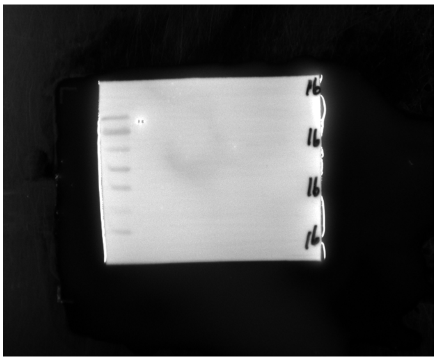 Full membrane image	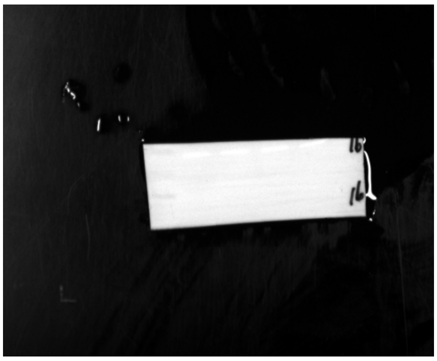 White light image
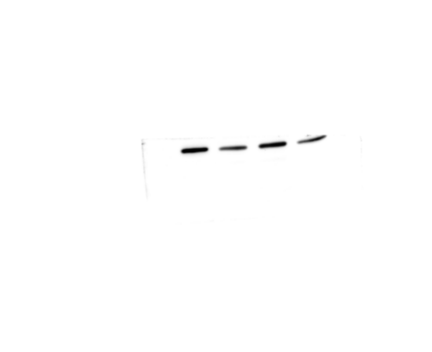 Chemiluminescent image	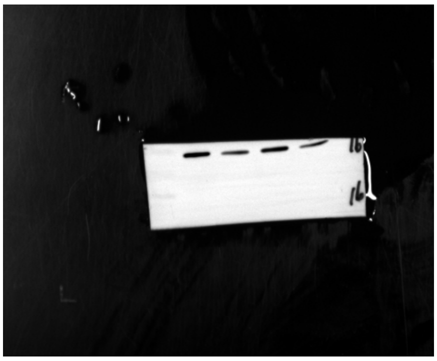 Composite image

Figure5J-BV2-β-actin

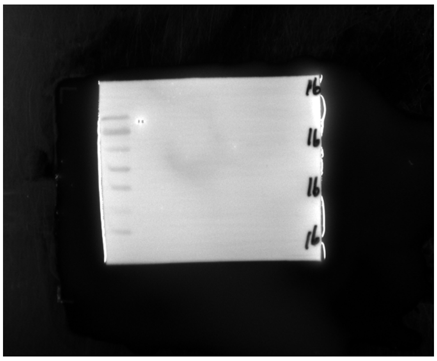	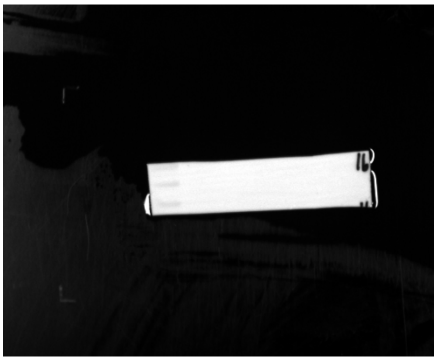

Full membrane image	White light image
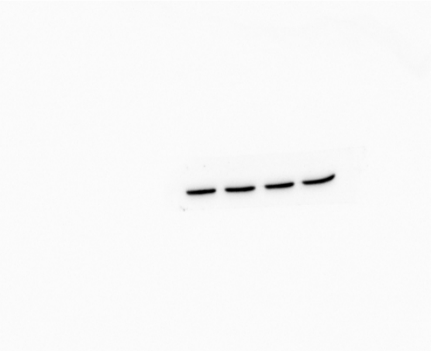<br>Chemiluminescent image	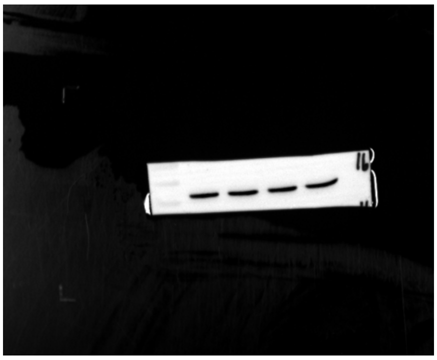<br>Composite image

Third replicate

Figure1D

Figure1D-HT22-Bax

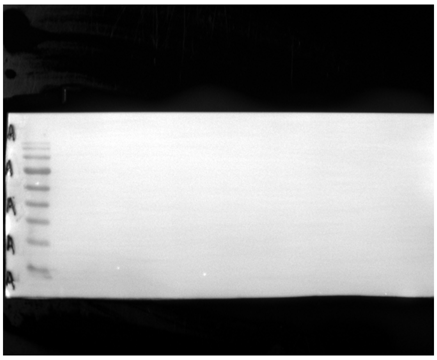<br>Full membrane image	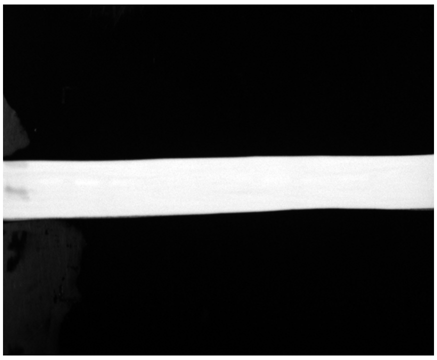<br>White light image
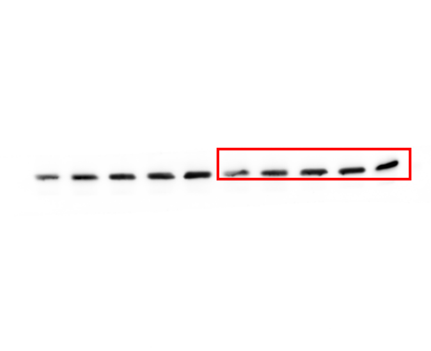<br>Chemiluminescent image	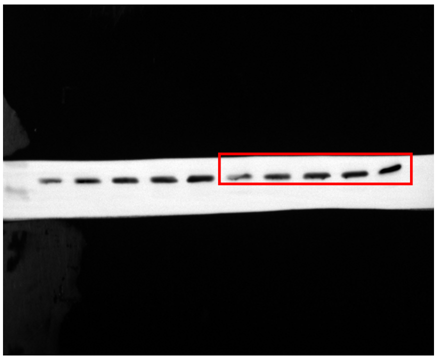<br>Composite image

Figure1D-HT22-Bcl2 Figure1D-HT22-Bax

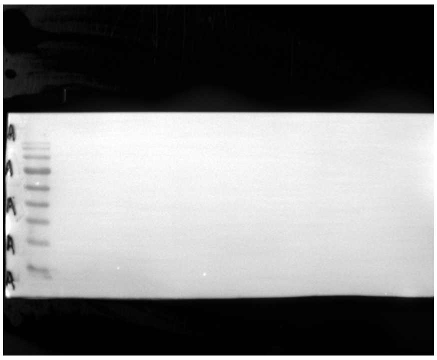

Full membrane image

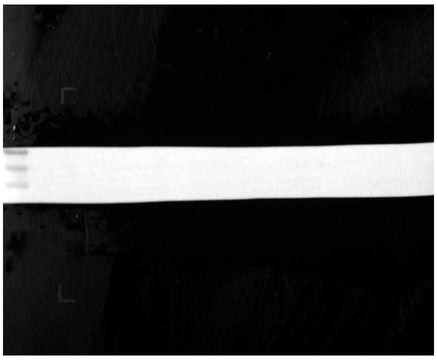

White light image

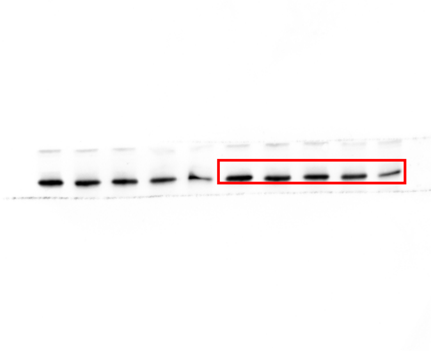

Chemiluminescent image

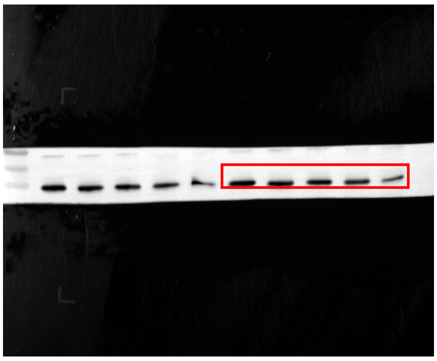

Composite image

Figure1D-HT22-Caspase3

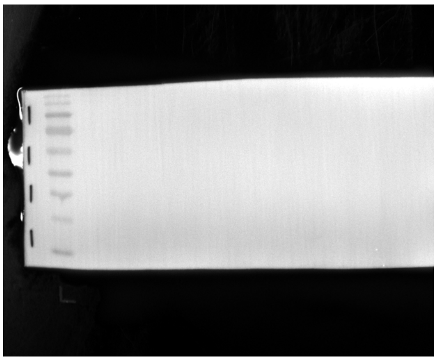

Full membrane image

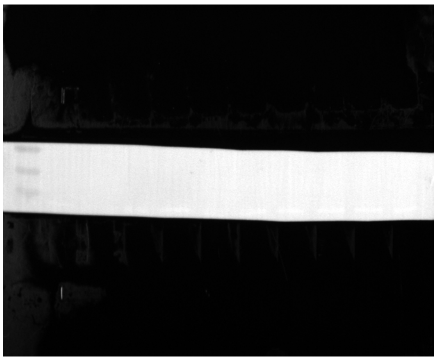

White light image

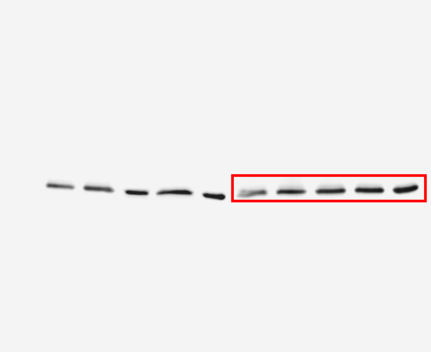

Chemiluminescent image

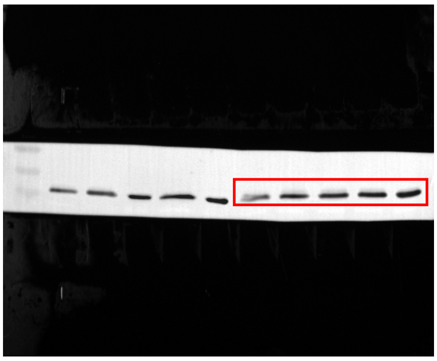

Composite image

Figure1D-HT22-BDNF

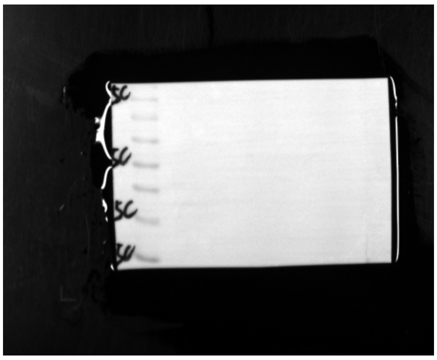<br>Full membrane image	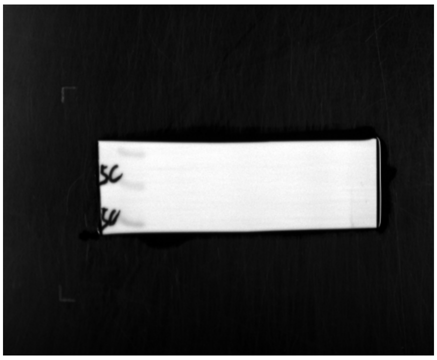<br>White light image
 Chemiluminescent image	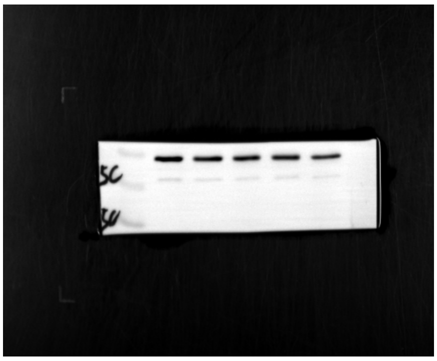<br>Composite image

Figure1D-HT22-β-actin Figure1D-HT22-Caspase3

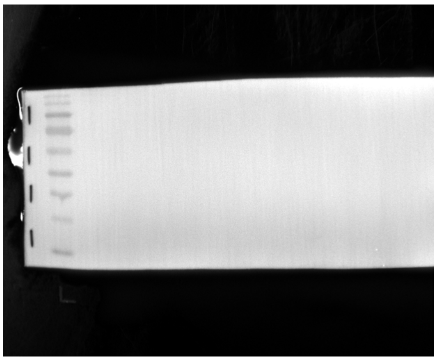<br>Full membrane image	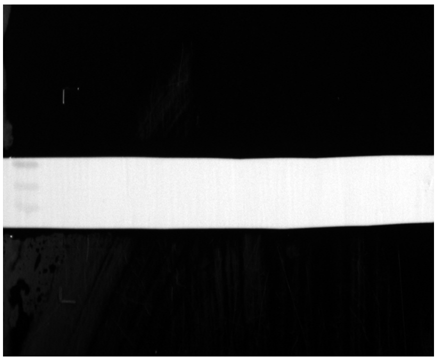<br>White light image
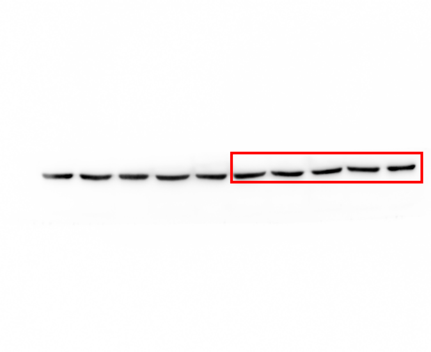<br>Chemiluminescent image	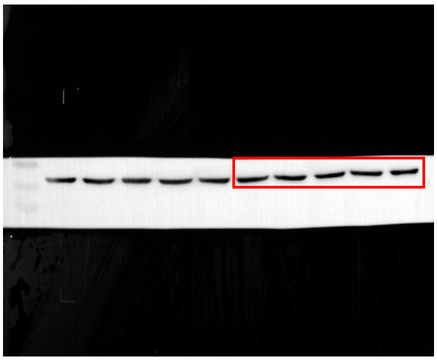<br>Composite image

Figure1D-BV2-Bax

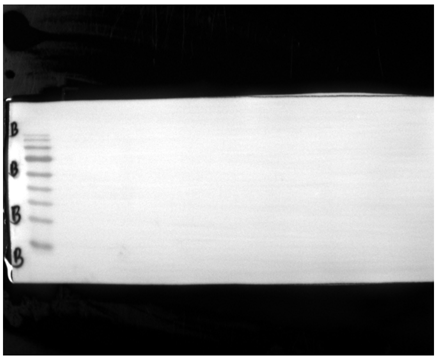 Full membrane image	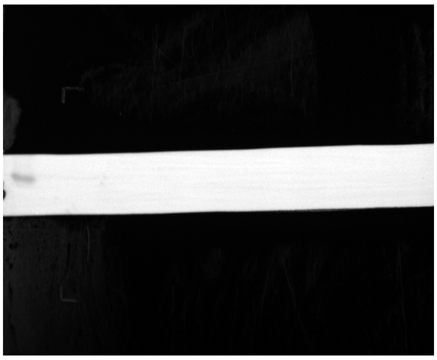 White light image
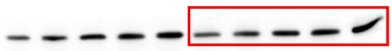 Chemiluminescent image	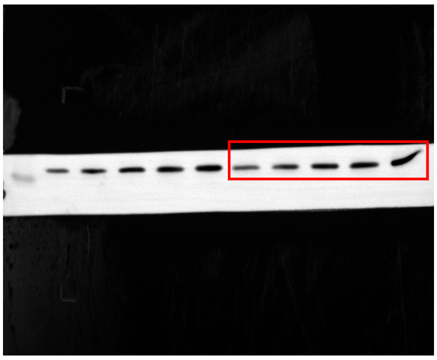 Composite image

Figure1D-BV2-Bcl2 Figure1D-BV2-Bax

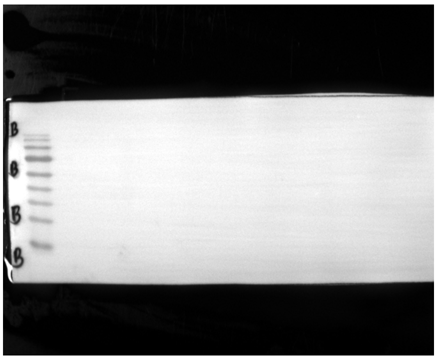 Full membrane image	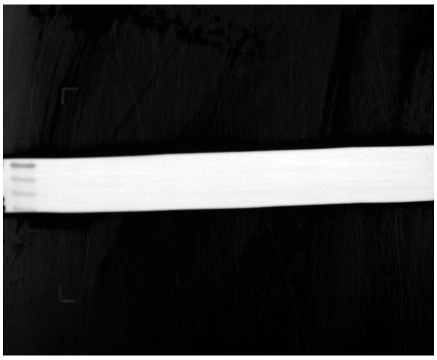 White light image
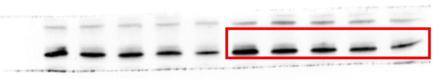 Chemiluminescent image	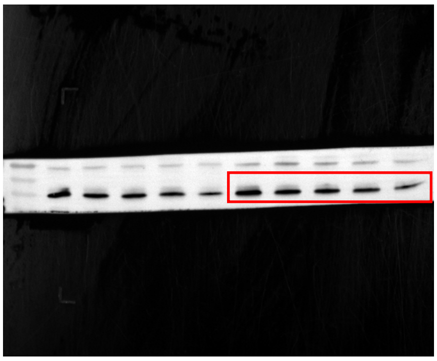 Composite image

Figure1D-BV2-Caspase3

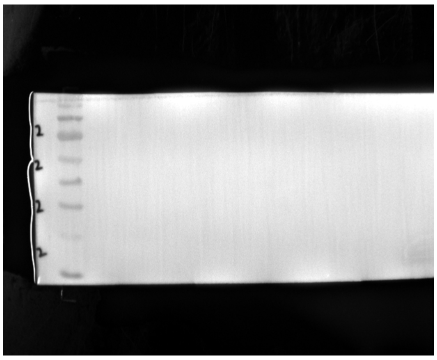

Full membrane image

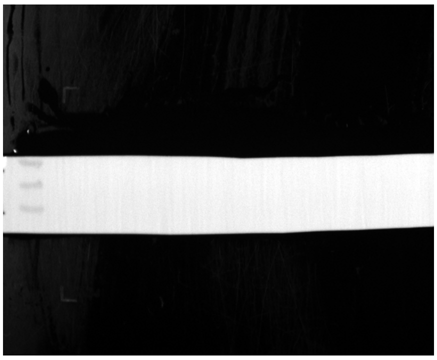

White light image

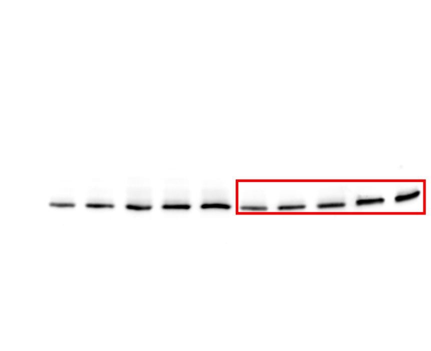

Chemiluminescent image

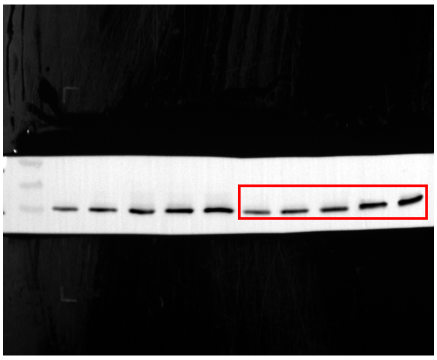

Composite image

Figure1D-BV2-BDNF

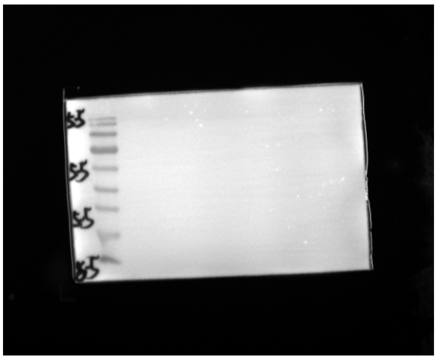

Full membrane image

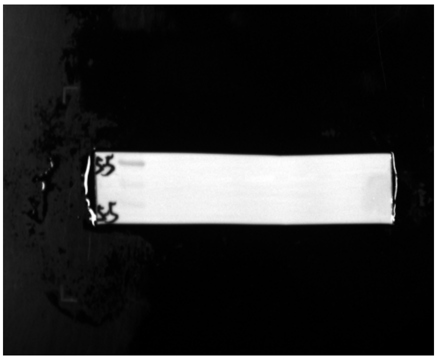

White light image

Chemiluminescent image

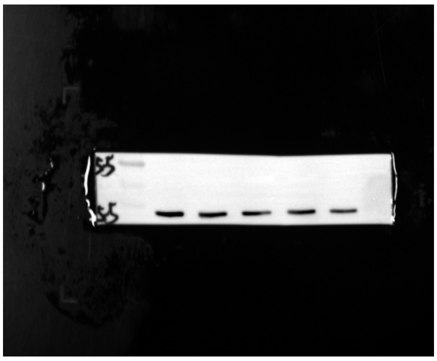

Composite image

Figure1D-BV2-β-actin Figure1D-BV2-Caspase3

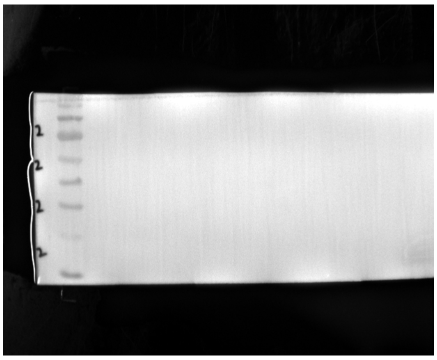 Full membrane image	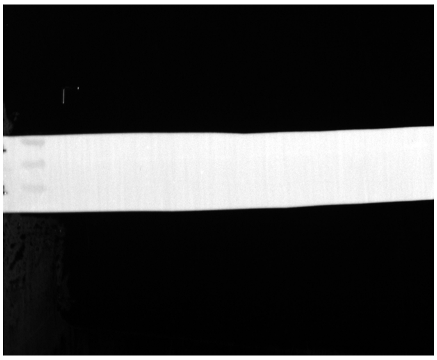 White light image
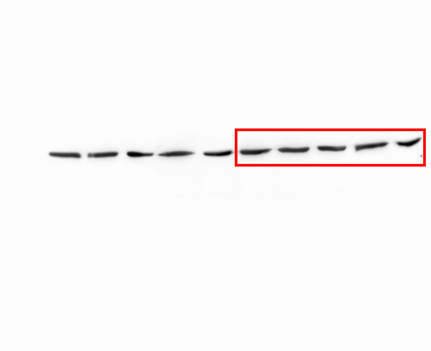 Chemiluminescent image	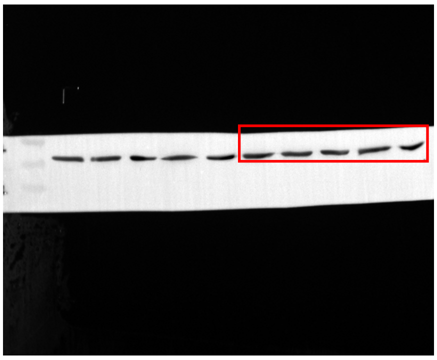 Composite image

Figure2A

Figure2A-CD63

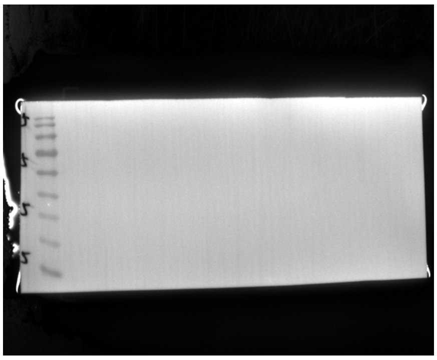 Full membrane image	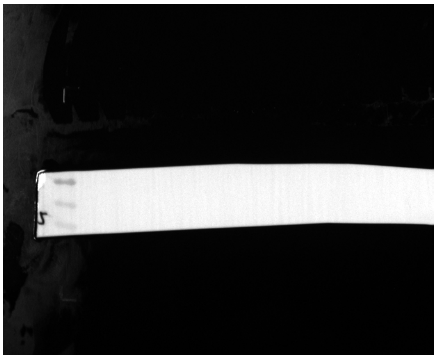 White light image
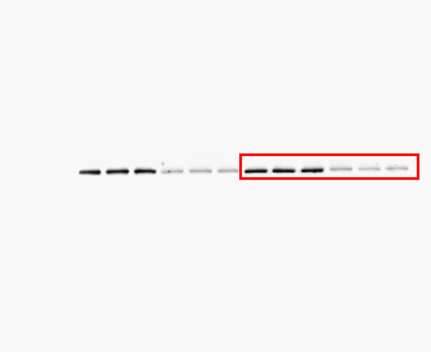 Chemiluminescent image	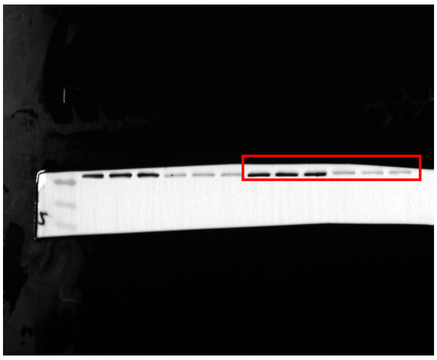 Composite image

Figure2A-TSG101

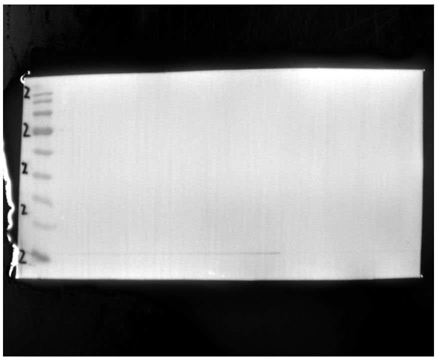

Full membrane image

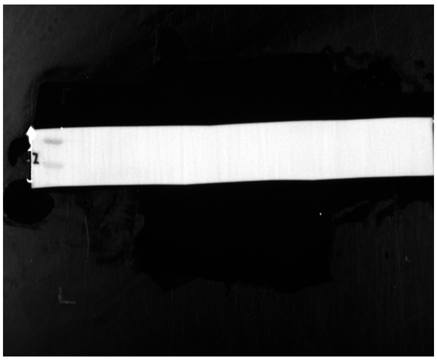

White light image

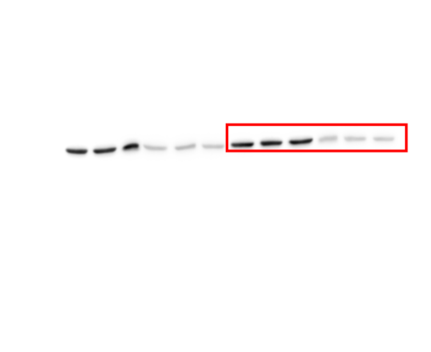

Chemiluminescent image

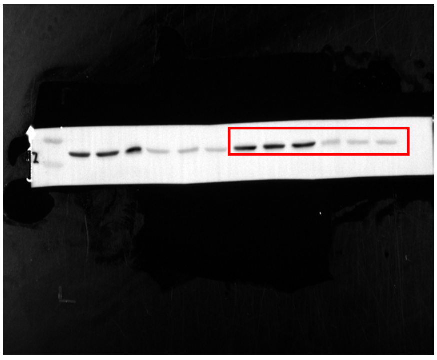

Composite image

Figure3F

Figure3F-HT22-Bax

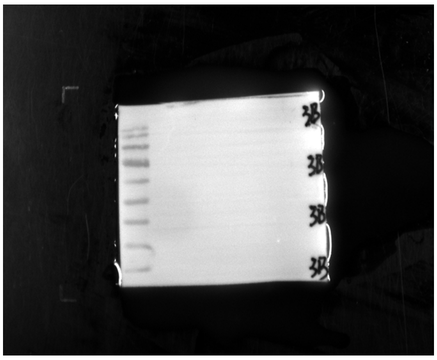

Full membrane image

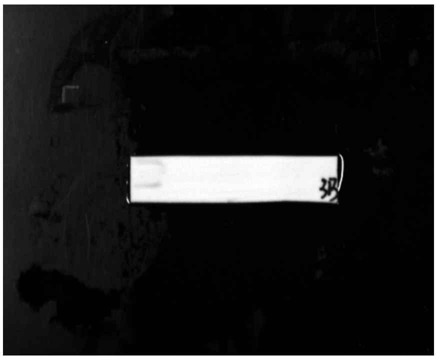

White light image

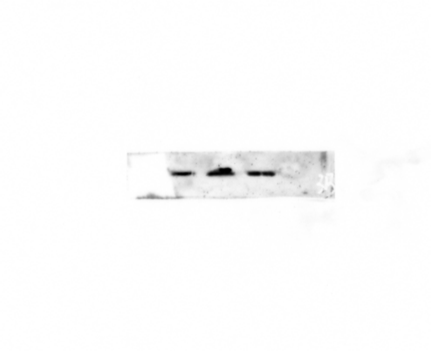

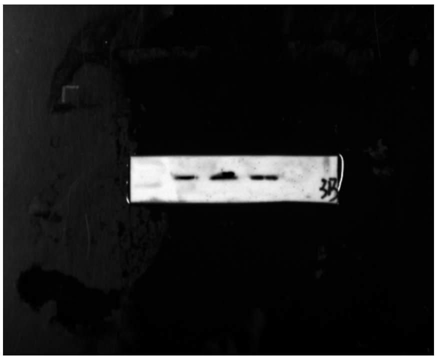

Chemiluminescent image	Composite image

Figure3F-HT22-Bcl2

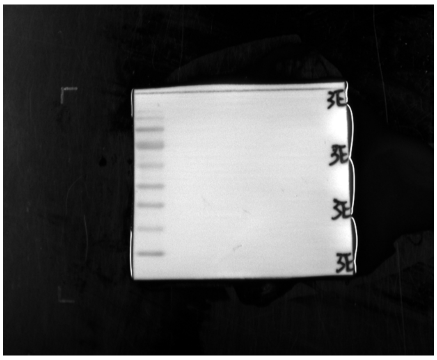 Full membrane image	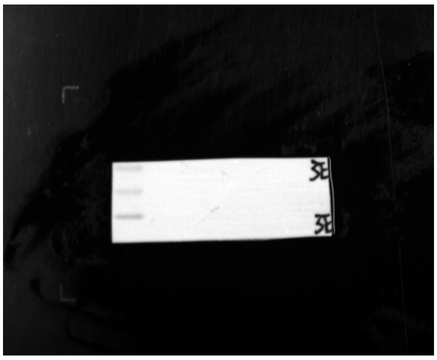 White light image
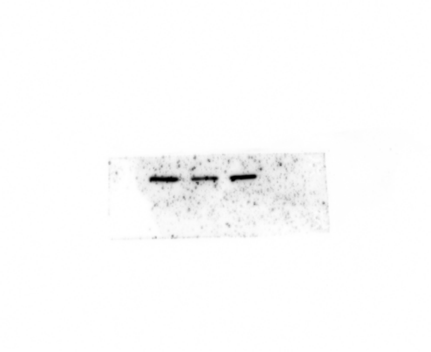 Chemiluminescent image	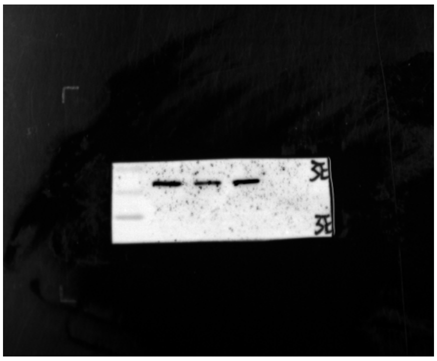 Composite image

Figure3F-HT22-Caspase3

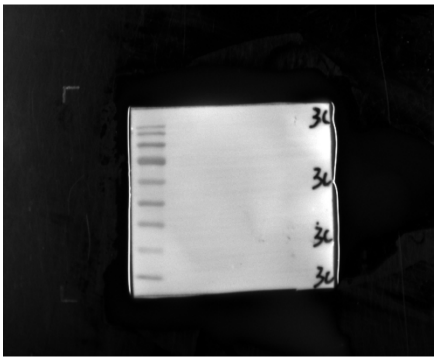 Full membrane image	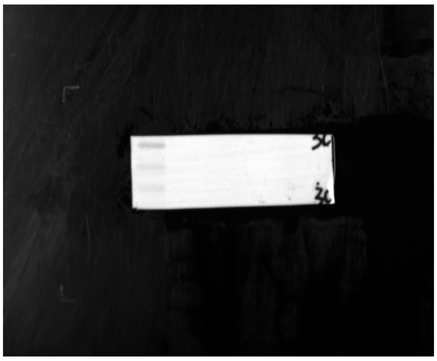 White light image
Chemiluminescent image	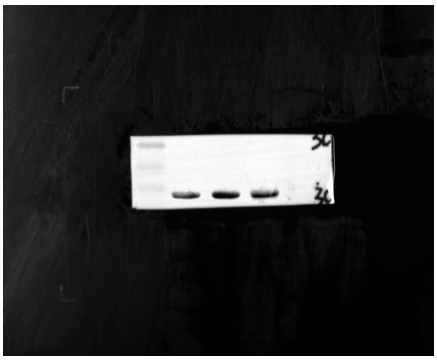 Composite image

Chemiluminescent image	Composite image

Figure3F-HT22-BDNF

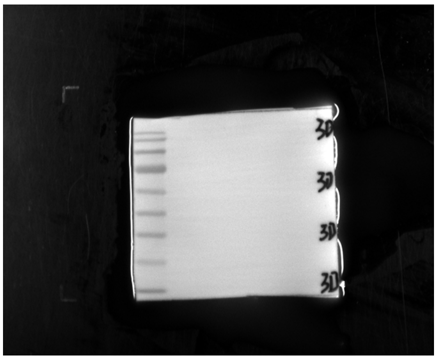<br>Full membrane image	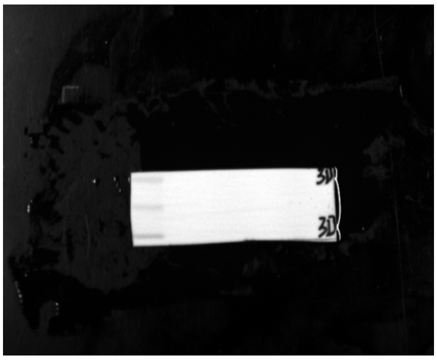<br>White light image
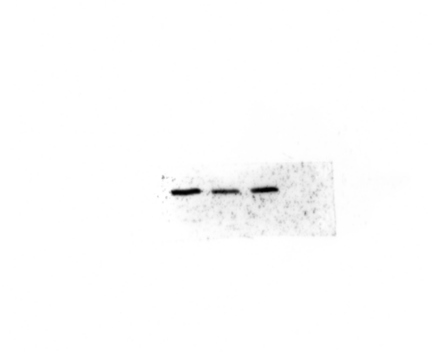<br>Chemiluminescent image	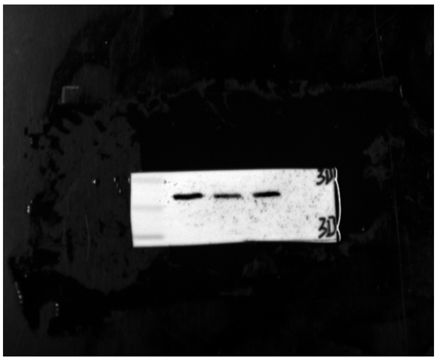<br>Composite image

Figure3F-HT22-β-actin Figure3F-HT22-BDNF

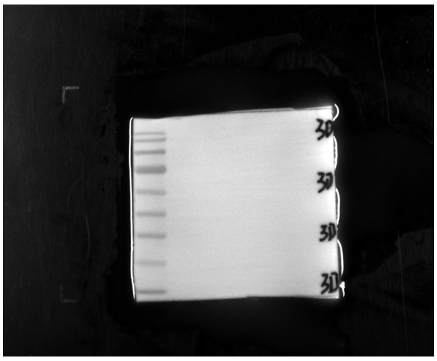<br>Full membrane image	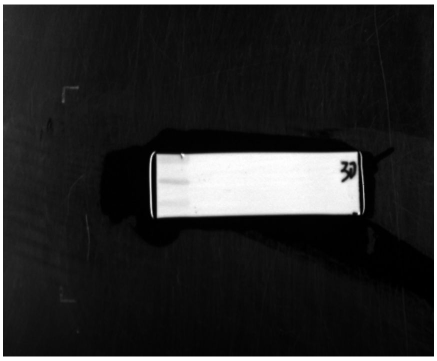<br>White light image
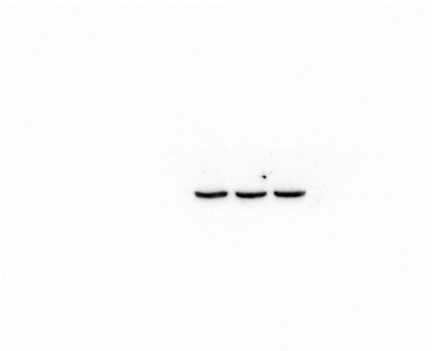	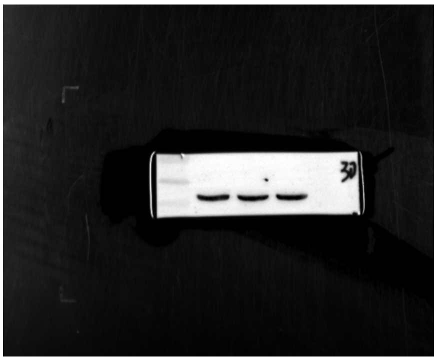

Chemiluminescent image	Composite image

Figure3F-BV2-Bax

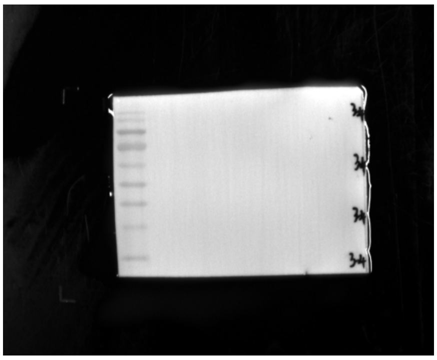<br>Full membrane image	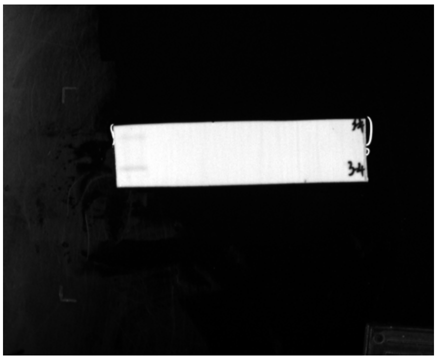<br>White light image
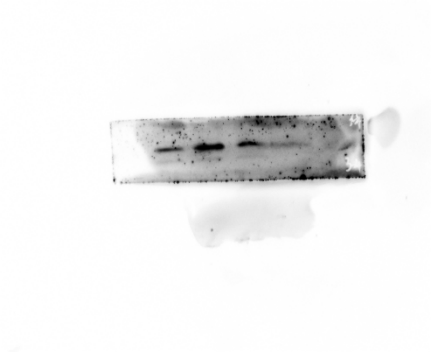<br>Chemiluminescent image	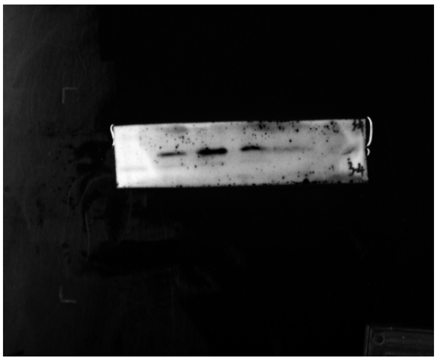<br>Composite image

Figure3F-BV2-Bcl2

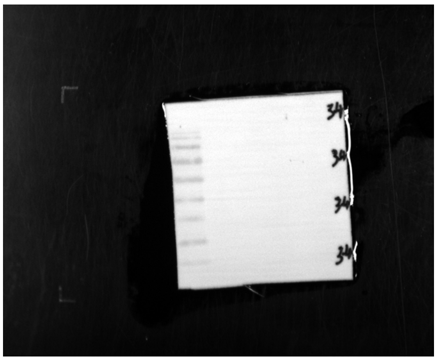<br>Full membrane image	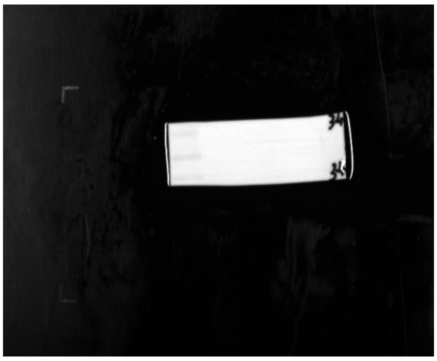<br>White light image
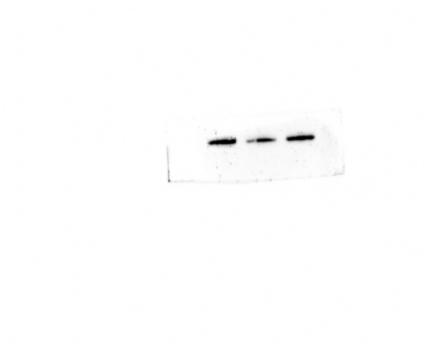	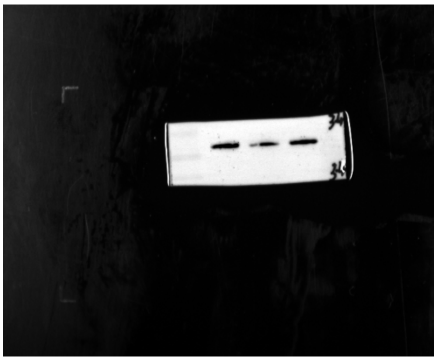

Chemiluminescent image	Composite image

Figure3F-BV2-Caspase3

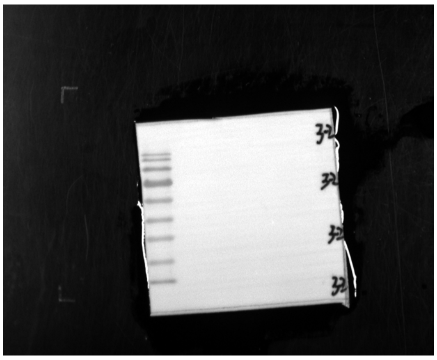<br>Full membrane image	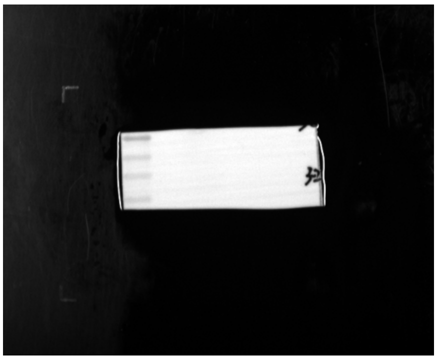<br>White light image
 Chemiluminescent image	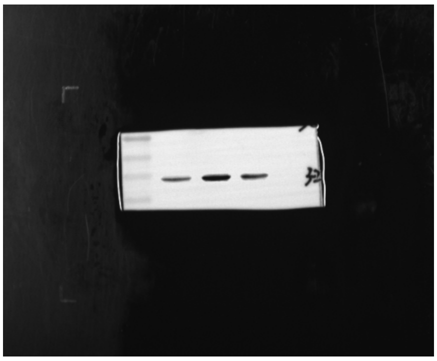<br>Composite image

Figure3F-BV2-BDNF

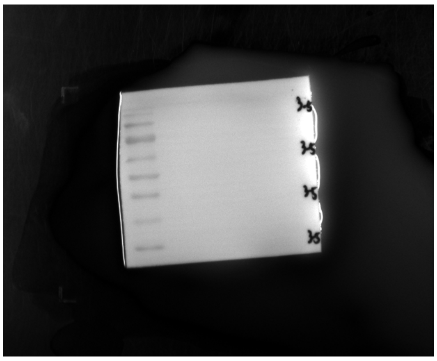<br>Full membrane image	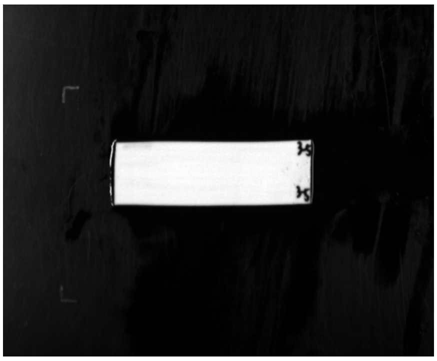<br>White light image
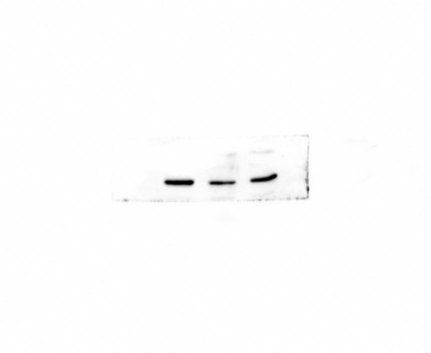	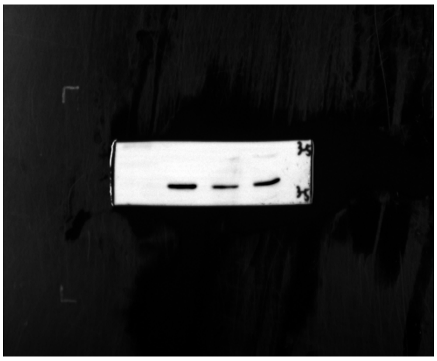

Chemiluminescent image	Composite image

Figure3F-BV2-β-actin

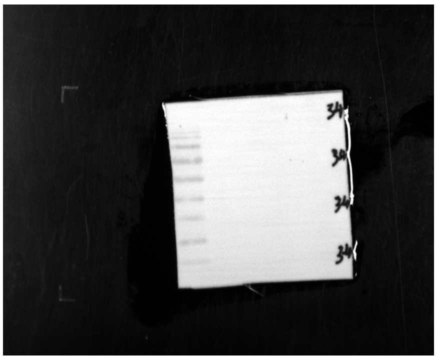<br>Full membrane image-同 Figure3F-BV2-Bcl2	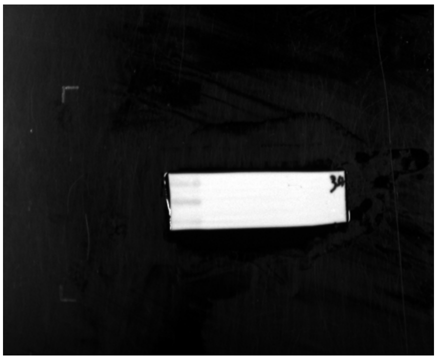<br>White light image
 Chemiluminescent image	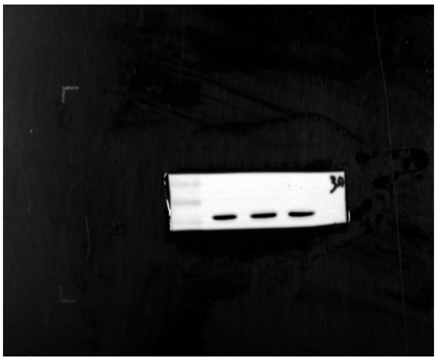<br>Composite image

Figure4E

Figure4E-Foxm1

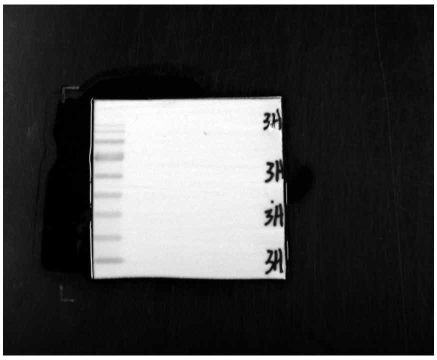<br>Full membrane image	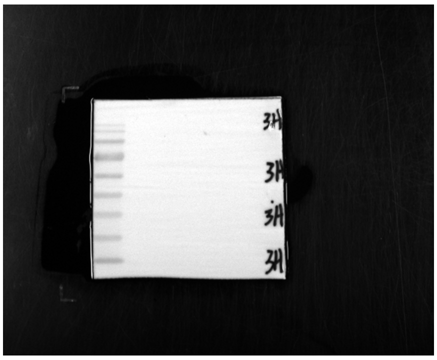<br>White light image

 Chemiluminescent image	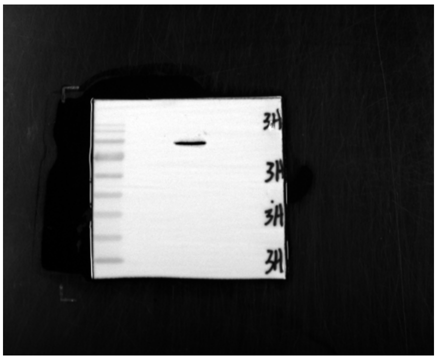<br>Composite image

Figure4F

Figure4F-BV2-Foxm1

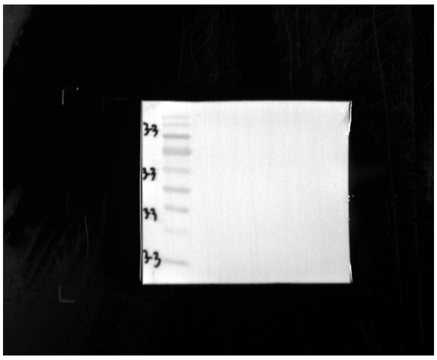<br>Full membrane image	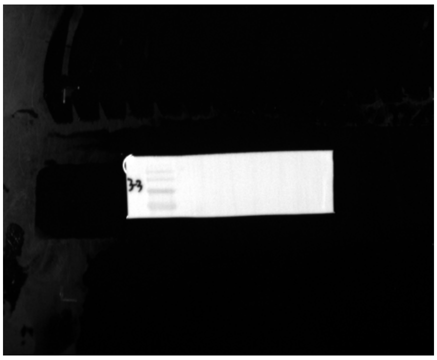<br>White light image
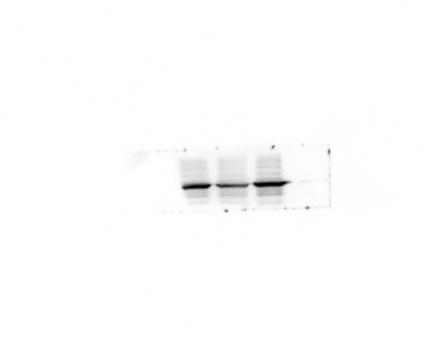<br>Chemiluminescent image	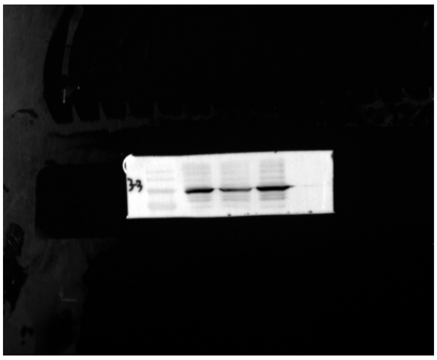<br>Composite image

Figure4F-BV2-β-actin Figure4F-BV2-β-actin

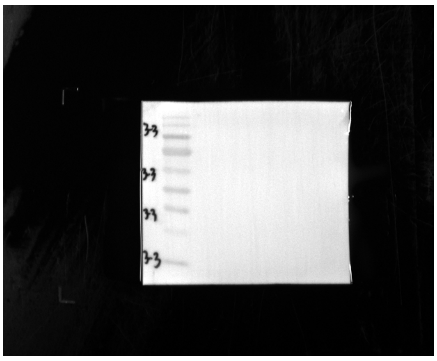	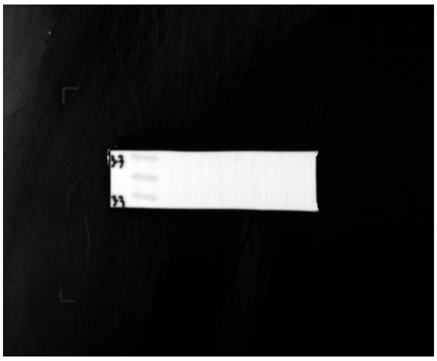

Full membrane image	White light image
 Chemiluminescent image	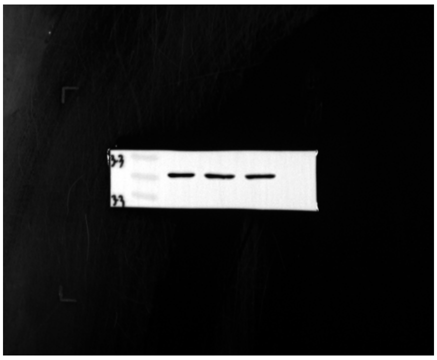<br>Composite image

Figure4F-HT22-Foxm1

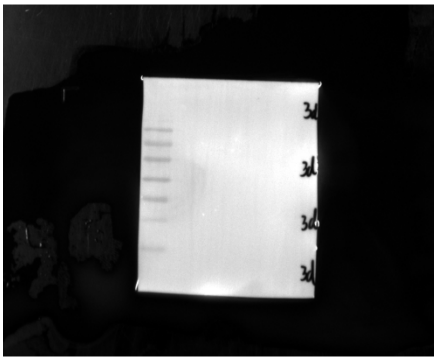<br>Full membrane image	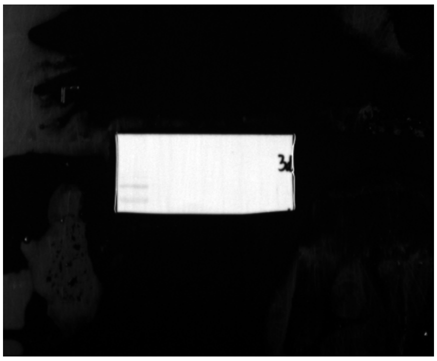<br>White light image
 Chemiluminescent image	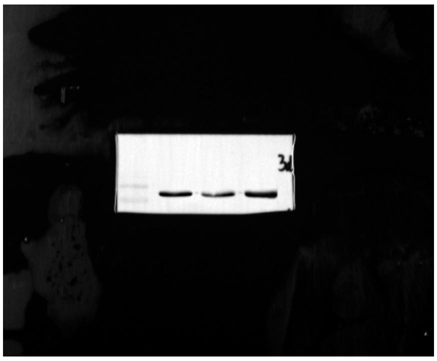<br>Composite image

Figure4F-HT22-β-actin

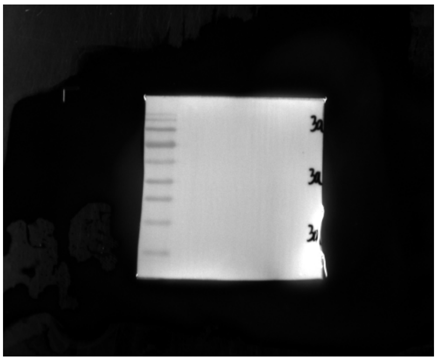	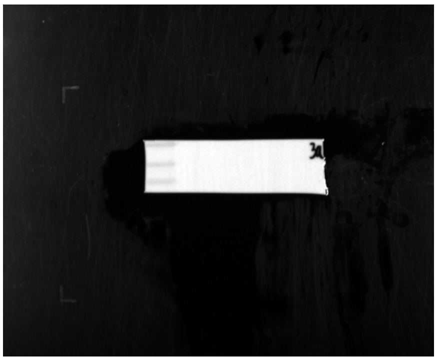

Full membrane image	White light image
Chemiluminescent image	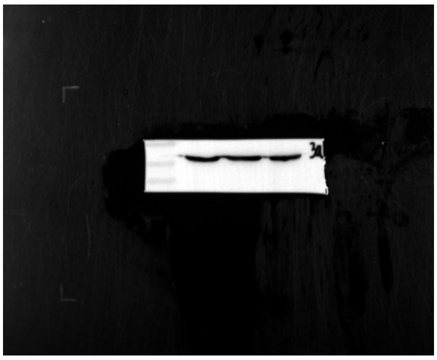 Composite image

Figure5A
Figure5A-Foxm1

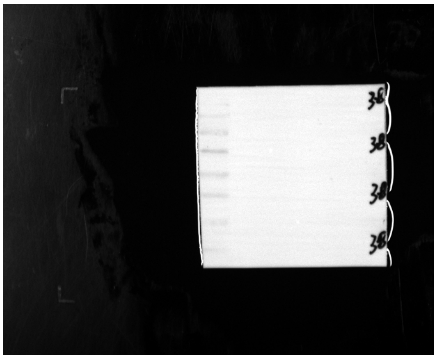 Full membrane image	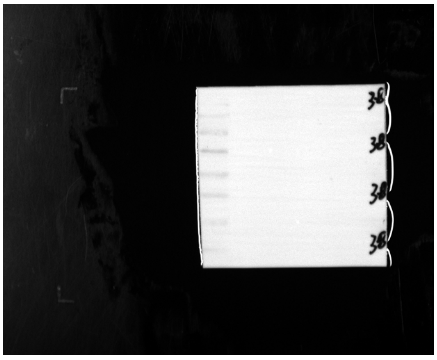 White light image
Chemiluminescent image	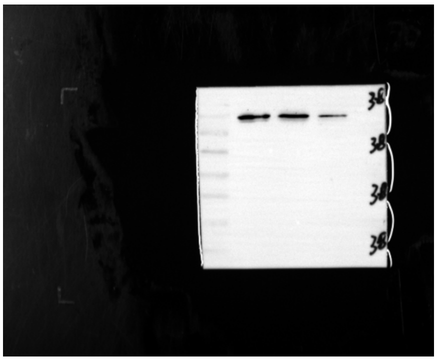 Composite image

Figure5A-β-actin

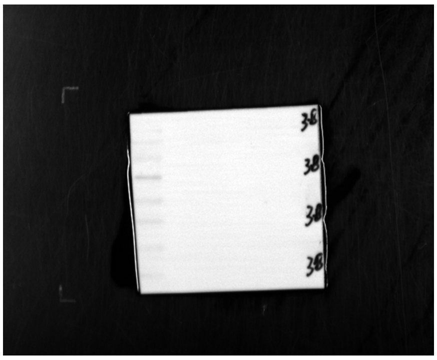<br>Full membrane image	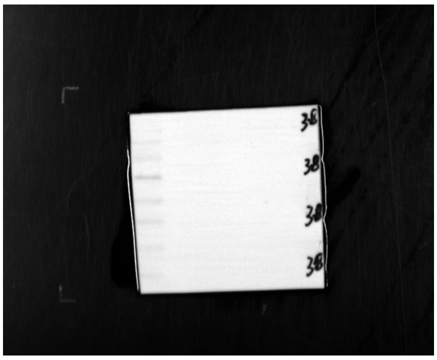<br>White light image
 Chemiluminescent image	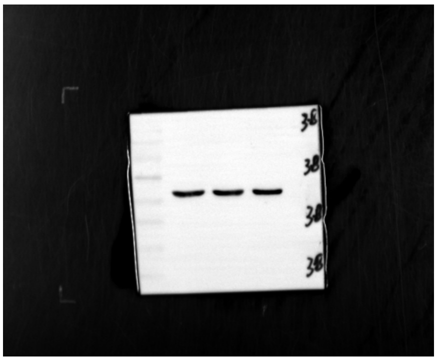<br>Composite image

Figure5B

Figure5B-Foxm1

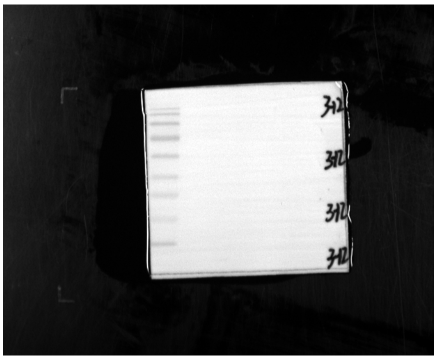<br>Full membrane image	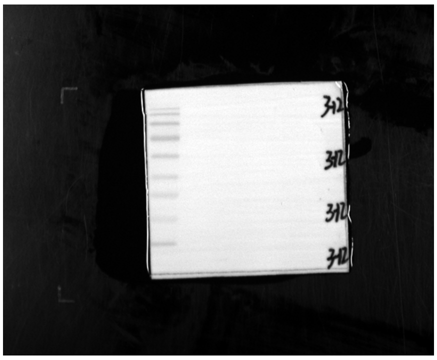<br>White light image
 Chemiluminescent image	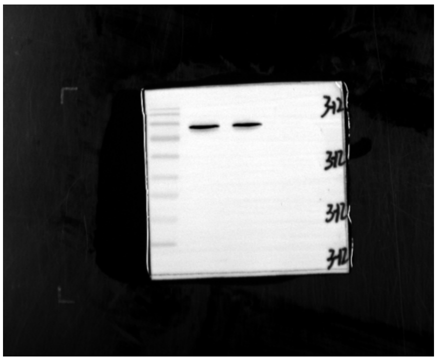<br>Composite image

Figure5C

Figure5C-HT22-Foxm1

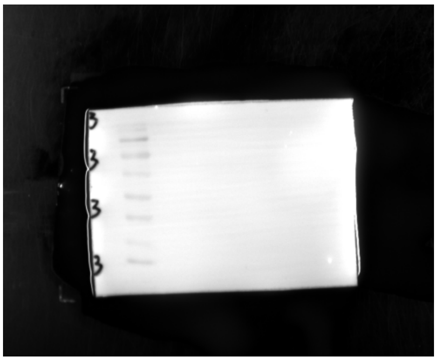 Full membrane image	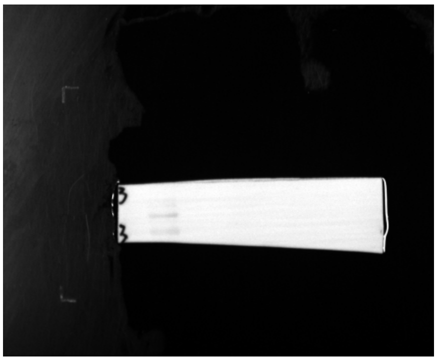 White light image
Chemiluminescent image	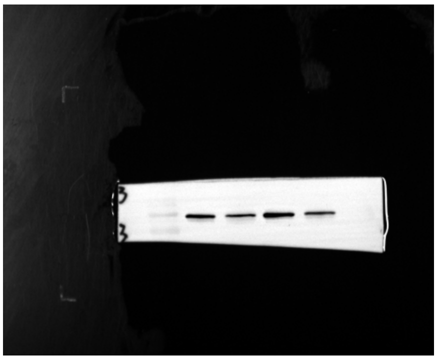 Composite image

Figure5C-HT22-β-actin

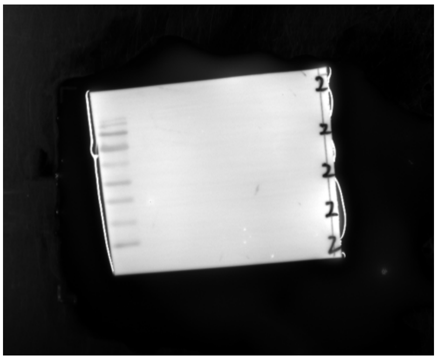 Full membrane image	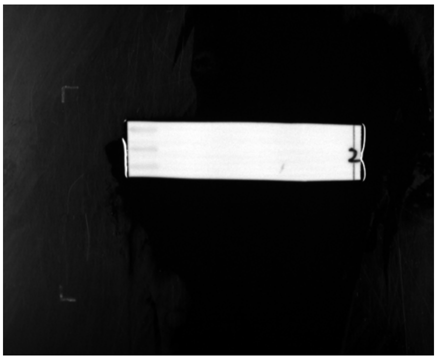 White light image
Chemiluminescent image	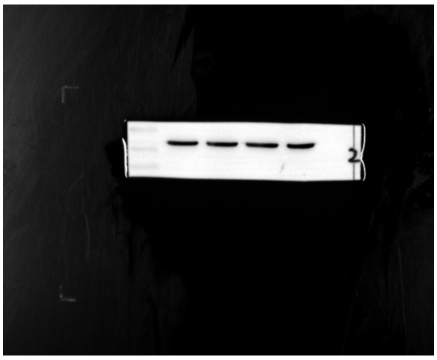 Composite image

Figure5D

Figure5D-BV2-Foxm1

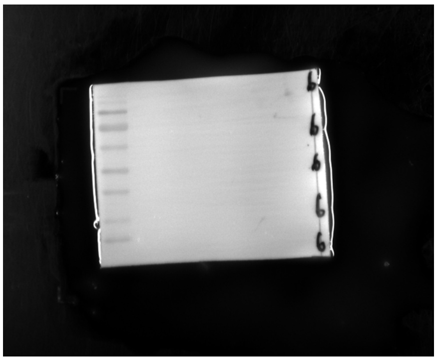 Full membrane image	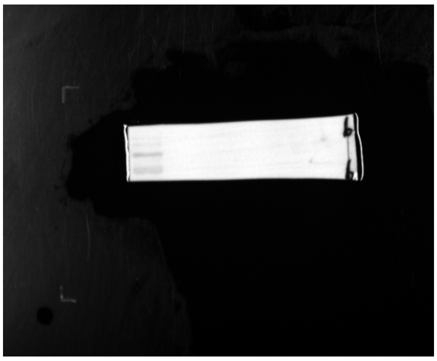 White light image
Chemiluminescent image	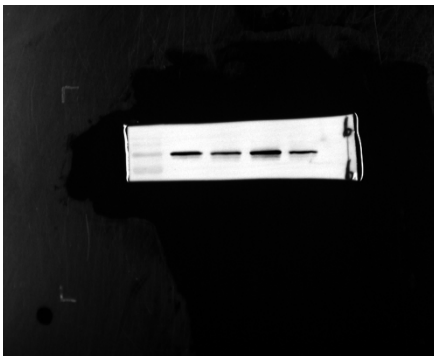 Composite image

Figure5D-BV2-β-actin Figure5D-BV2-Foxm1

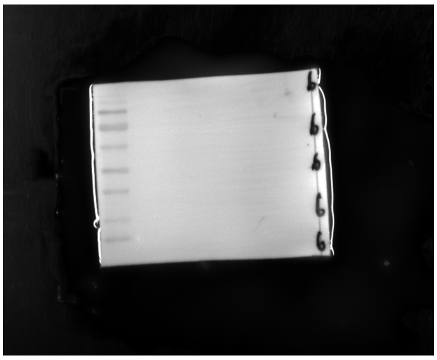 Full membrane image	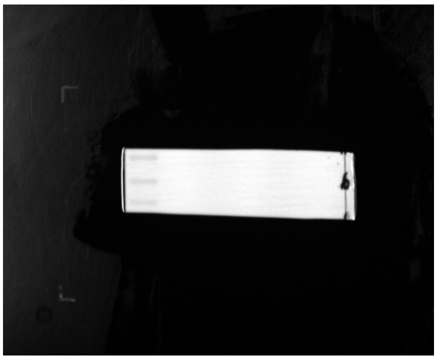 White light image
	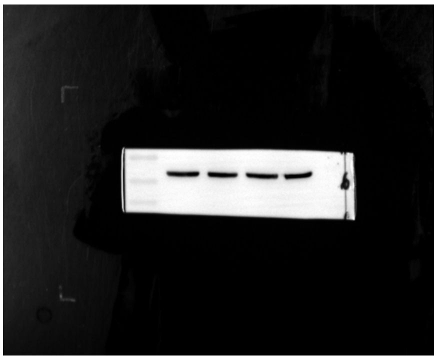

Chemiluminescent image	Composite image

Figure5I

Figure5I-HT22-Bax

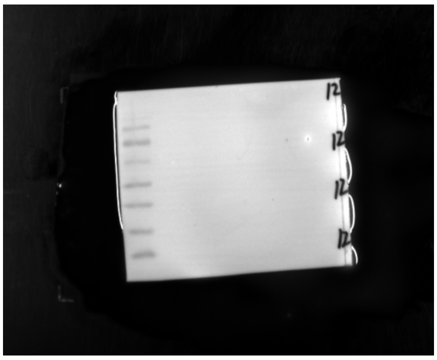<br>Full membrane image	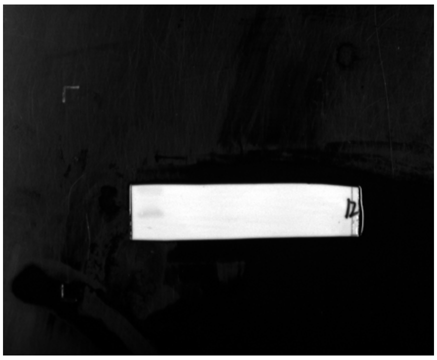<br>White light image
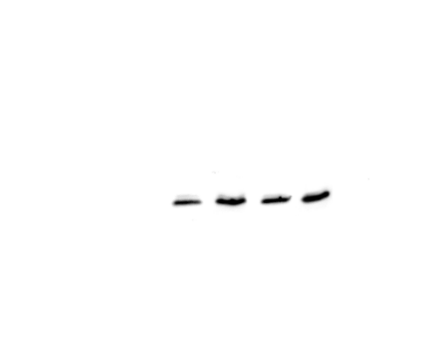<br>Chemiluminescent image	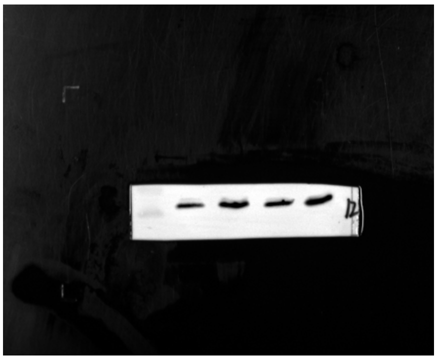<br>Composite image

Figure5I-HT22-Bcl2

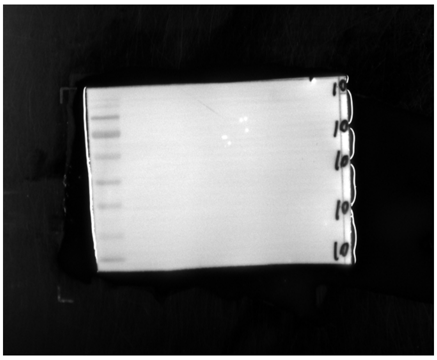<br>Full membrane image	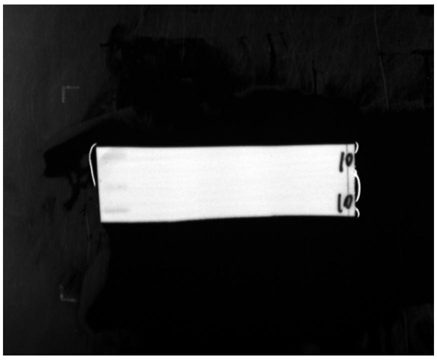<br>White light image

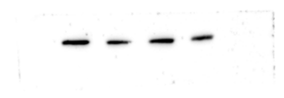<br>Chemiluminescent image	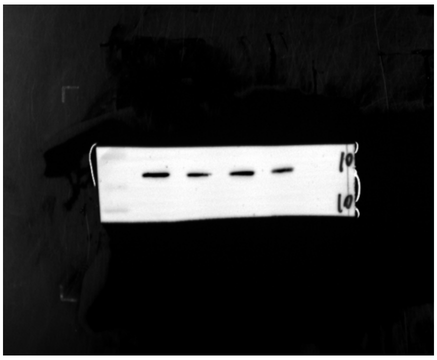<br>Composite image

Figure5I-HT22-Caspase3

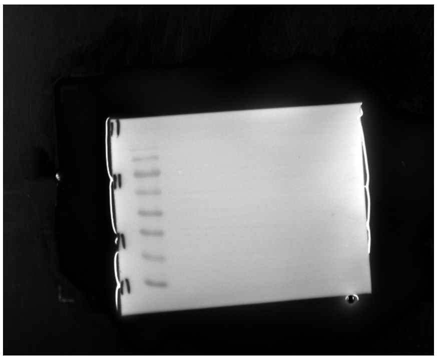<br>Full membrane image	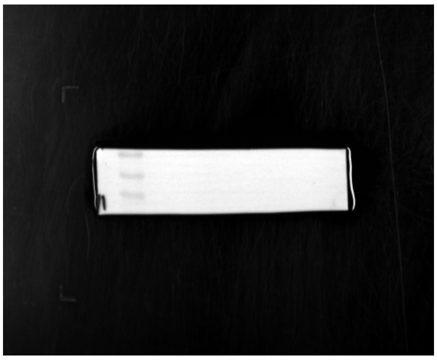<br>White light image
 Chemiluminescent image	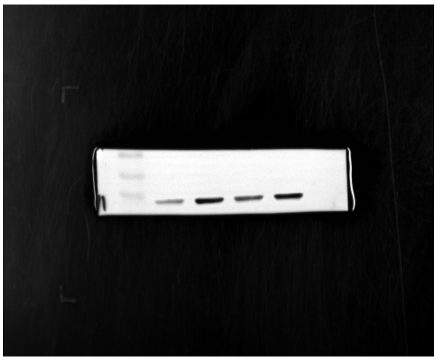<br>Composite image

Figure5I-HT22-BDNF

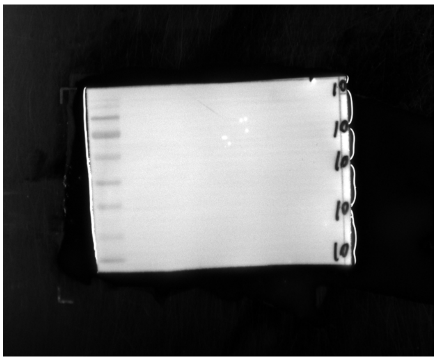<br>Full membrane image	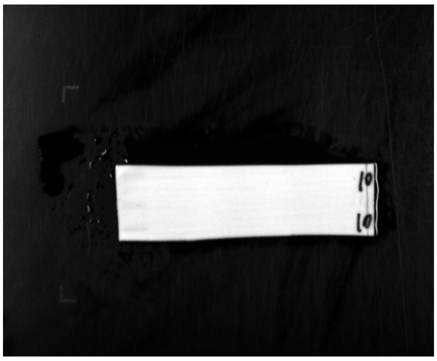<br>White light image

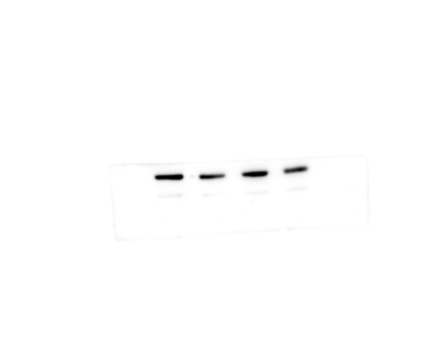 Chemiluminescent image	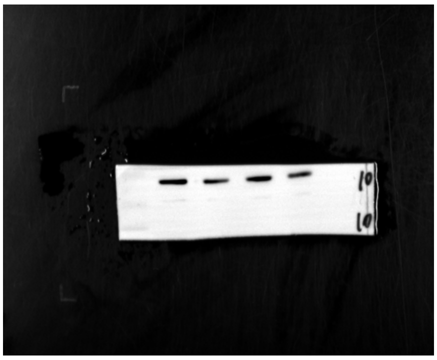 Composite image

Figure5I-HT22-β-actin

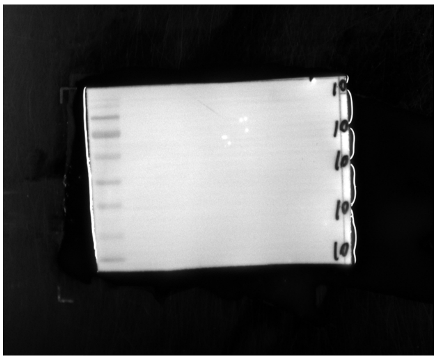 Full membrane image	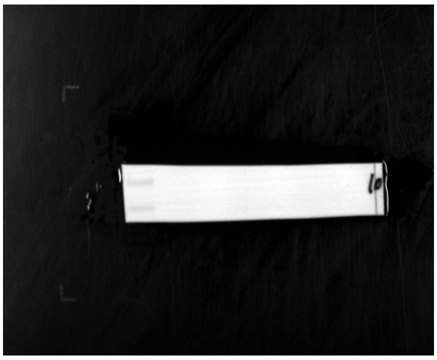 White light image
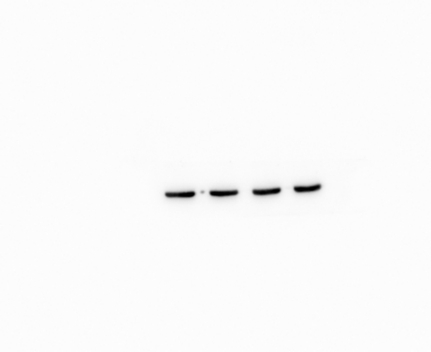 Chemiluminescent image	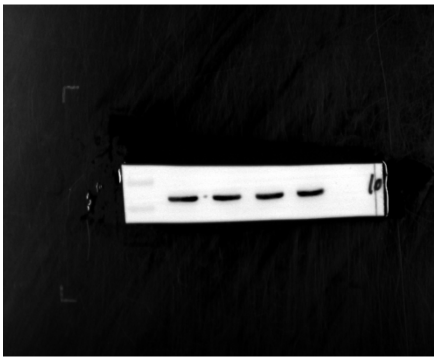 Composite image

Figure5J

Figure5J-BV2-Bax

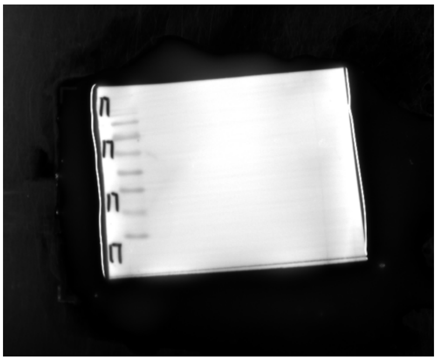	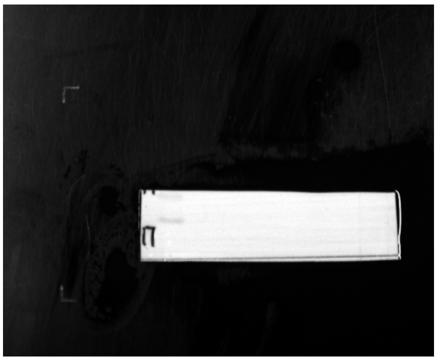

Full membrane image	White light image
Chemiluminescent image	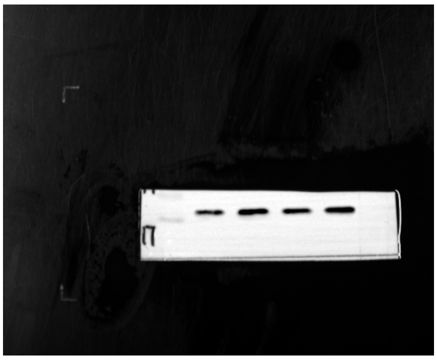 Composite image

Figure5J-BV2-Bcl2

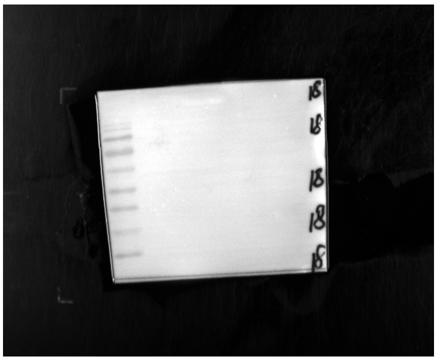 Full membrane image	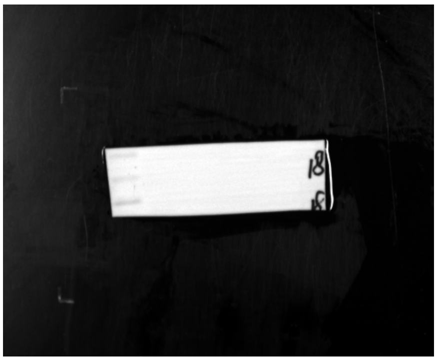 White light image
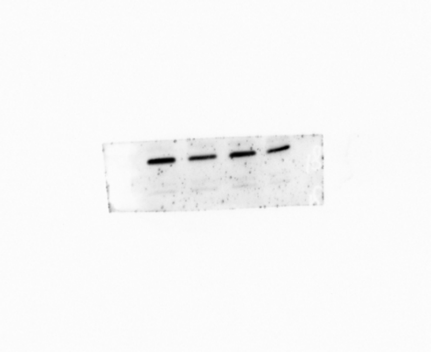 Chemiluminescent image	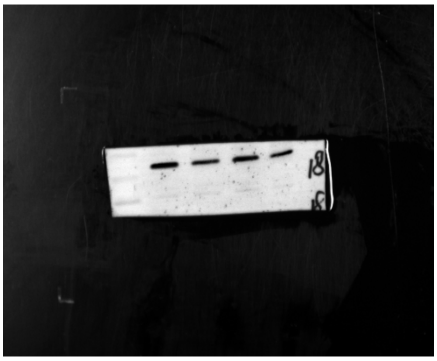 Composite image

Figure5J-BV2-Caspase3

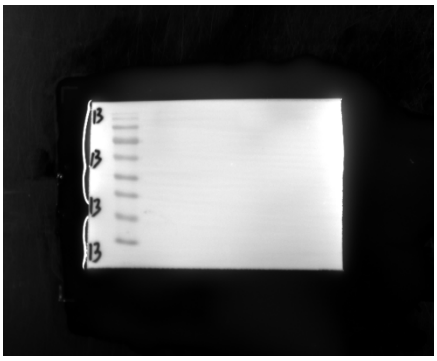	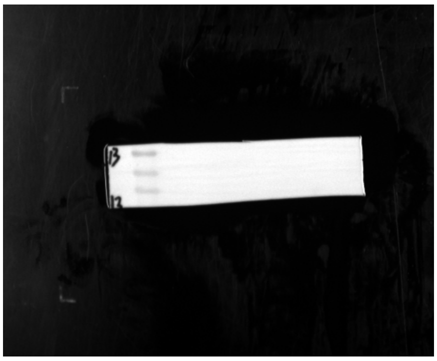

Full membrane image	White light image
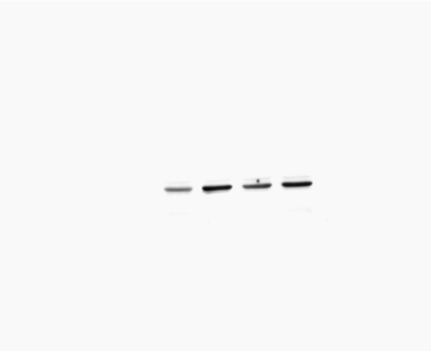	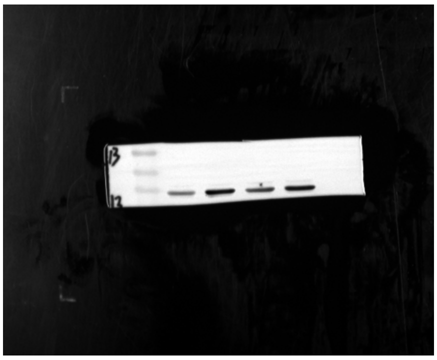
Chemiluminescent image	Composite image

Figure5J-BV2-BDNF

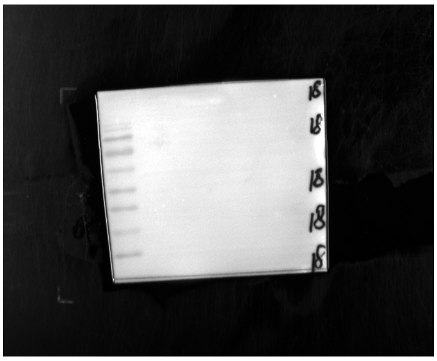	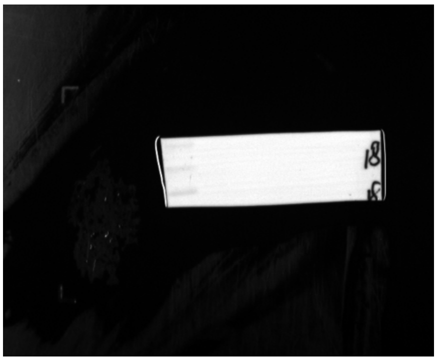
Full membrane image	White light image
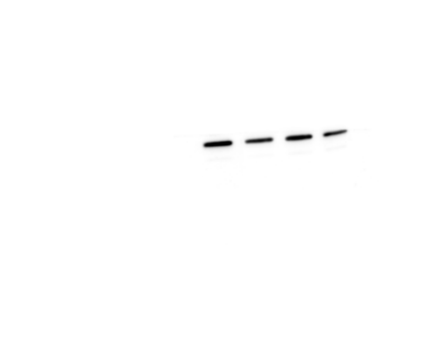	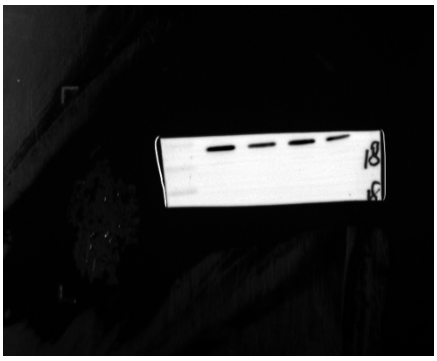
Chemiluminescent image	Composite image

Figure5J-BV2-β-actin

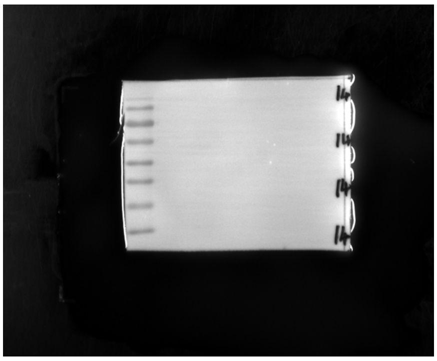	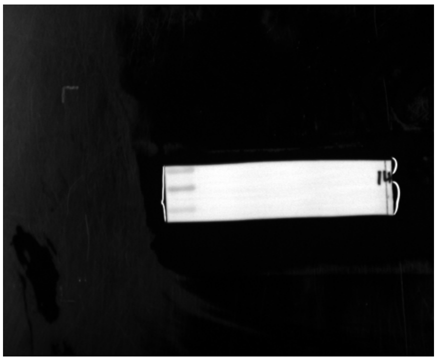

Full membrane image	White light image
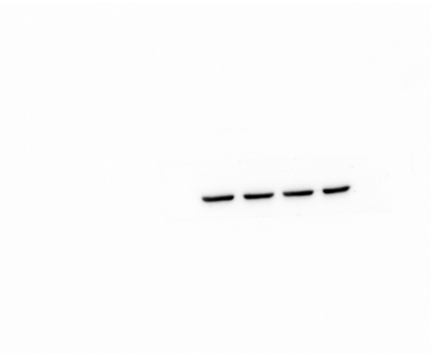	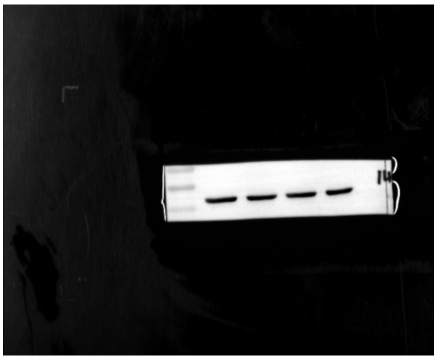
Chemiluminescent image	Composite image
